# Supplementary material for: LiveCellMiner: A new tool to analyze mitotic progression
Source: PLoS One. 2022 Jul 7;17(7):e0270923. doi: 10.1371/journal.pone.0270923 (PMC9262191; doi:10.1371/journal.pone.0270923)
Supplement: S1 Text — (PDF) [file pone.0270923.s001.pdf]

LiveCellMiner: A New Tool to Analyze Mitotic Progression

Daniel Moreno-Andrés[1,*,†], Anuk Bhattacharyya[2], Anja Scheufen[1], Johannes Stegmaier[2,*,†]

1 Institute of Biochemistry and Molecular Cell Biology, Medical School, RWTH Aachen University, Aachen, Germany
2 Institute of Imaging and Computer Vision, RWTH Aachen University, Aachen, Germany

* Correspondence: dmoreno@ukaachen.de, johannes.stegmaier@lfb.rwth-aachen.de †: These authors contributed equally.

S1 Note: Experimental Details

LSM5live Experiments. LSD1 Data Set [1]

HeLa cells expressing H2B-mCherry and α-tubulin-EGFP were transfected with the indicated siRNA oligonucleotides and seeded in eight-well µ-slide chambers (Ibidi). Starting at 30 h post-transfection, cells were imaged using a Plan-Apochromat 10× NA 0.45 objective and a 561-nm diode laser on a LSM5 live confocal microscope (Zeiss) equipped with a heating and CO2 incubation system (Ibidi). ZEN software (Zeiss) was used to acquire images from five 7.5-µm-spaced optical z-sections at various xy positions every 3 min. Single position *.ome files were generated from the maximum intensity projections in ZEN and converted into image sequences with Fiji software.

LSM5live Experiments. RecQL4 Data Set [2]

HeLa cells expressing H2B-mCherry and EGFP-α-tubulin were transfected with the indicated siRNA oligonucleotides in eight-well µ-slide chambers (Ibidi) and, after 24 h, were imaged for 48 h in a LSM 5 live confocal microscope (Zeiss) equipped with a heating and CO2 incubation system (Ibidi). Seven 3.6-µm-spaced optical z-sections at various positions every 3 min were acquired with a Plan-Apochromat 20× NA 0.8 objective and a 488-nm and 561-nm diode lasers controlled by ZEN software. For the analysis, maximum intensity projections in Z were generated in ZEN for every position and converted into temporal image sequences with the free licensed AxioVision software (LE64; V4.9.1.0).

LSM710 Experiments. CTRL vs PP2A Data Set (Unpublished)

HeLa cells expressing H2B-mCherry were transfected with the indicated siRNA oligonucleotides in eight-well µ-slide chambers (Ibidi) and, after 24 h, were imaged for 48 h in a LSM 710 confocal microscope (Zeiss) equipped with an Incubator XL S1(Zeiss). Seven 2.6-µm-spaced optical z-sections at various positions every 3 min were acquired with a Plan-Apochromat 20× NA 0.8 objective and a 488-nm Argon and 561-nm DPSS lasers controlled by ZEN software (Zeiss). For the analysis, maximum intensity projections in Z were generated for every position and converted into temporal image sequences in ZEN 2.3 software (Zeiss).

Nikon Experiments. VPS72, INO80, SRCAP, EP400 and H2A.Z. Data Set [3]

HeLa cells expressing H2B-mCherry were transfected with the indicated siRNA oligonucleotides in eight-well μ-slide chambers (Ibidi) and, after 48 h, were imaged for 48 h with the widefield module of a Ti2 Eclipse (Nikon) equipped with a LED light engine SpectraX (Lumecor) and GFP/mCherry filter sets, a Plan-Apochromat 10x NA 0.5 objective and environmental control system (Ibidi). Elements software (Nikon) was used to perform fluorescence multi-position imaging every three minutes and the subsequent conversion to image sequences.

Nikon Experiments. RPE cells. Data Set [3]

RPE cells expressing H2B-mCherry were transfected with 20nM of the indicated siRNA oligonucleotides in eight-well µ-slide chambers (Ibidi) and, after 48 h, were imaged for 48 h with the widefield module of a Ti2 Eclipse (Nikon) equipped with a LED light engine SpectraX (Lumecor) and GFP/mCherry filter sets, a Plan-Apochromat 20x NA 0.75 air objective and environmental control system (Ibidi). Elements software (Nikon) was used to perform fluorescence multi-position imaging every three minutes and the subsequent conversion to image sequences.

Screening Data Set by Hériché *et al.* [4]

The screening data set is publicly available at Image Data Resource (IDR) (`https://idr.openmicroscopy.org/webclient/?show=screen-102`). HeLa cells stably expressing HIST1H2BJ-mCherry and LMNA-eGFP in each well of siRNA-coated 96-well plates. The images were acquired with an Olimpus IX-81 automated epifluorescence microscope with a 20$\times$ objective and a time interval of 8.5 min for 44 h. Four independent replicates were acquired for each siRNA treatment.

References

1. Schooley A, Moreno-Andres D, De Magistris P, Vollmer B, Antonin W. The Lysine Demethylase LSD1 is Required for Nuclear Envelope Formation at the End of Mitosis. Journal of Cell Science. 2015;128(18):3466–77.

2. Yokoyama H, Moreno-Andres D, Astrinidis SA, Hao Y, Weberruss M, Schellhaus AK, et al. Chromosome Alignment Maintenance Requires the MAP RECQL4, Mutated in the Rothmund-Thomson Syndrome. Life Science Alliance. 2019;2(1).

3. Moreno-Andres D, Yokoyama H, Scheufen A, Holzer G, Lue H, Schellhaus AK, et al. VPS72/YL1-Mediated H2A.Z Deposition Is Required for Nuclear Reassembly after Mitosis. Cells. 2020;9(7).

4. Hériché JK, Lees JG, Morilla I, Walter T, Petrova B, Roberti MJ, et al. Integration of Biological Data by Kernels on Graph Nodes Allows Prediction of New Genes Involved in Mitotic Chromosome Condensation. Molecular Biology of the Cell. 2014;25(16):2522–2536.
