# Supplementary figures and images for: LiveCellMiner: A new tool to analyze mitotic progression (part 1 of 2)
Source: PLoS One. 2022 Jul 7;17(7):e0270923. doi: 10.1371/journal.pone.0270923 (PMC9262191; doi:10.1371/journal.pone.0270923)

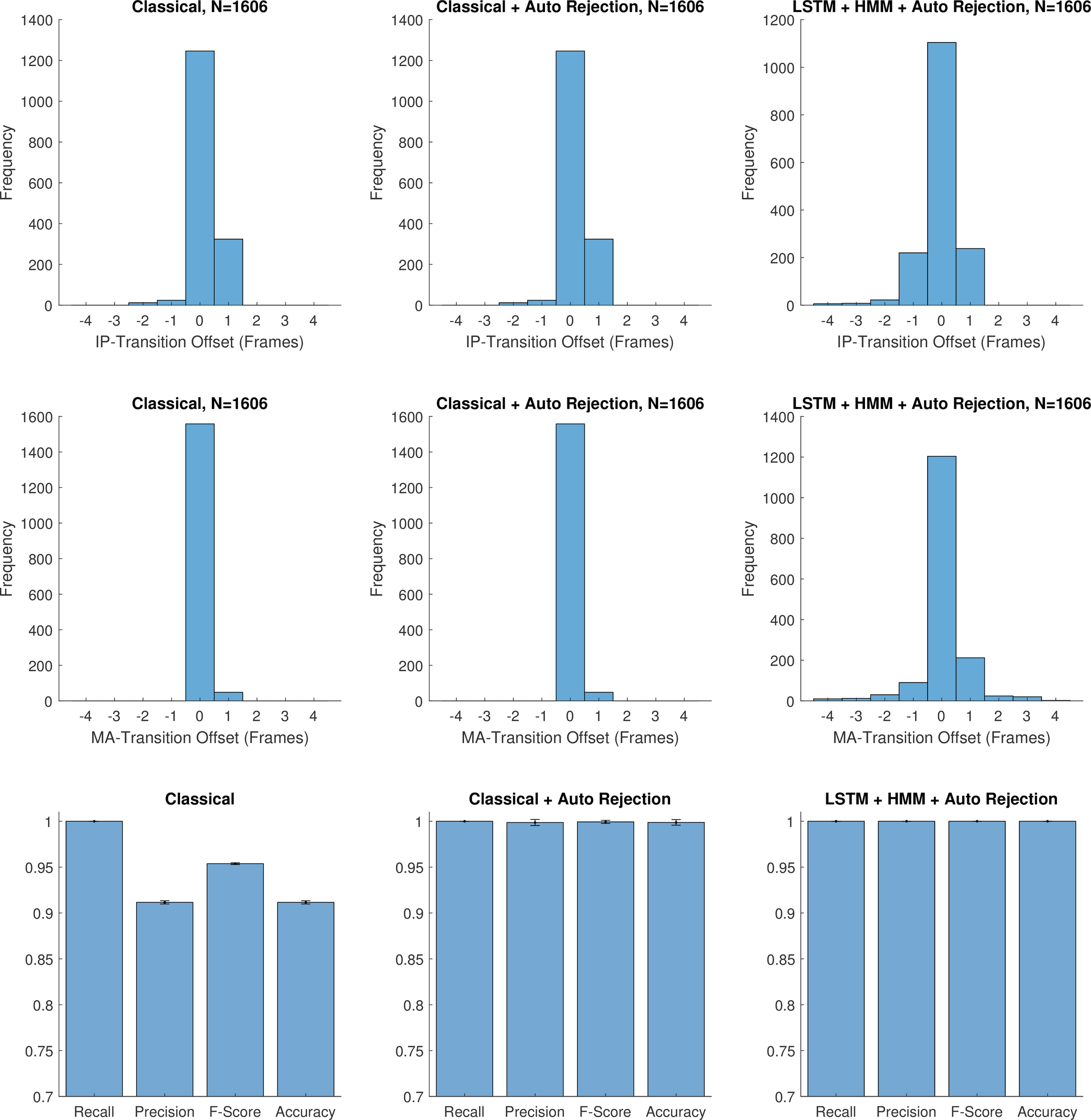

Supplement: S1 Fig — The manually annotated data comprise three experiments with two positions each (20× objective, physical spacing 0.415μm, 3 minute time intervals and N = 832 trajectories in total). Columns show the results of the three different synchronization methods (Classical, Classical+Auto Rejection and LSTM+HMM+Auto Rejection) as described in the main text. The first two rows show histograms of the frame offset of the automatically identified synchronization time points with respect to a manually annotated ground truth (IP: interphase to prophase transition and MA: metaphase to anaphase transition). For instance, a value of 1 indicates that the synchronization time point is set one frame too late and 0 indicates a perfect match. The last row quantifies precision, recall, f-score and accuracy of the auto rejection module that is intended to discard erroneous tracks. Validation was performed using a 6-fold cross validation by retraining the LSTM-based classifier on each split and testing the classifier on each of the remaining test data. (TIF) [file pone.0270923.s002.tif]

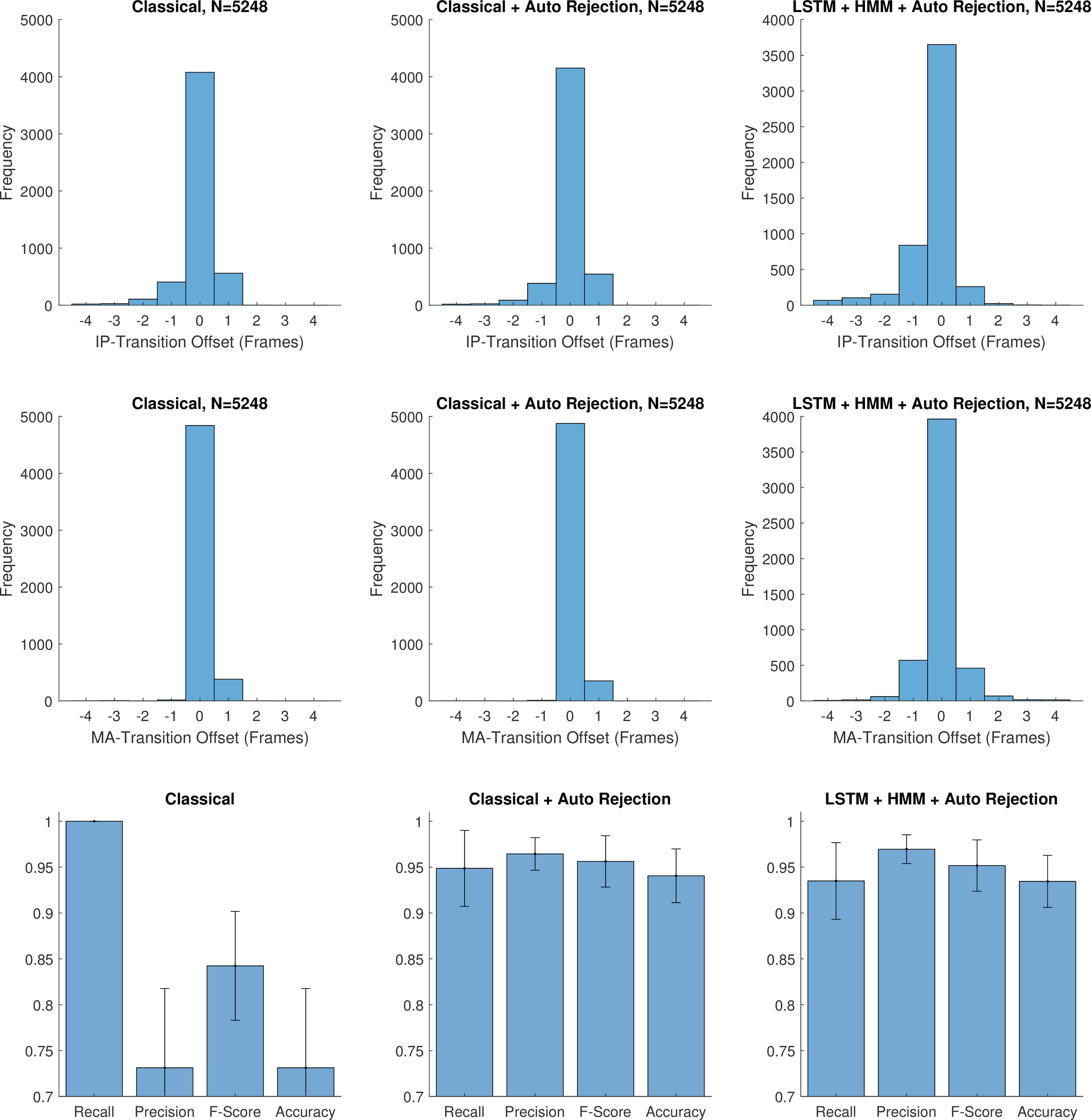

Supplement: S2 Fig — The manually annotated data comprise three experiments with two positions each (10× objective, physical spacing 0.65μm, 3 minute time intervals and N = 832 trajectories in total). Columns show the results of the three different synchronization methods (Classical, Classical+Auto Rejection and LSTM+HMM+Auto Rejection) as described in the main text. The first two rows show histograms of the frame offset of the automatically identified synchronization time points with respect to a manually annotated ground truth (IP: interphase to prophase transition and MA: metaphase to anaphase transition). For instance, a value of 1 indicates that the synchronization time point is set one frame too late and 0 indicates a perfect match. The last row quantifies precision, recall, f-score and accuracy of the auto rejection module that is intended to discard erroneous tracks. Validation was performed using a 6-fold cross validation by retraining the LSTM-based classifier on each split and testing the classifier on each of the remaining test data. (TIF) [file pone.0270923.s003.tif]

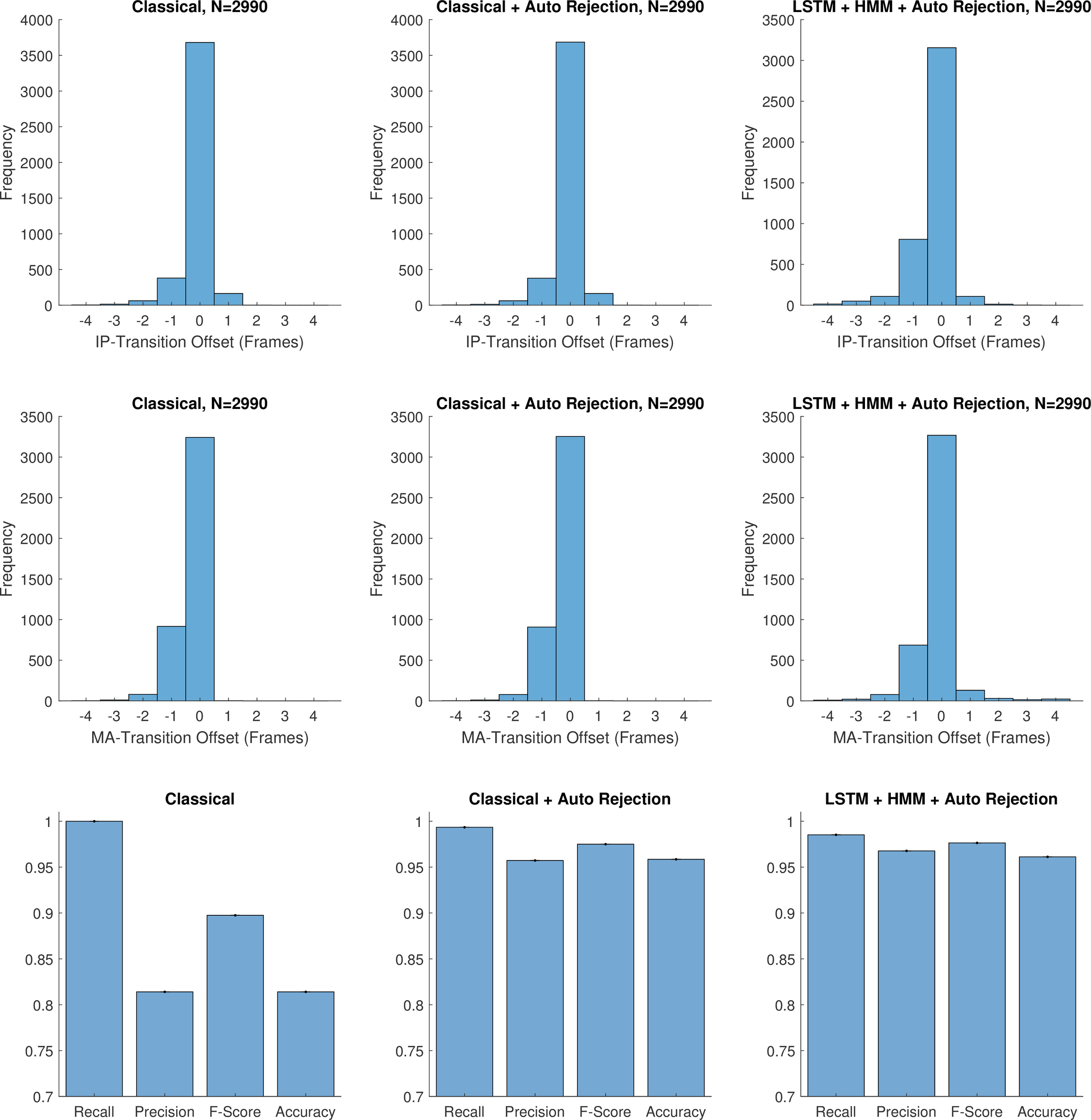

Supplement: S3 Fig — The manually annotated data comprise three experiments with 16 positions each (10× objective, physical spacing 0.656μm, 3 minute time intervals and N = 5878 trajectories in total). Columns show the results of the three different synchronization methods (Classical, Classical+Auto Rejection and LSTM+HMM+Auto Rejection) as described in the main text. The first two rows show histograms of the frame offset of the automatically identified synchronization time points with respect to a manually annotated ground truth (IP: interphase to prophase transition and MA: metaphase to anaphase transition). For instance, a value of 1 indicates that the synchronization time point is set one frame too late and 0 indicates a perfect match. The last row quantifies precision, recall, f-score and accuracy of the auto rejection module that is intended to discard erroneous tracks. Validation was performed by training on 1328 trajectories that were evenly distributed among all positions and by applying it to a remaining set of 2990 trajectories. (TIF) [file pone.0270923.s004.tif]

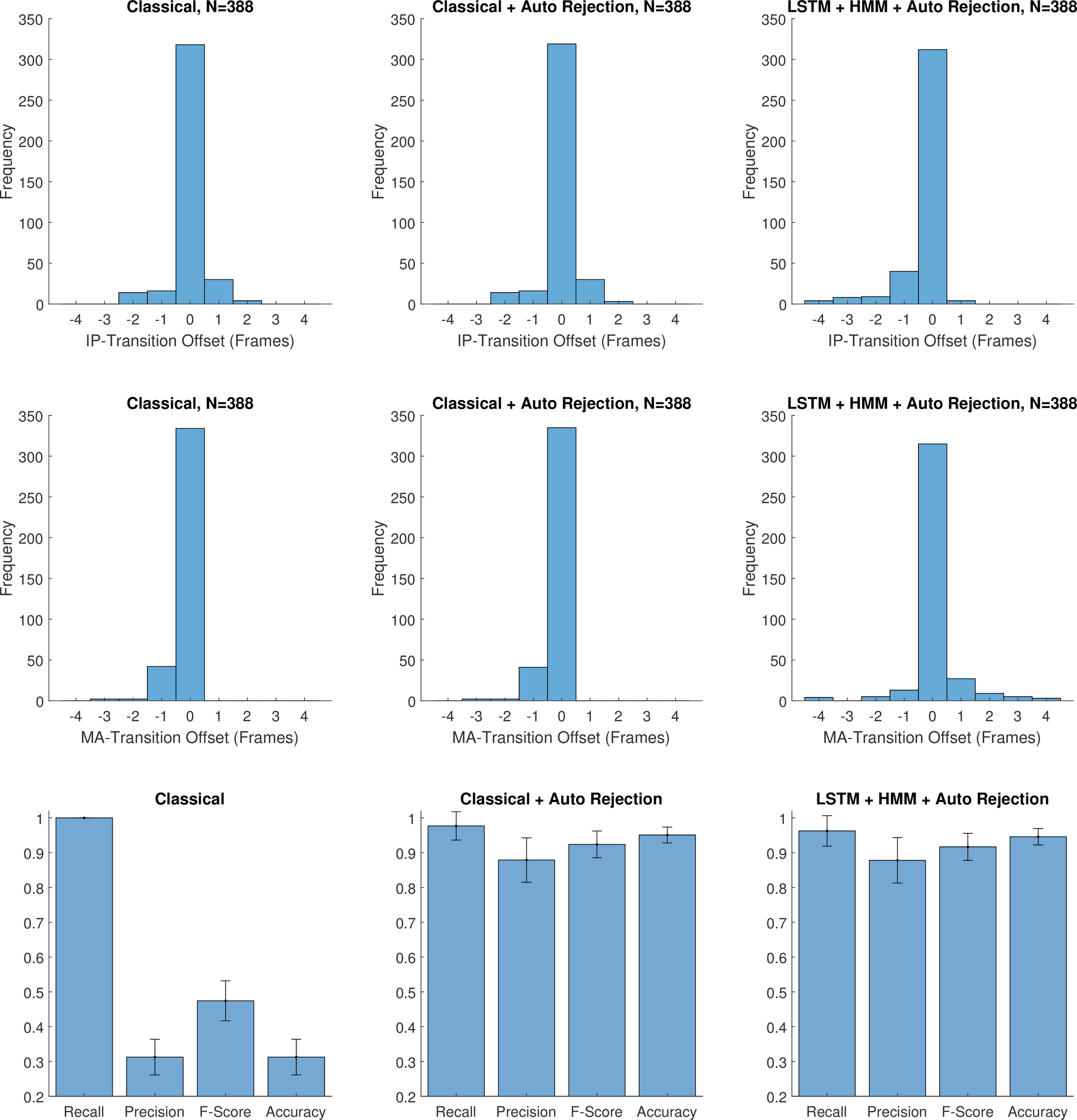

Supplement: S4 Fig — The manually annotated data comprise one experiment with 6 positions (Nikon microscope, 20x objective, physical spacing 0.33μm, 3 minute time intervals and N = 388 trajectories in total). Columns show the results of the three different synchronization methods (Classical, Classical+Auto Rejection and LSTM+HMM+Auto Rejection) as described in the main text. The first two rows show histograms of the frame offset of the automatically identified synchronization time points with respect to a manually annotated ground truth (IP: interphase to prophase transition and MA: metaphase to anaphase transition). For instance, a value of 1 indicates that the synchronization time point is set one frame too late and 0 indicates a perfect match. The last row quantifies precision, recall, f-score and accuracy of the auto rejection module that is intended to discard erroneous tracks. Validation was performed using a 6-fold cross validation by retraining the LSTM-based classifier on each split and testing the classifier on each of the remaining test data. (TIF) [file pone.0270923.s005.tif]

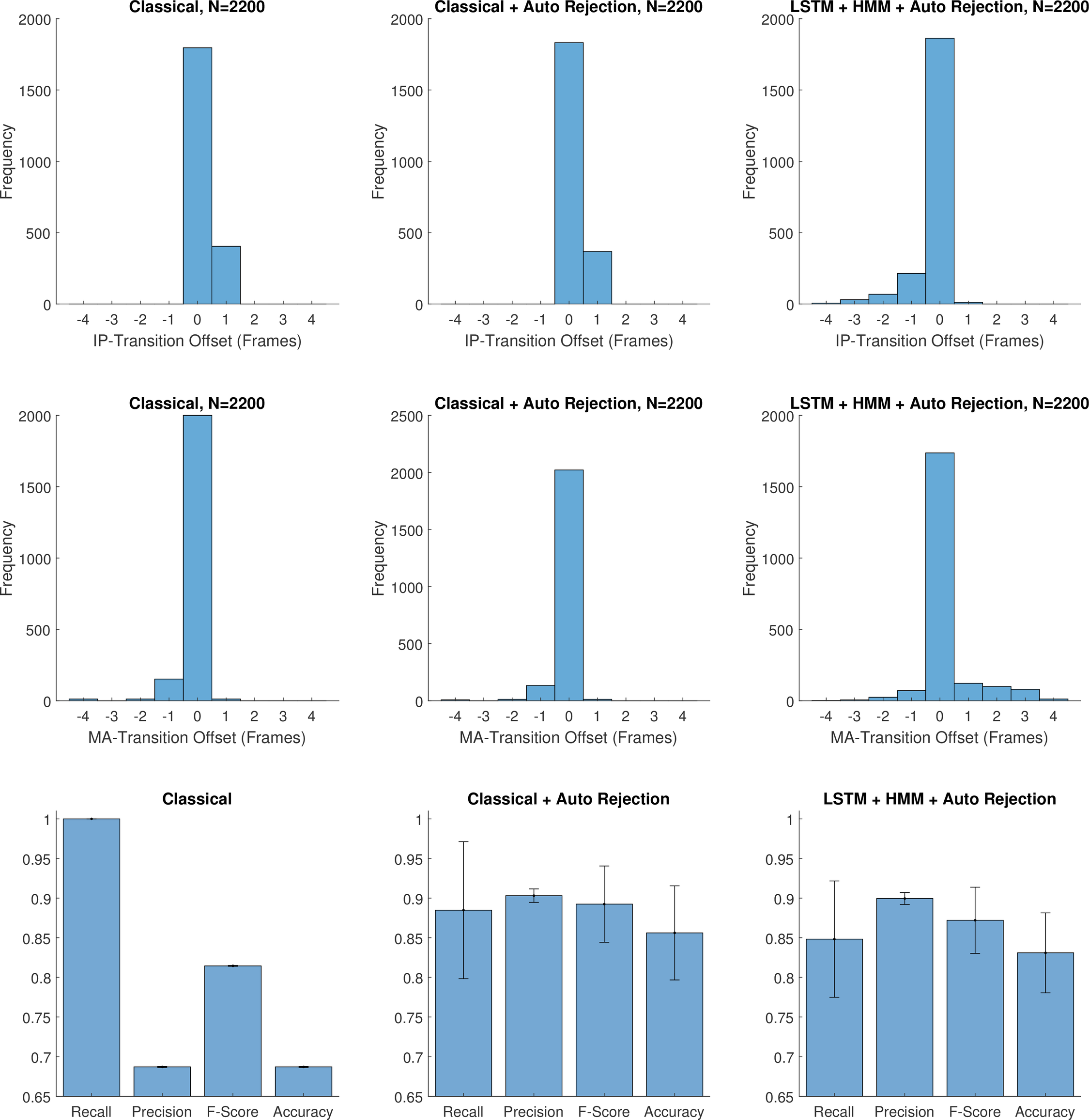

Supplement: S5 Fig — The manually annotated data comprise twelve experiments with 4 positions each (automated epifluorescence microscope, 20× objective, physical spacing 0.32μm, 8.5 minute time intervals and N = 2200 trajectories in total). Columns show the results of the three different synchronization methods (Classical, Classical+Auto Rejection and LSTM+HMM+Auto Rejection) as described in the main text. The first two rows show histograms of the frame offset of the automatically identified synchronization time points with respect to a manually annotated ground truth (IP: interphase to prophase transition and MA: metaphase to anaphase transition). For instance, a value of 1 indicates that the synchronization time point is set one frame too late and 0 indicates a perfect match. The last row quantifies precision, recall, f-score and accuracy of the auto rejection module that is intended to discard erroneous tracks. Validation was performed using a 6-fold cross validation by retraining the LSTM-based classifier on each split and testing the classifier on each of the remaining test data. (TIF) [file pone.0270923.s006.tif]

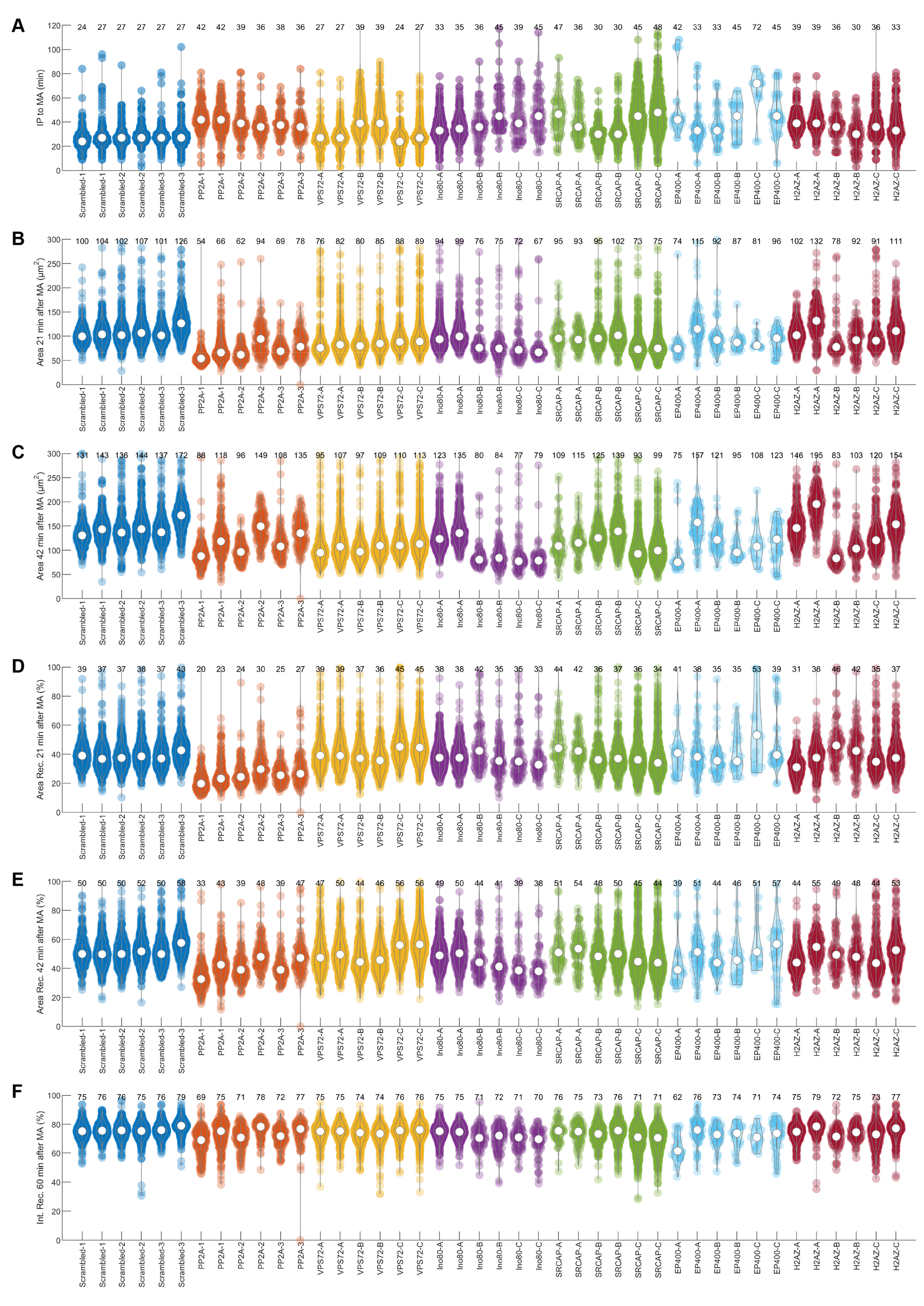

Supplement: S6 Fig — The different panels show violin plots of the IP to MA duration in minutes (A), the area at 21 and 42 minutes after anaphase onset (B, C), the area recovery compared to the level at interphase in % at 21 and 42 minutes after the anaphase onset (D, E) as well as a combined recovery measure comprised of area, minor axis length, mean intensity and intensity standard deviation at 60 minutes after anaphase onset (F). See S2 Table for a more detailed description of the individual features. Numbers above each violin are the respective median values. (TIF) [file pone.0270923.s007.tif]

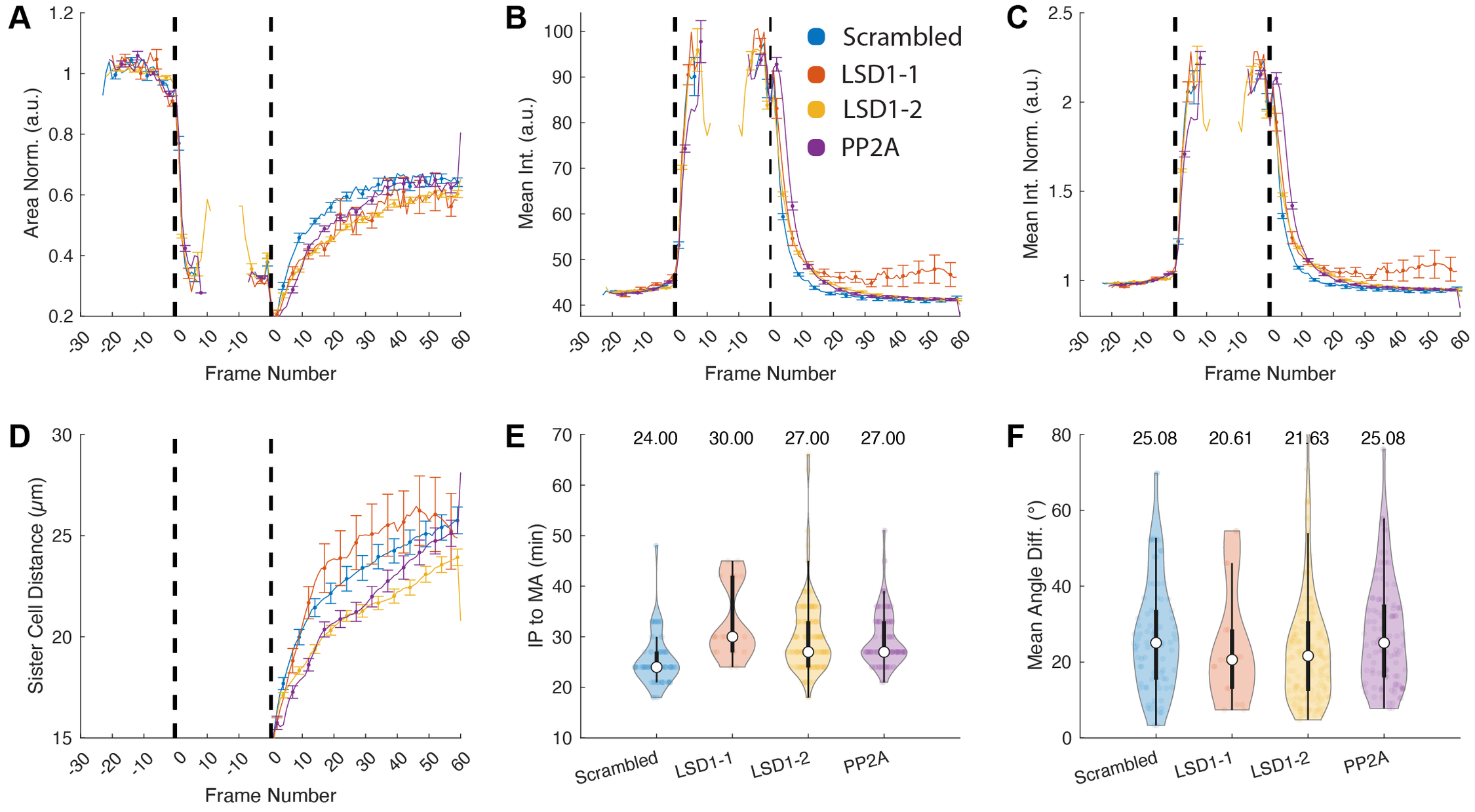

Supplement: S7 Fig — Images were acquired with a confocal microscope (LSM5L, 10×, 0.656μm/pixel). We compare control (Scrambled), LSD1-1, LSD1-2 and PP2A knockdown cells. The basic features involve the normalized area (A), the mean intensity (B), the normalized mean intensity (C) and the sister cell displacement (D). The violin plots show the duration between interphase-prophase and metaphase-anaphase transition in minutes (E) and the mean orientation angle difference in degrees (F). The selection was constrained to cells exhibiting an interphase mean intensity in the range of 40−46, which yielded a set of NScrambled = 84, NLSD1-1 = 18, NLSD1-2 = 190, NPP2A = 106 cells. (TIF) [file pone.0270923.s008.tif]

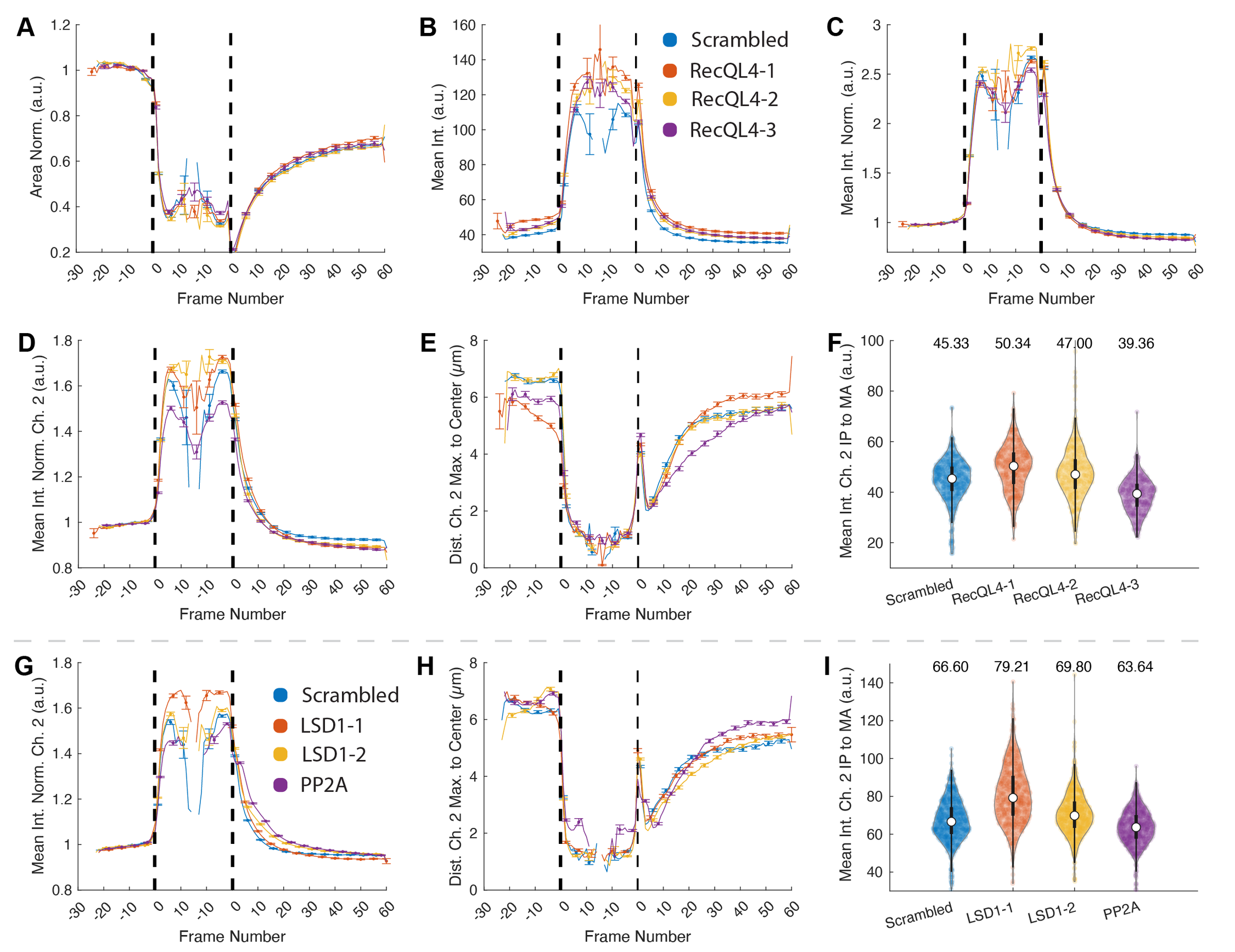

Supplement: S8 Fig — Panels (A)-(F) show control (Scrambled) vs. RecQL4-1, RecQL4-2 and RecQL4-3, whereas panels (G)—(I) show control (Scrambled) vs. LSD1-1, LSD1-2 and PP2A knockdown cells. The features involve the normalized area (A), the mean intensity (B), the normalized mean intensity (absolute intensity values divided by the interphase mean intensity of each cell, C). Panels (D-I) exemplify features that were extracted from the second fluorescence channel and include the normalized mean intensity (D,G), the distance of the intensity maximum to the segmentation centroid (E,H) and the average mean intensity between the IP and MA transitions (F,I). Images of panels (A)-(F) above the dashed line were acquired with a confocal microscope (LSM5L, 20X, 0.656μm/pixel). The plots combine extracted trajectories from three independent repeats with a total number of Nscrambled = 1094, NRecQL4-1 = 814, NRecQL4-3 = 842, NRecQL4-4 = 786 cells. Images of panels (G)-(I) below the dashed line were acquired with a confocal microscope (LSM5L, 10×, 0.656μm/pixel). The plots combine extracted trajectories from three independent repeats with a total number of NScrambled = 1262, NLSD1-2 = 970, NLSD1-6 = 1332, NPP2A = 1198 cells. See S2 and S3 Tables for details on the depicted features. (TIF) [file pone.0270923.s009.tif]

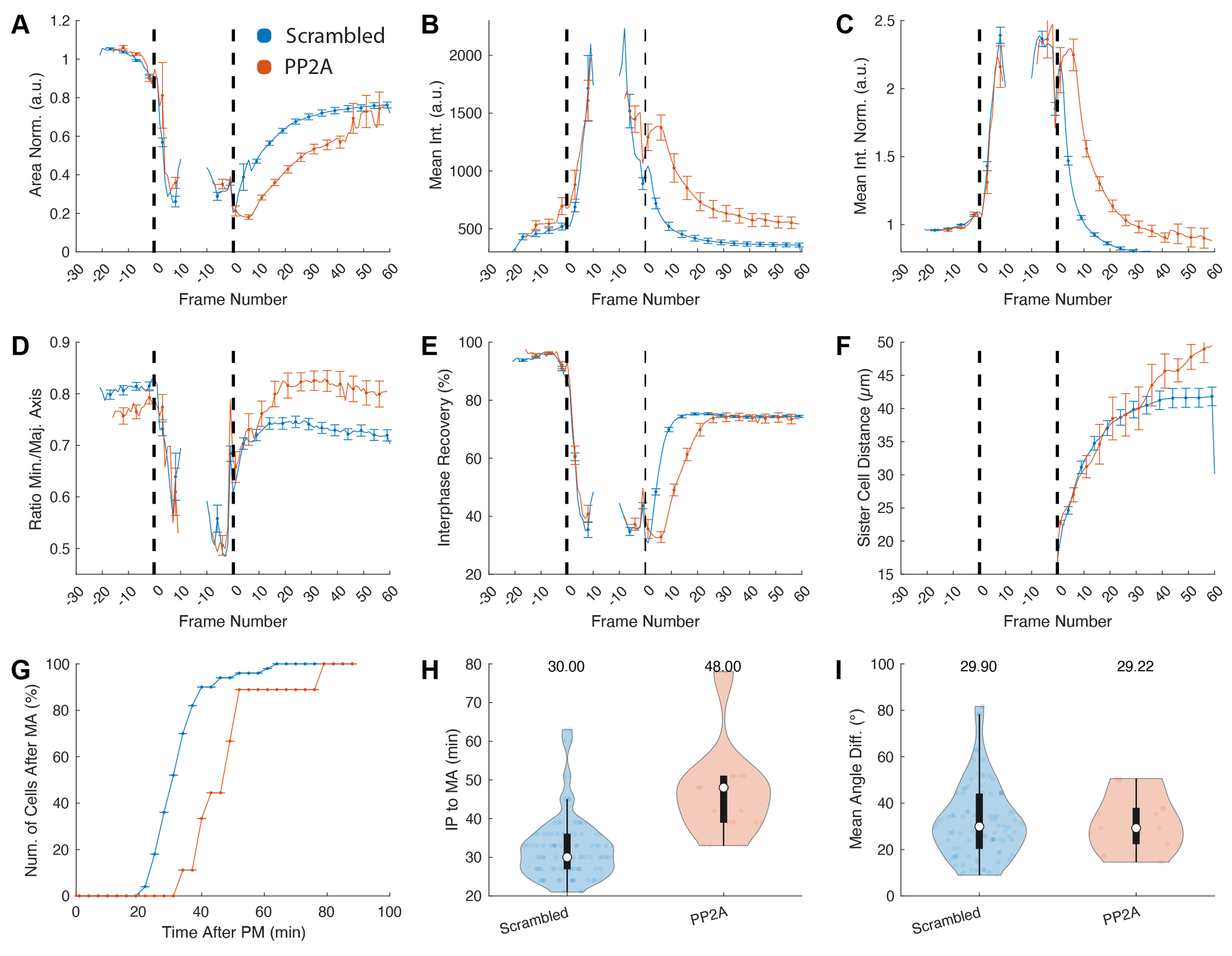

Supplement: S9 Fig — Images were acquired 48 h post transfection with with 20nM siRNA by the widefield module of a Ti2 Eclipse (Nikon) equipped with a LED light engine SpectraX (Lumecor) and GFP/mCherry filter sets, a Plan-Apochromat 20x NA 0.75 and scaling 0.33μm/pixel. We compare the quantitation of features as in Fig 5 for control (Scrambled) and PP2A knockdown. The plots combine extracted trajectories from NScrambled = 104 and NPP2A = 20 cells. See S2 and S3 Tables for details on the depicted features. (TIF) [file pone.0270923.s010.tif]

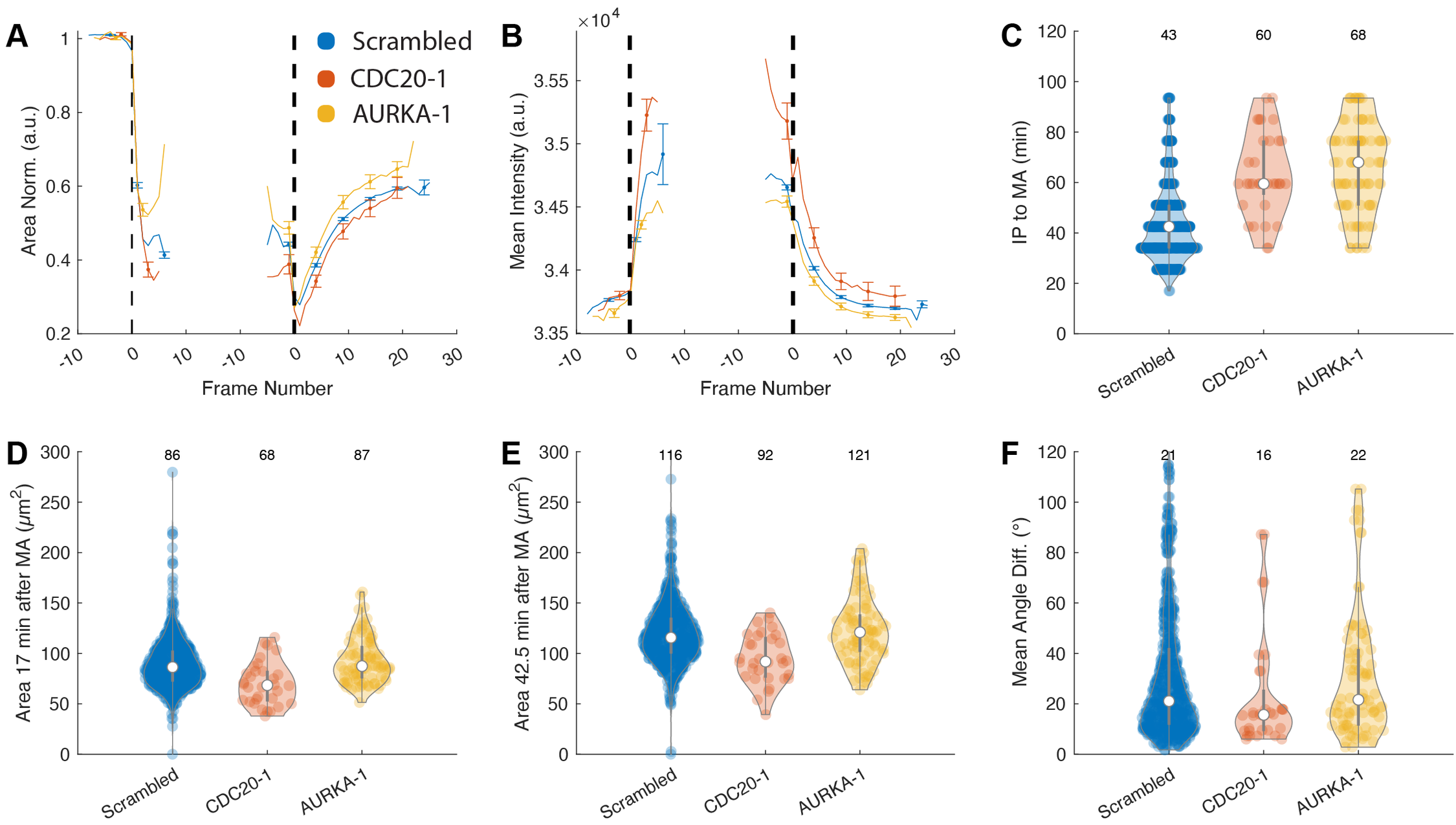

Supplement: S10 Fig — This data set is publicly available at Image Data Resource (IDR) (https://idr.openmicroscopy.org/webclient/?show=screen-102). There, HeLa cells stably expressing HIST1H2BJ-mCherry and LMNA-eGFP were cultured in siRNA-coated 96-well plates. The images were acquired with an Olympus IX-81 automated epifluorescence microscope with a 20× objective, physical spacing 0.32μm and a time interval of 8.5 min for 44 h. Four independent replicates were acquired for each siRNA treatment. The plots show pooled measures from 48 scrambled-, 24 siCDC20- and 4 AurKA-1- siRNA treated positions (NScrambled = 668, NCDC20 = 32, NAurKA-1 = 86). See S2 and S3 Tables for details on the depicted features. (TIF) [file pone.0270923.s011.tif]

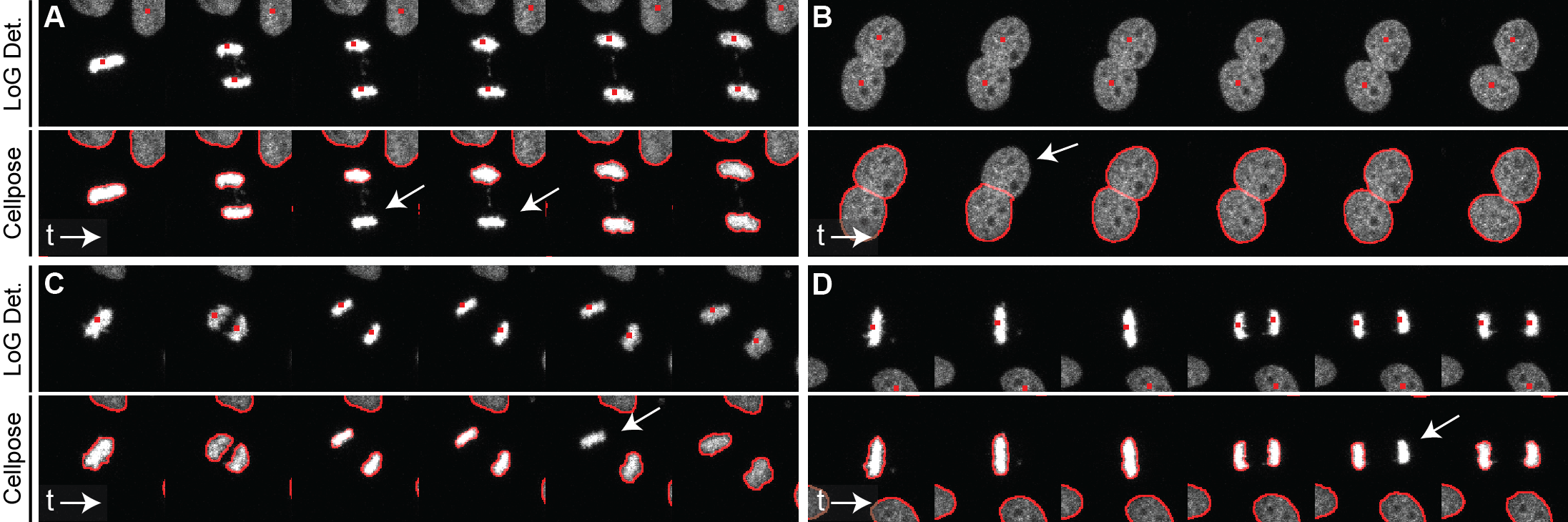

Supplement: S11 Fig — Cellpose provides highly accurate nuclei segmentation for most of the cells. However, in some rare cases (e.g., in late anaphase), cells tend to flicker and remain undetected for one or more frames. To prevent interrupted tracks for such misdetections, LiveCellMiner provides a fallback option on classical image analysis methods and uses a LoG-based nucleus detection coupled with a classical binary threshold and watershed-based segmentation as detailed in the main text. The depicted examples qualitatively demonstrate the accurate segmentation performance of Cellpose and a few examples where detections were missed that are successfully identified by the classical LoG-based detection method. We found that using both approaches in combination resulted in complementary results and effectively in more complete tracks as quantitatively demonstrated in S1 Table. The average diameter of all cells and across all time points in this example is 40.79 pixels and the Cellpose diameter parameter was set to the default value of 30 pixels, to allow segmenting smaller objects like cells in meta- and anaphase as well. (TIF) [file pone.0270923.s012.tif]

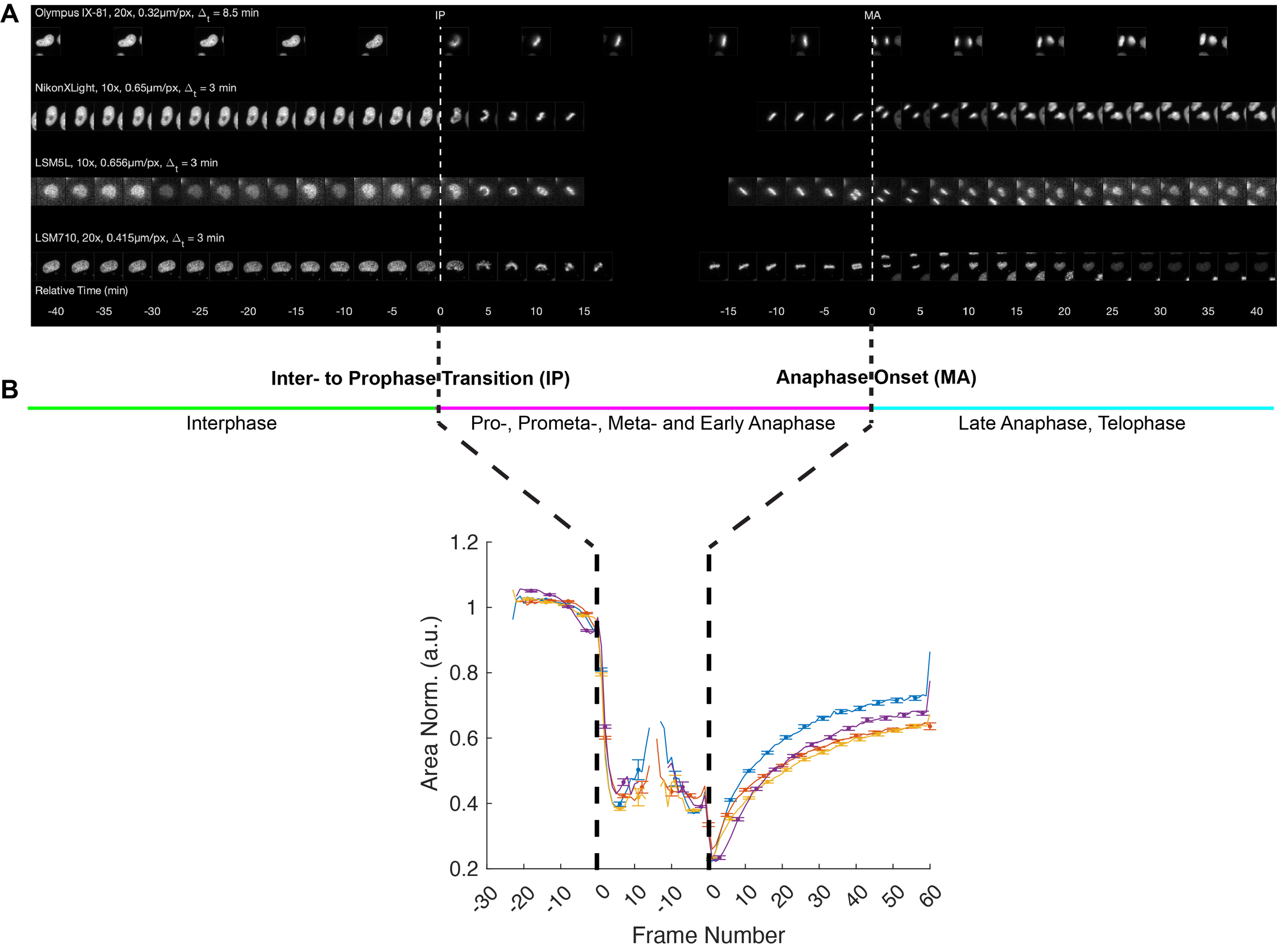

Supplement: S12 Fig — (A) Each row shows a single cell of different experiments (see legend in the figure for imaging details). Images were scaled to a consistent size, temporally aligned and contrast was adapted for better visibility. (B) In all temporal plots of the manuscript, we group the visualization into three different phases, (1) interphase, (2) pro-, prometa-, meta- and early anaphase and (3) late ana- and telophase. The dashed lines indicate the interphase to prophase transition (IP) and the metaphase to anaphase transition/anaphase onset (MA). For better visibility and to be able to visualize cells with different IP to MA duration, the frames between IP and MA are evenly distributed to the left and right. Time stamps are in minutes and display the relative timing with respect to the transition time points. (TIF) [file pone.0270923.s013.tif]

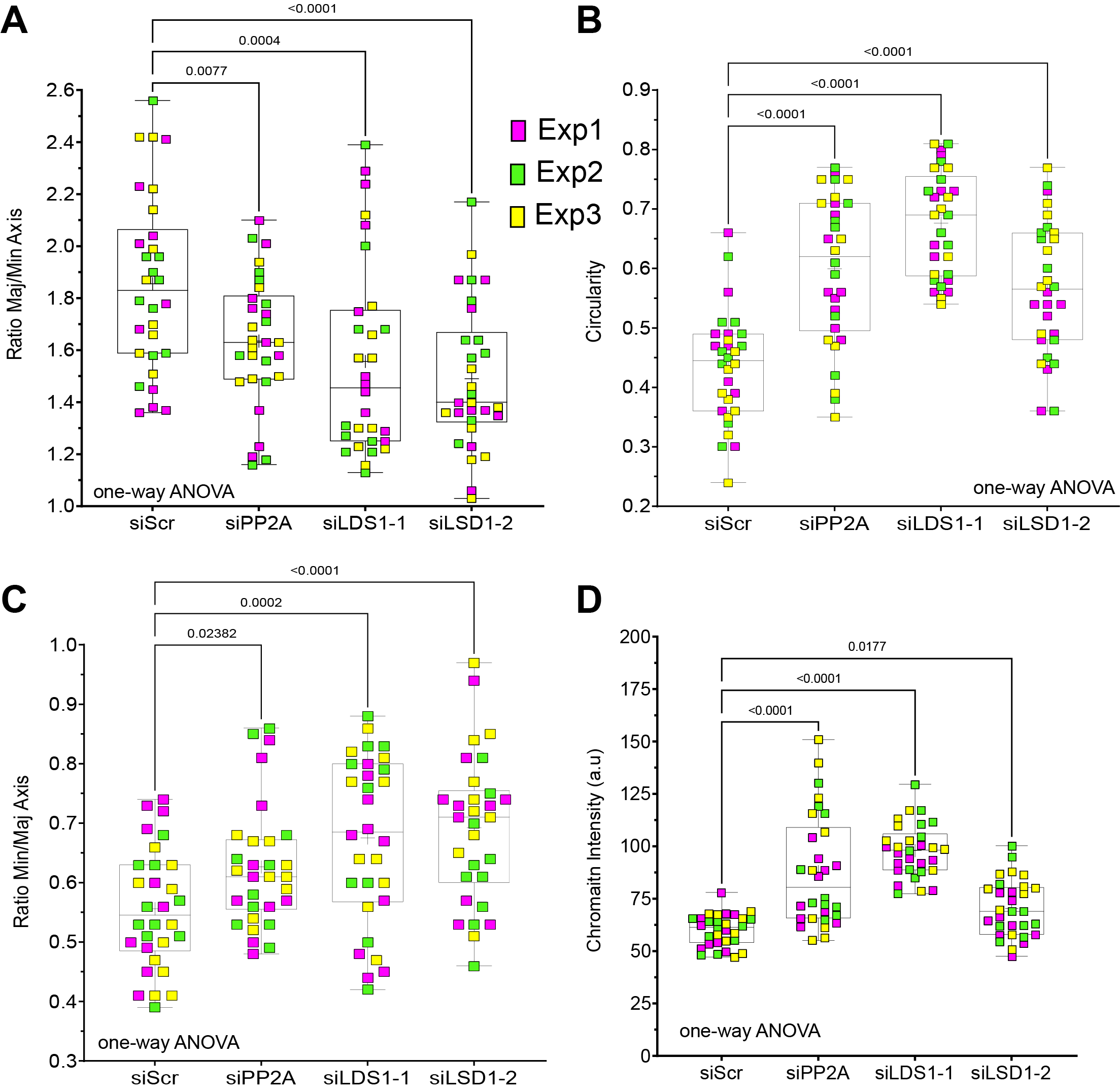

Supplement: S13 Fig — To confirm the unexpected nuclear morphology phenotypes in LSD1 and PP2A in late mitosis detected by LiveCellMiner, we manually measured the major/minor axes (A, C), circularity (B) and the mean intensity of cells (D) from the original LSD1 data set records 45h post transfection. Using Fiji [75], 10 random daughter cells 30 min after anaphase onset per condition per experiment (three independent experiments indicated in colored data points) were manually segmented with the Wand tool in legacy mode, and the values were extracted using the ROI manager. The data were organized in excel tables and plots (box and whisker min to max) and statistical analysis were done using GraphPath (D’Agostino-Pearson normality test and parametric one-way ANOVA). (TIF) [file pone.0270923.s014.tif]

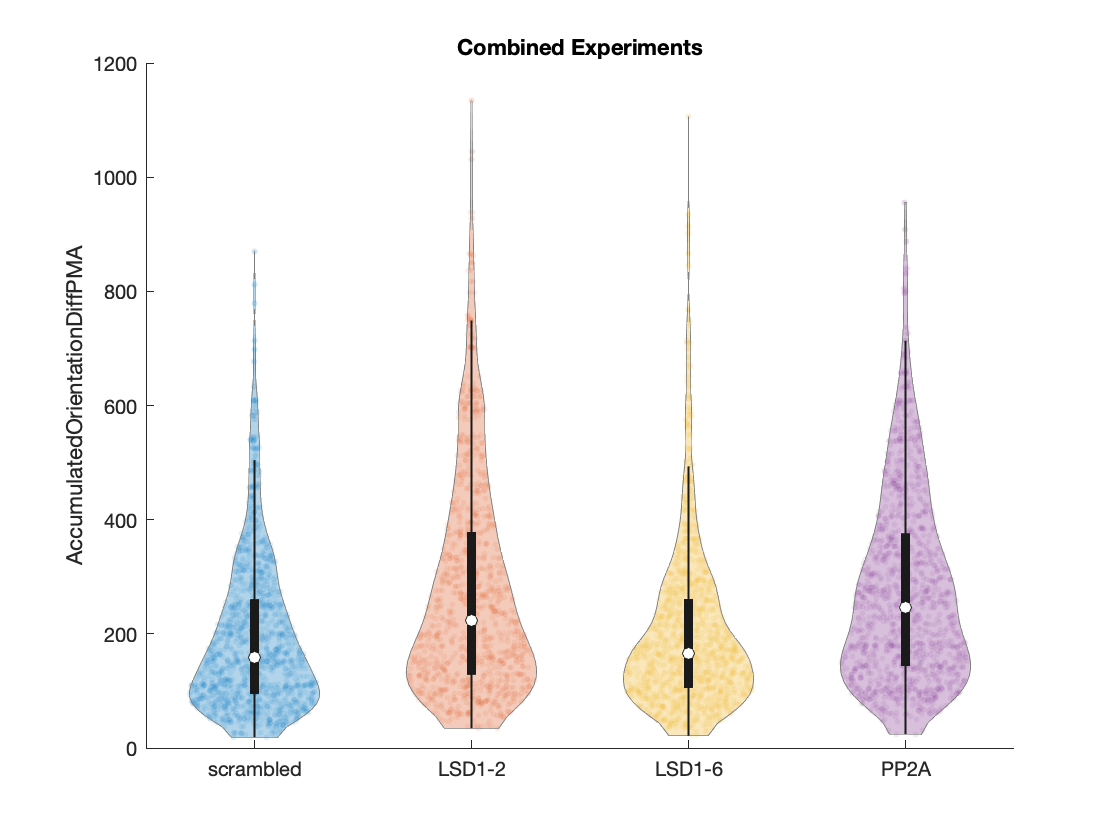

Supplement: S1 File — All existing single features and time series features are contained and accessible from an HTML-based overview file. Extract the archive to a folder of your choice and open the HTML file in the root directory using any web browser. (ZIP) [file pone.0270923.s022.zip › Plots/LSD1_FusedProjects_CARSync_AdditionalFeatures_AccumulatedOrientationDiffPMA_BoxPlots.png]

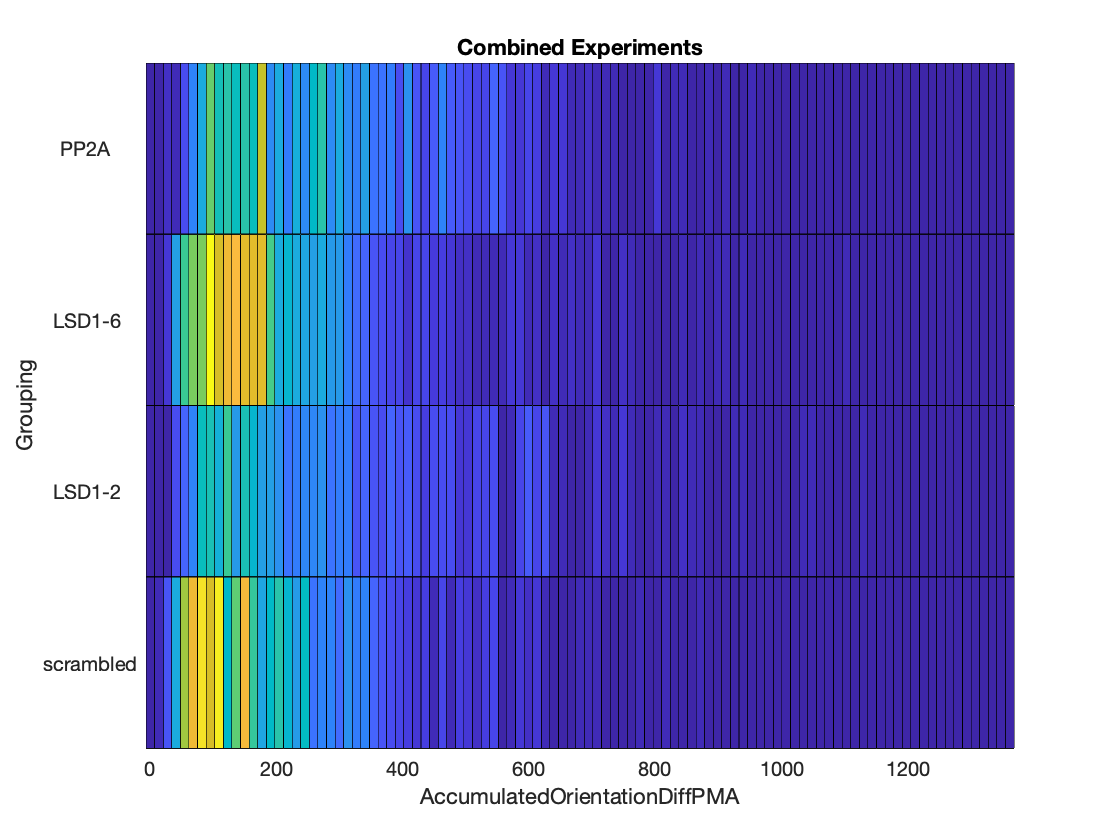

Supplement: S1 File — All existing single features and time series features are contained and accessible from an HTML-based overview file. Extract the archive to a folder of your choice and open the HTML file in the root directory using any web browser. (ZIP) [file pone.0270923.s022.zip › Plots/LSD1_FusedProjects_CARSync_AdditionalFeatures_AccumulatedOrientationDiffPMA_Histograms.png]

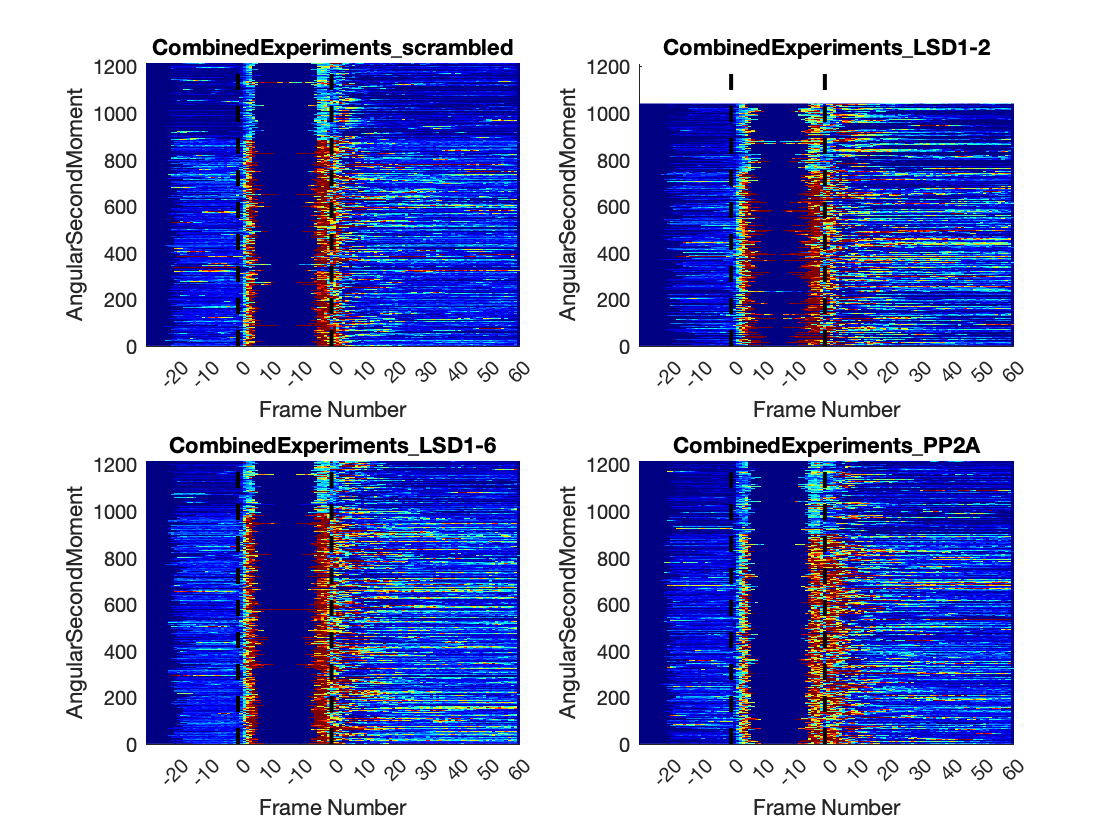

Supplement: S1 File — All existing single features and time series features are contained and accessible from an HTML-based overview file. Extract the archive to a folder of your choice and open the HTML file in the root directory using any web browser. (ZIP) [file pone.0270923.s022.zip › Plots/LSD1_FusedProjects_CARSync_AdditionalFeatures_AngularSecondMoment_HeatMaps.png]

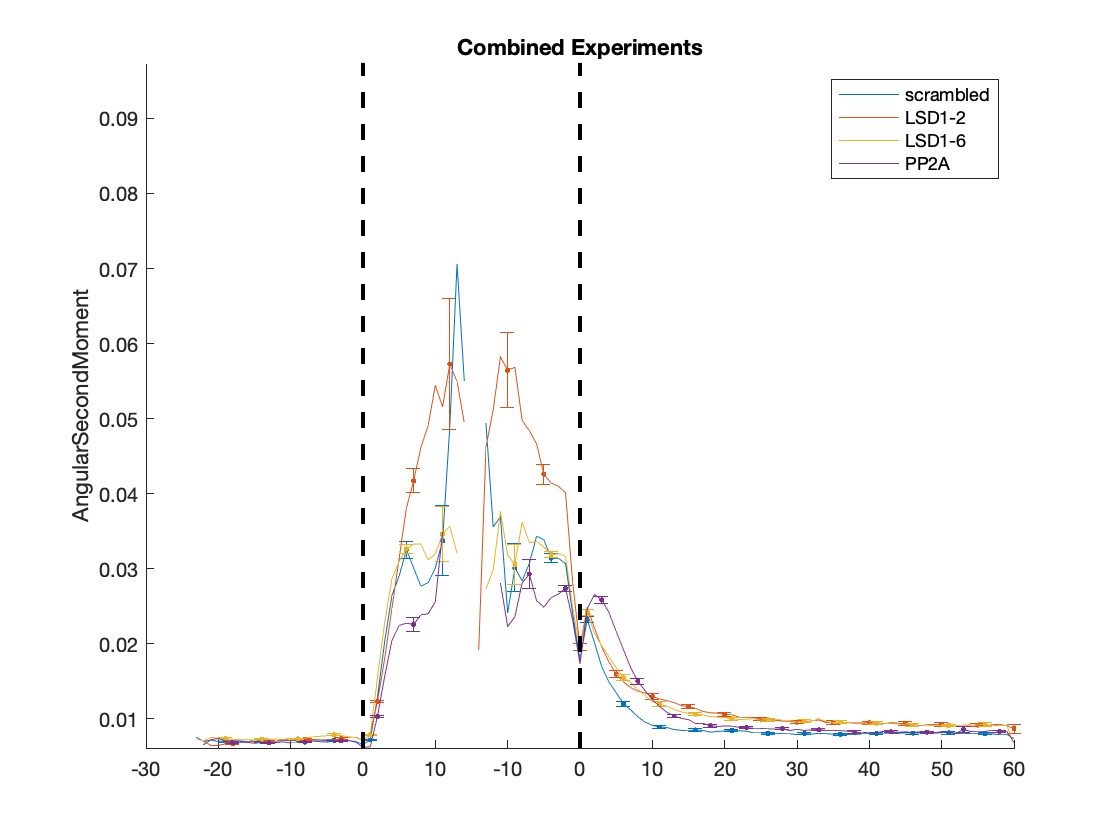

Supplement: S1 File — All existing single features and time series features are contained and accessible from an HTML-based overview file. Extract the archive to a folder of your choice and open the HTML file in the root directory using any web browser. (ZIP) [file pone.0270923.s022.zip › Plots/LSD1_FusedProjects_CARSync_AdditionalFeatures_AngularSecondMoment_LinePlots.png]

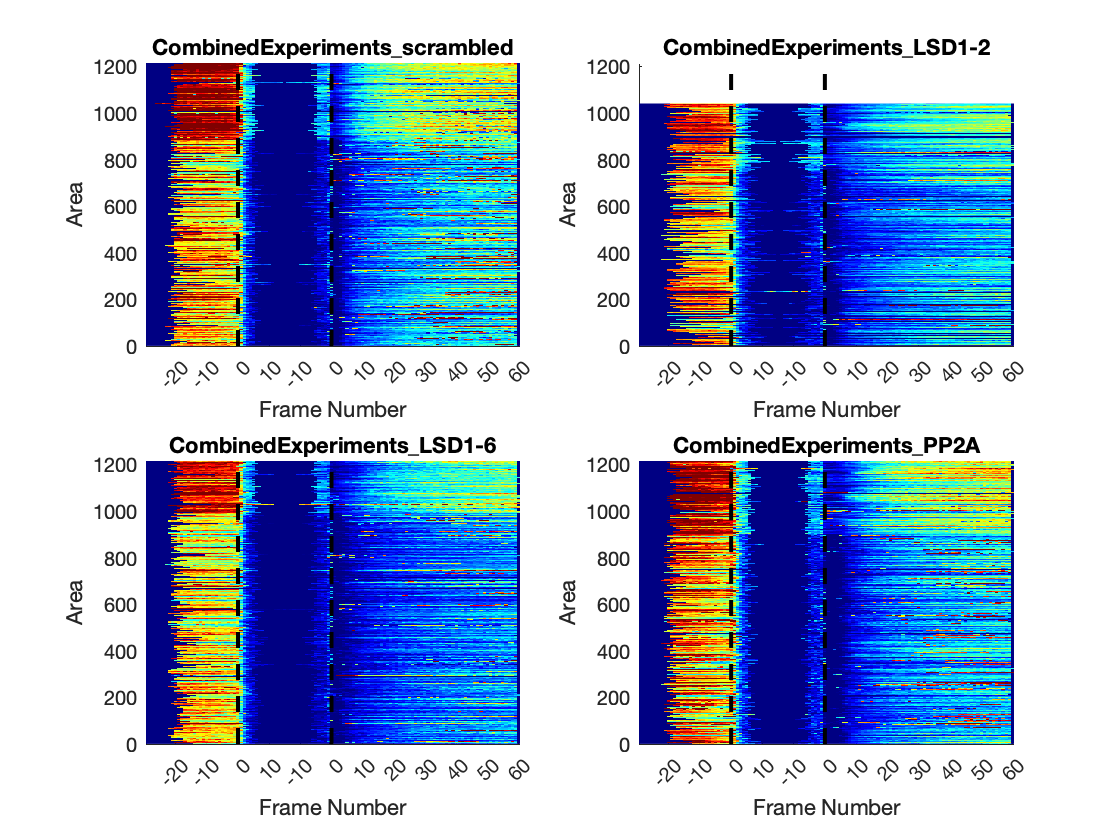

Supplement: S1 File — All existing single features and time series features are contained and accessible from an HTML-based overview file. Extract the archive to a folder of your choice and open the HTML file in the root directory using any web browser. (ZIP) [file pone.0270923.s022.zip › Plots/LSD1_FusedProjects_CARSync_AdditionalFeatures_Area_HeatMaps.png]

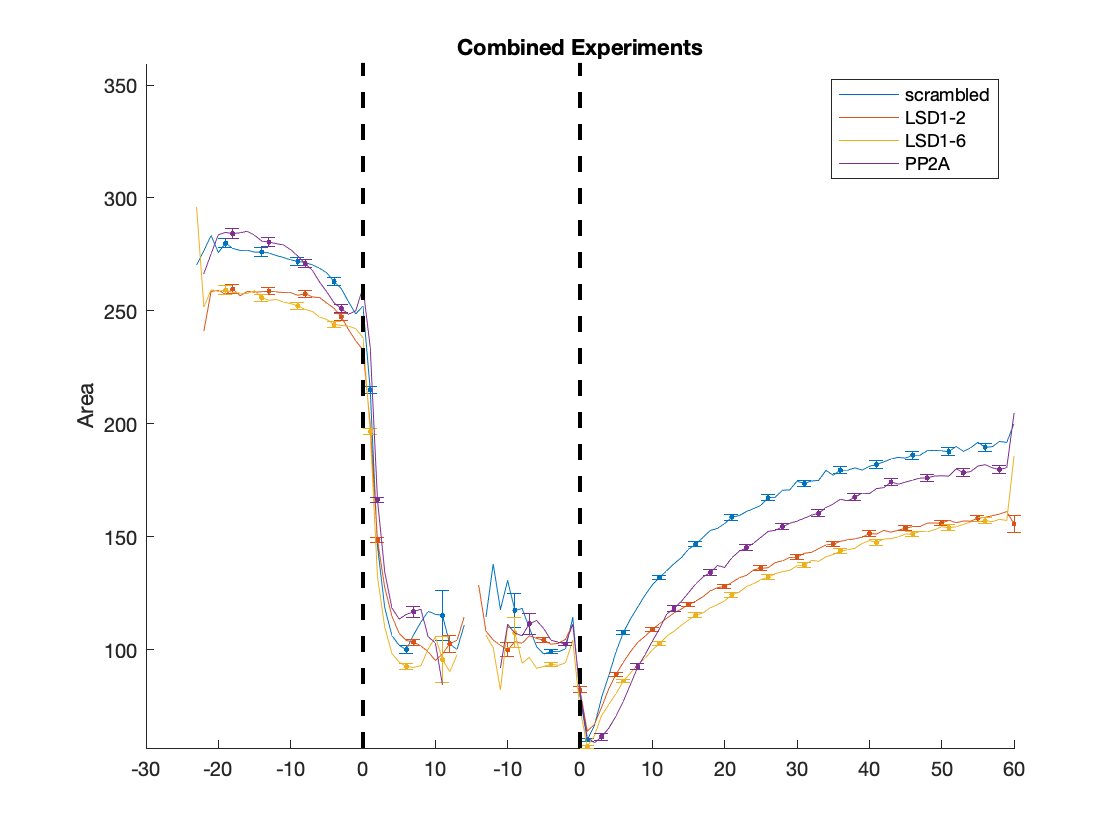

Supplement: S1 File — All existing single features and time series features are contained and accessible from an HTML-based overview file. Extract the archive to a folder of your choice and open the HTML file in the root directory using any web browser. (ZIP) [file pone.0270923.s022.zip › Plots/LSD1_FusedProjects_CARSync_AdditionalFeatures_Area_LinePlots.png]

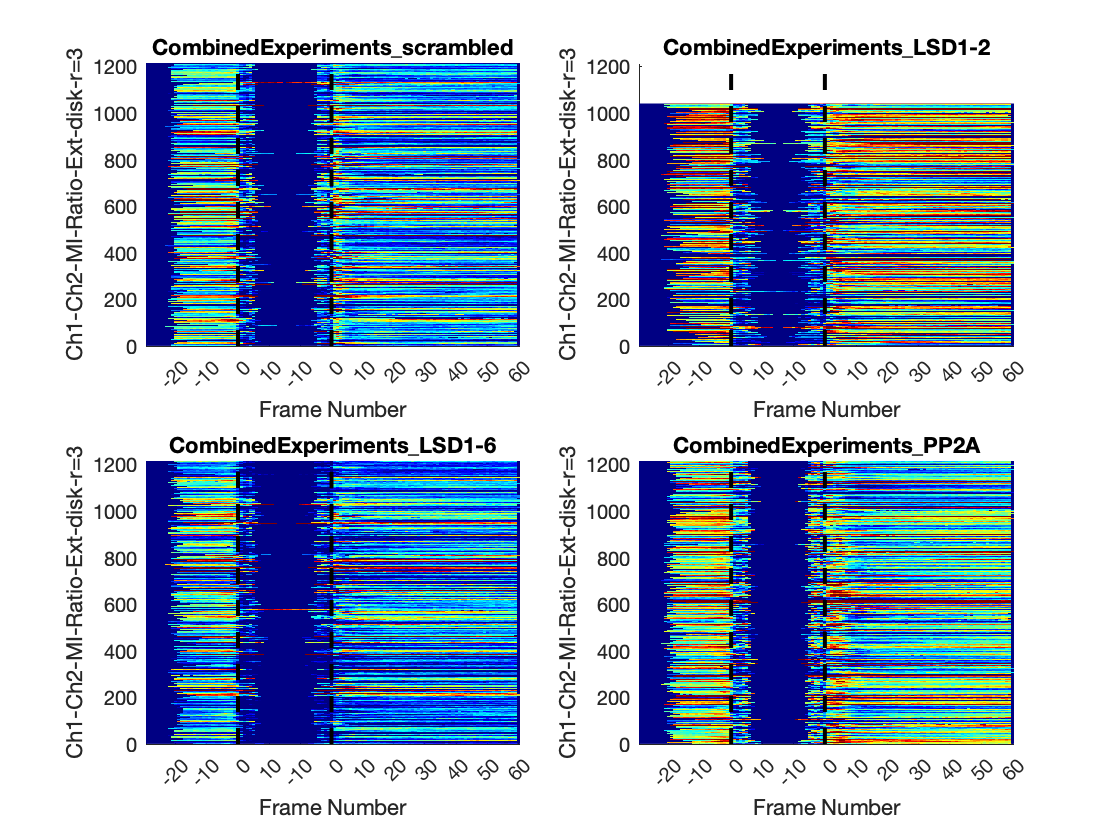

Supplement: S1 File — All existing single features and time series features are contained and accessible from an HTML-based overview file. Extract the archive to a folder of your choice and open the HTML file in the root directory using any web browser. (ZIP) [file pone.0270923.s022.zip › Plots/LSD1_FusedProjects_CARSync_AdditionalFeatures_Ch1-Ch2-MI-Ratio-Ext-disk-r=3_HeatMaps.png]

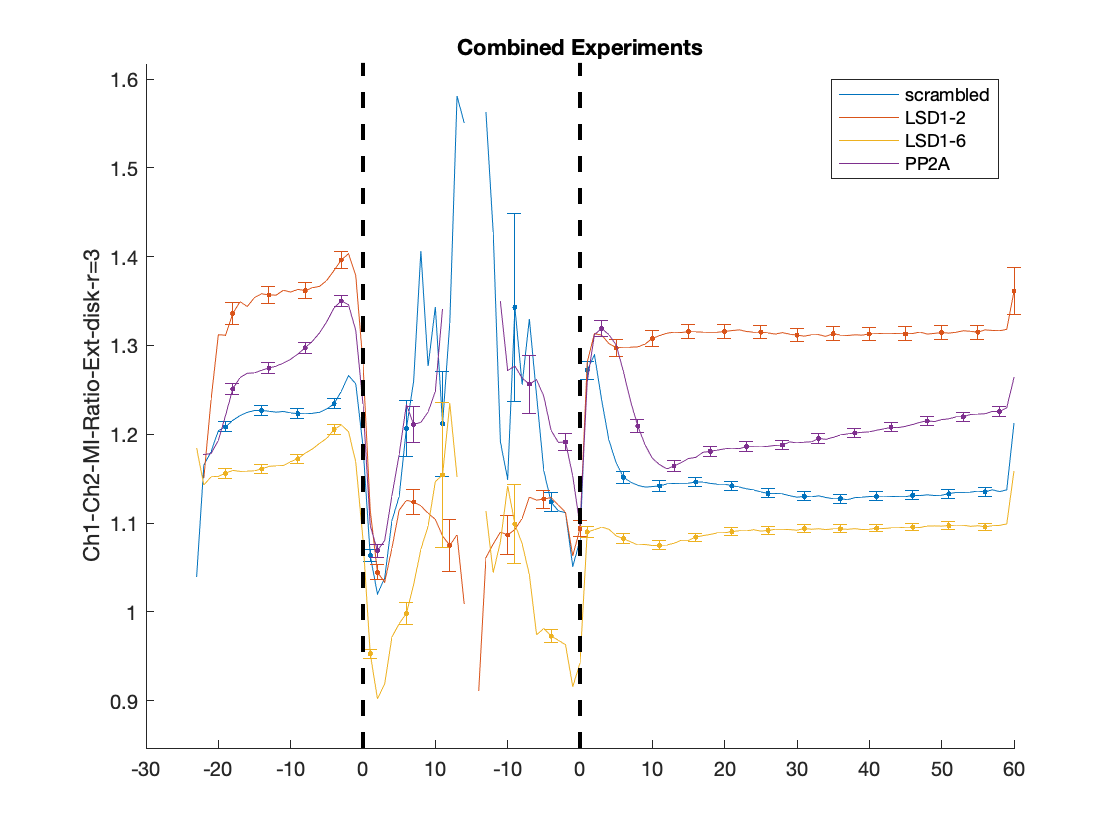

Supplement: S1 File — All existing single features and time series features are contained and accessible from an HTML-based overview file. Extract the archive to a folder of your choice and open the HTML file in the root directory using any web browser. (ZIP) [file pone.0270923.s022.zip › Plots/LSD1_FusedProjects_CARSync_AdditionalFeatures_Ch1-Ch2-MI-Ratio-Ext-disk-r=3_LinePlots.png]

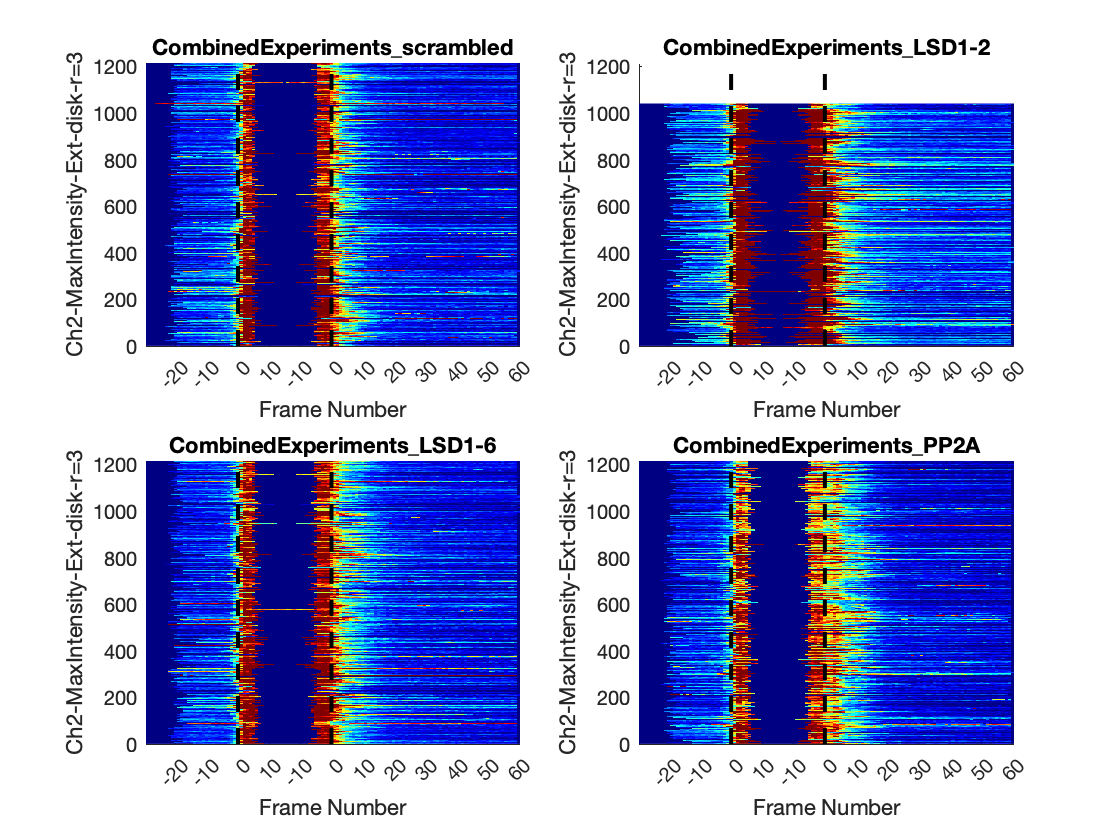

Supplement: S1 File — All existing single features and time series features are contained and accessible from an HTML-based overview file. Extract the archive to a folder of your choice and open the HTML file in the root directory using any web browser. (ZIP) [file pone.0270923.s022.zip › Plots/LSD1_FusedProjects_CARSync_AdditionalFeatures_Ch2-MaxIntensity-Ext-disk-r=3_HeatMaps.png]

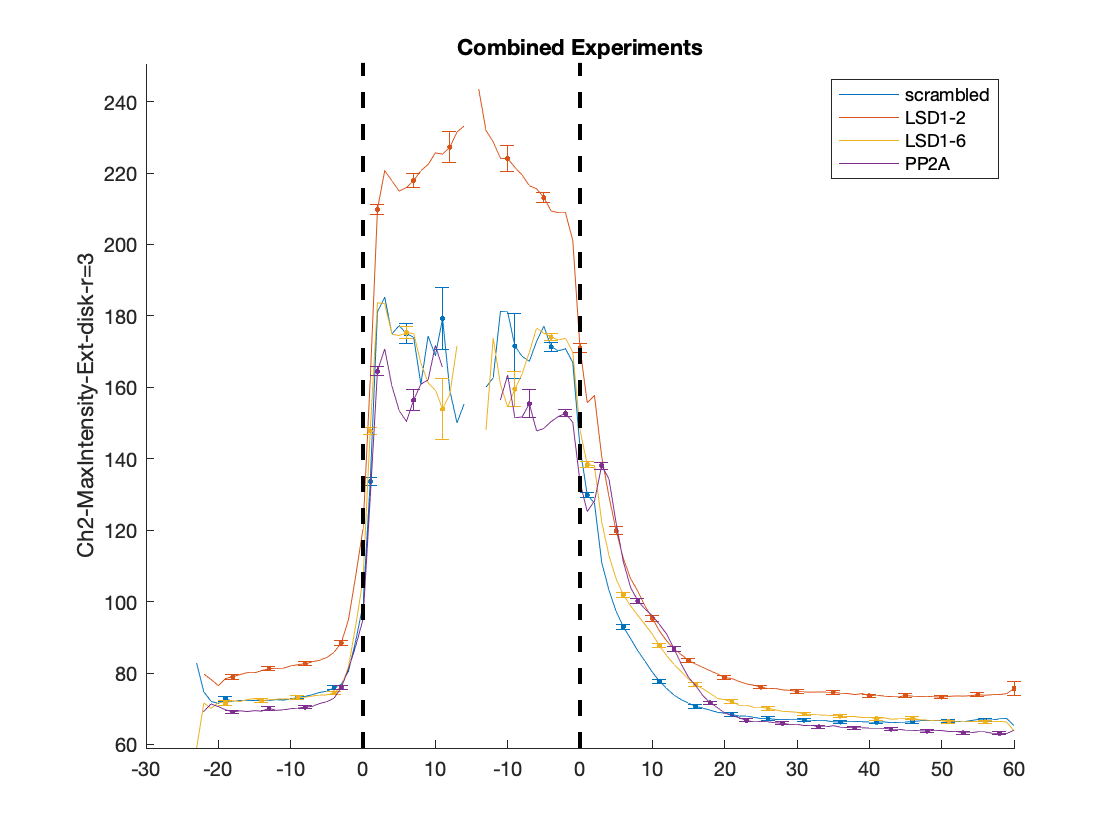

Supplement: S1 File — All existing single features and time series features are contained and accessible from an HTML-based overview file. Extract the archive to a folder of your choice and open the HTML file in the root directory using any web browser. (ZIP) [file pone.0270923.s022.zip › Plots/LSD1_FusedProjects_CARSync_AdditionalFeatures_Ch2-MaxIntensity-Ext-disk-r=3_LinePlots.png]

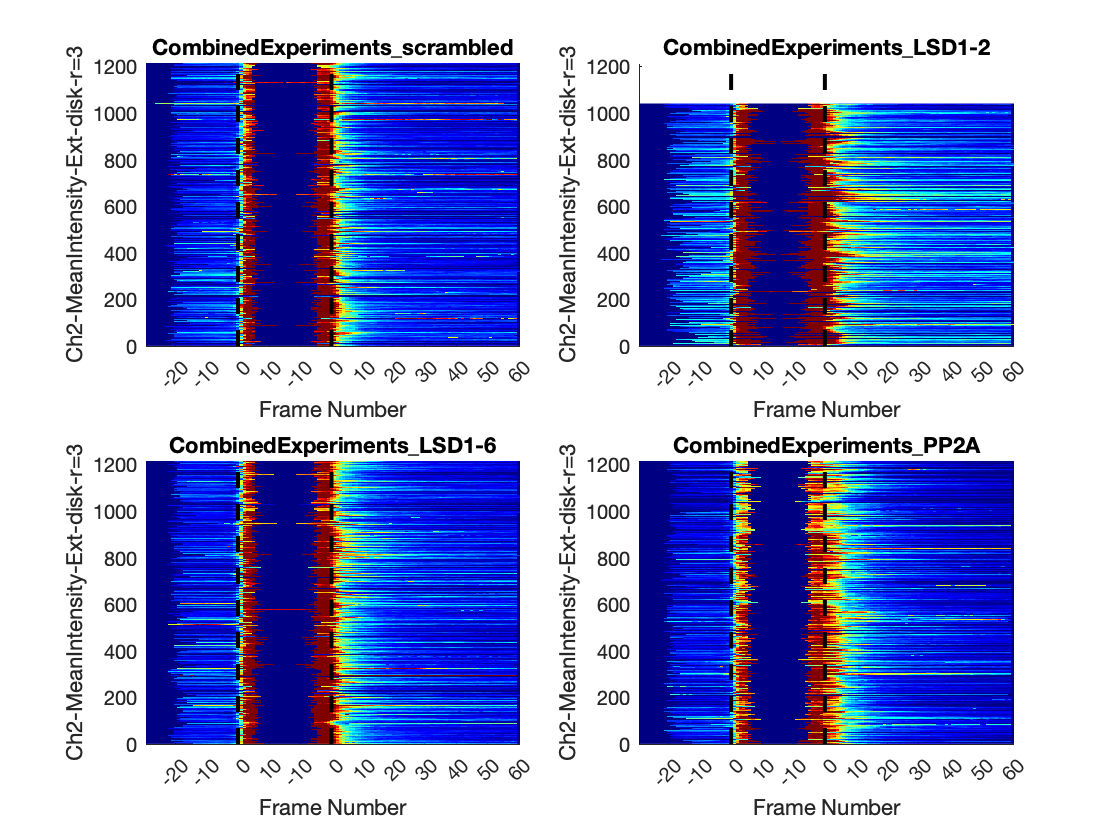

Supplement: S1 File — All existing single features and time series features are contained and accessible from an HTML-based overview file. Extract the archive to a folder of your choice and open the HTML file in the root directory using any web browser. (ZIP) [file pone.0270923.s022.zip › Plots/LSD1_FusedProjects_CARSync_AdditionalFeatures_Ch2-MeanIntensity-Ext-disk-r=3_HeatMaps.png]

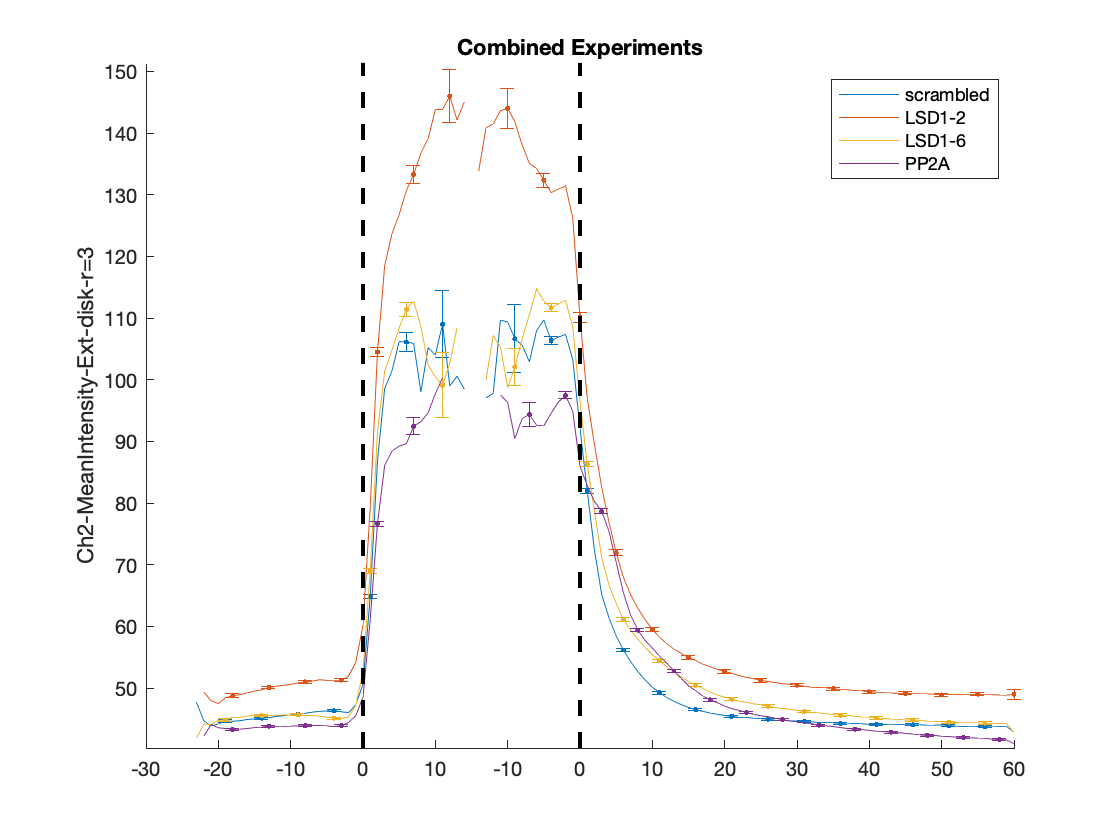

Supplement: S1 File — All existing single features and time series features are contained and accessible from an HTML-based overview file. Extract the archive to a folder of your choice and open the HTML file in the root directory using any web browser. (ZIP) [file pone.0270923.s022.zip › Plots/LSD1_FusedProjects_CARSync_AdditionalFeatures_Ch2-MeanIntensity-Ext-disk-r=3_LinePlots.png]

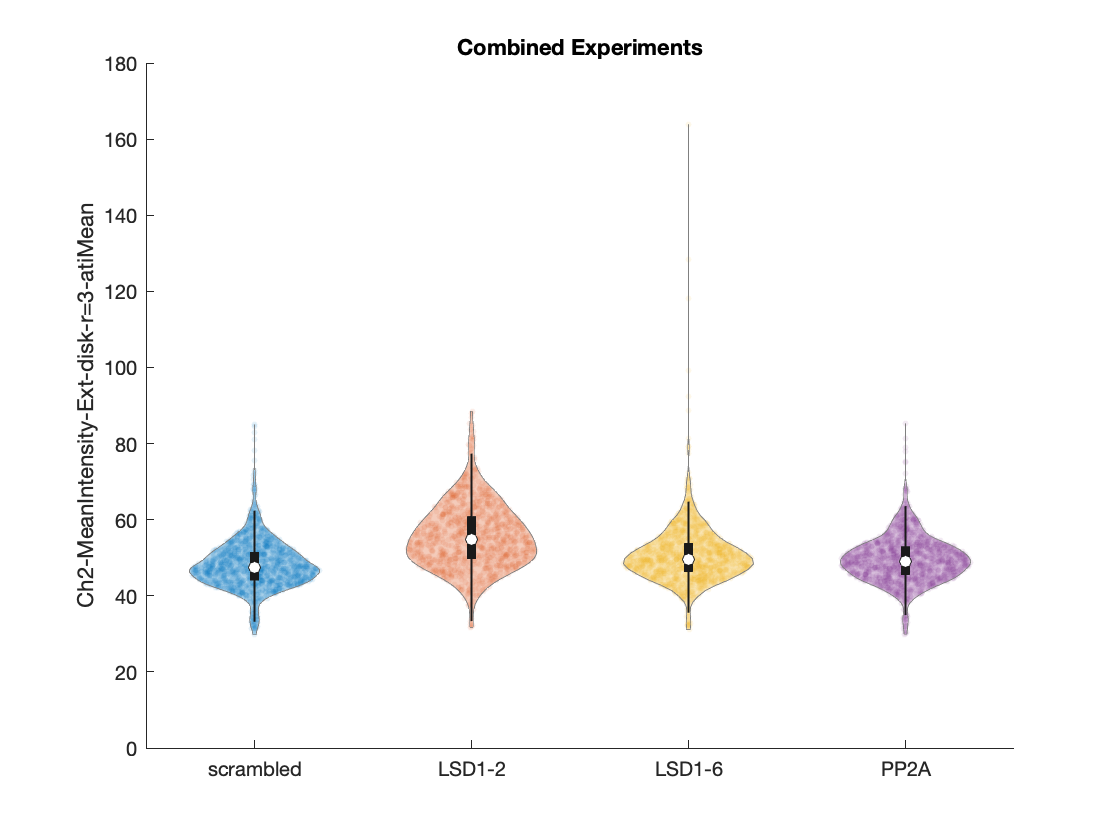

Supplement: S1 File — All existing single features and time series features are contained and accessible from an HTML-based overview file. Extract the archive to a folder of your choice and open the HTML file in the root directory using any web browser. (ZIP) [file pone.0270923.s022.zip › Plots/LSD1_FusedProjects_CARSync_AdditionalFeatures_Ch2-MeanIntensity-Ext-disk-r=3-atiMean_BoxPlots.png]

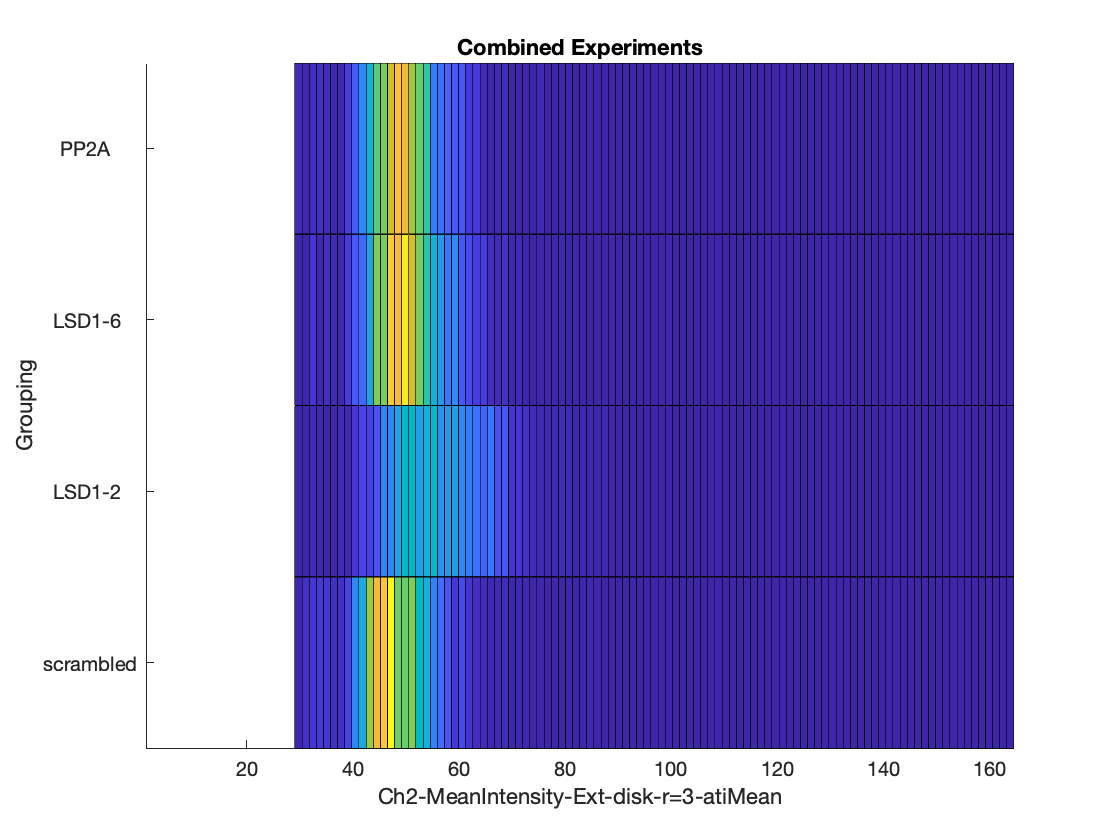

Supplement: S1 File — All existing single features and time series features are contained and accessible from an HTML-based overview file. Extract the archive to a folder of your choice and open the HTML file in the root directory using any web browser. (ZIP) [file pone.0270923.s022.zip › Plots/LSD1_FusedProjects_CARSync_AdditionalFeatures_Ch2-MeanIntensity-Ext-disk-r=3-atiMean_Histograms.png]

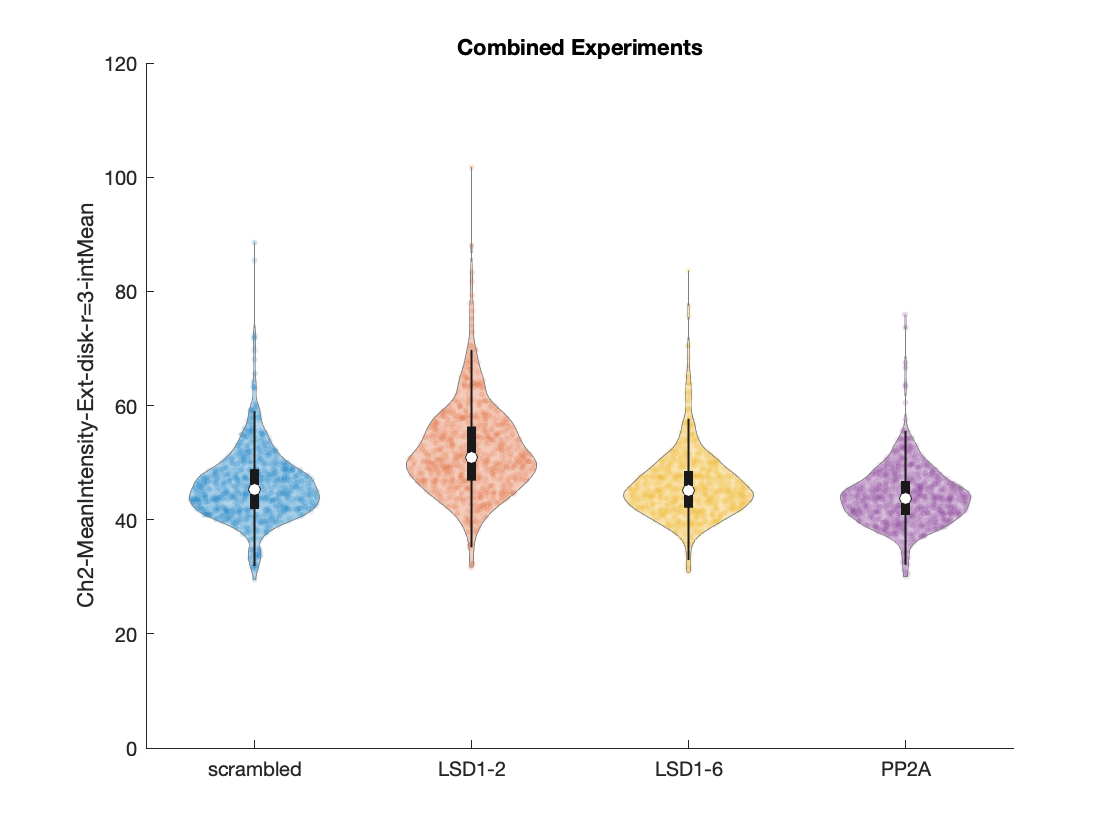

Supplement: S1 File — All existing single features and time series features are contained and accessible from an HTML-based overview file. Extract the archive to a folder of your choice and open the HTML file in the root directory using any web browser. (ZIP) [file pone.0270923.s022.zip › Plots/LSD1_FusedProjects_CARSync_AdditionalFeatures_Ch2-MeanIntensity-Ext-disk-r=3-intMean_BoxPlots.png]

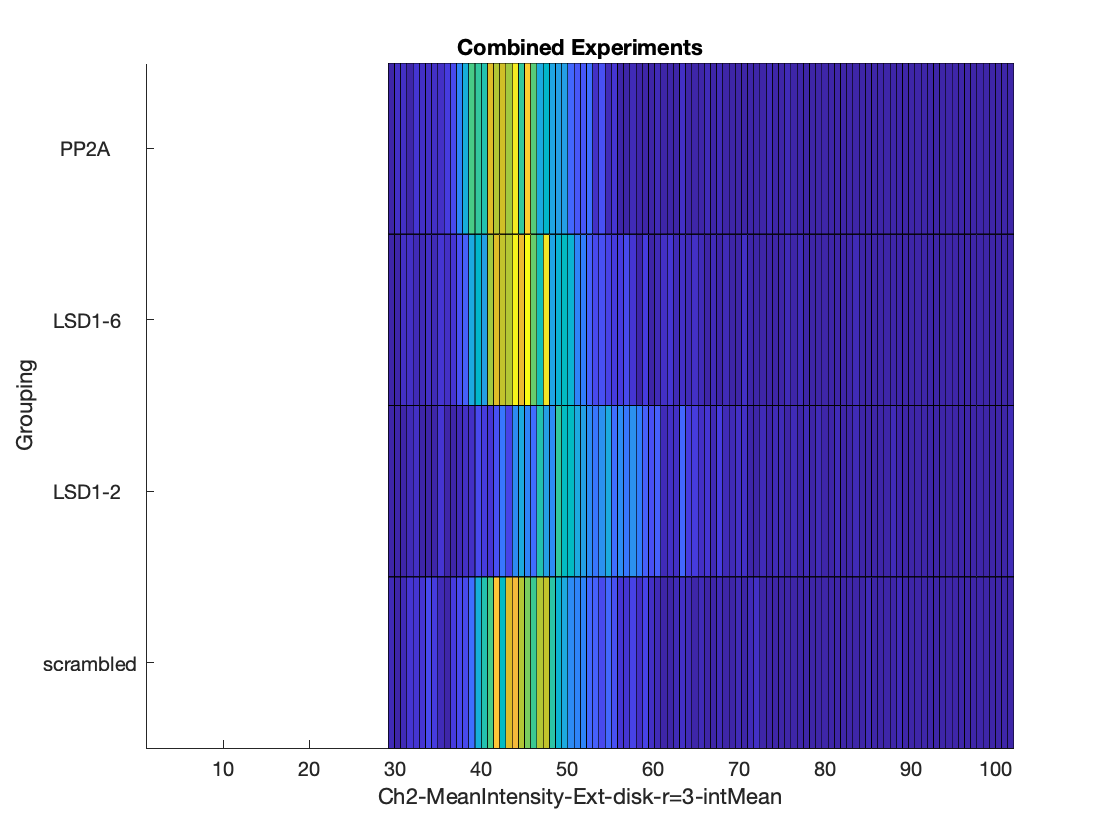

Supplement: S1 File — All existing single features and time series features are contained and accessible from an HTML-based overview file. Extract the archive to a folder of your choice and open the HTML file in the root directory using any web browser. (ZIP) [file pone.0270923.s022.zip › Plots/LSD1_FusedProjects_CARSync_AdditionalFeatures_Ch2-MeanIntensity-Ext-disk-r=3-intMean_Histograms.png]

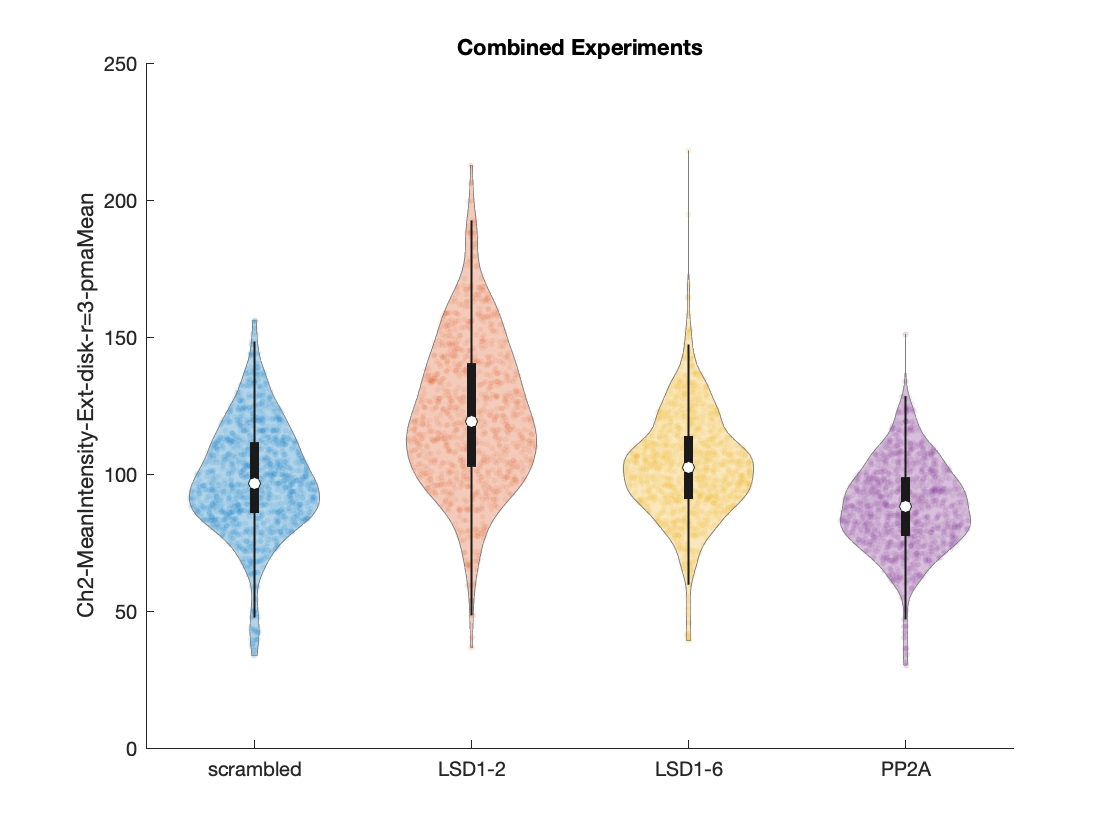

Supplement: S1 File — All existing single features and time series features are contained and accessible from an HTML-based overview file. Extract the archive to a folder of your choice and open the HTML file in the root directory using any web browser. (ZIP) [file pone.0270923.s022.zip › Plots/LSD1_FusedProjects_CARSync_AdditionalFeatures_Ch2-MeanIntensity-Ext-disk-r=3-pmaMean_BoxPlots.png]

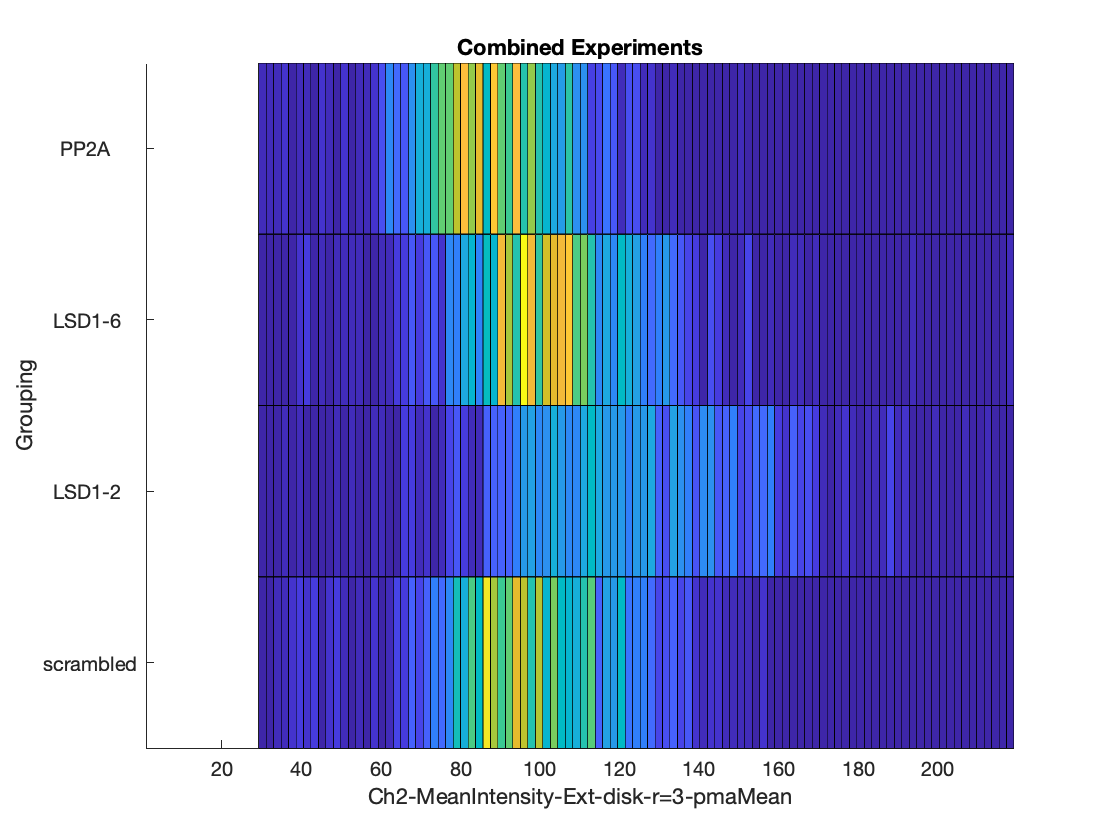

Supplement: S1 File — All existing single features and time series features are contained and accessible from an HTML-based overview file. Extract the archive to a folder of your choice and open the HTML file in the root directory using any web browser. (ZIP) [file pone.0270923.s022.zip › Plots/LSD1_FusedProjects_CARSync_AdditionalFeatures_Ch2-MeanIntensity-Ext-disk-r=3-pmaMean_Histograms.png]

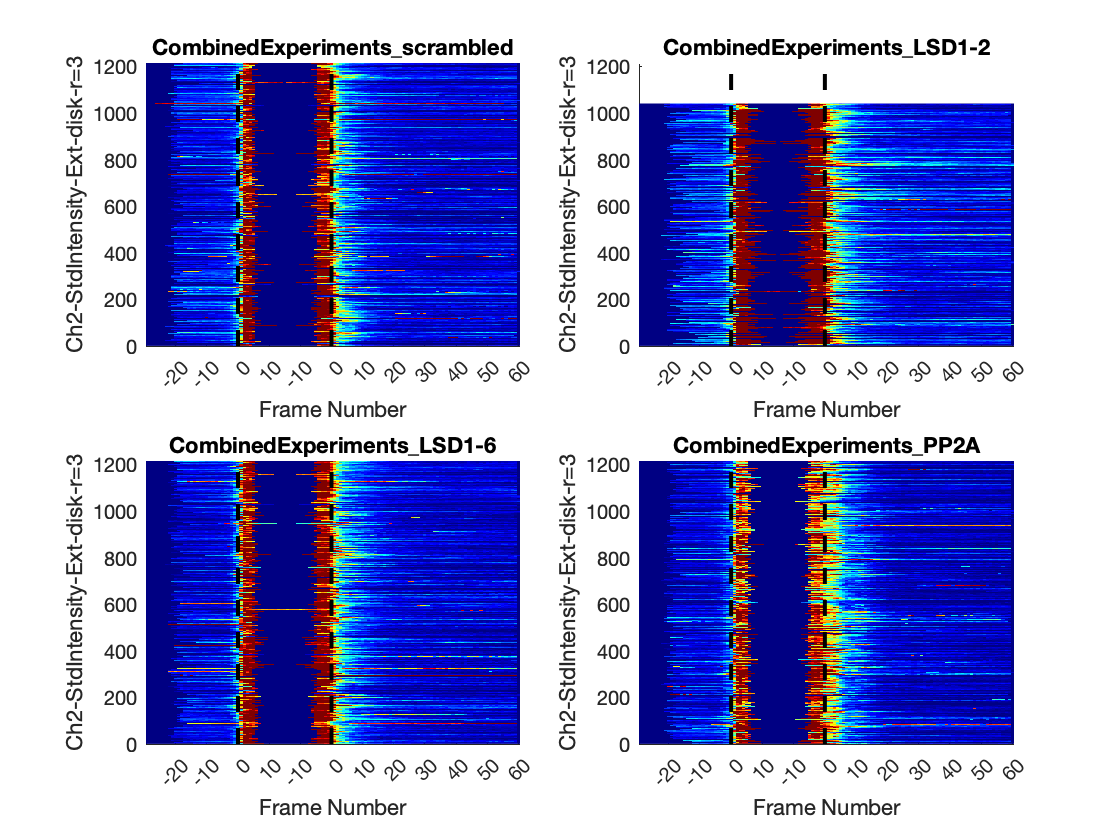

Supplement: S1 File — All existing single features and time series features are contained and accessible from an HTML-based overview file. Extract the archive to a folder of your choice and open the HTML file in the root directory using any web browser. (ZIP) [file pone.0270923.s022.zip › Plots/LSD1_FusedProjects_CARSync_AdditionalFeatures_Ch2-StdIntensity-Ext-disk-r=3_HeatMaps.png]

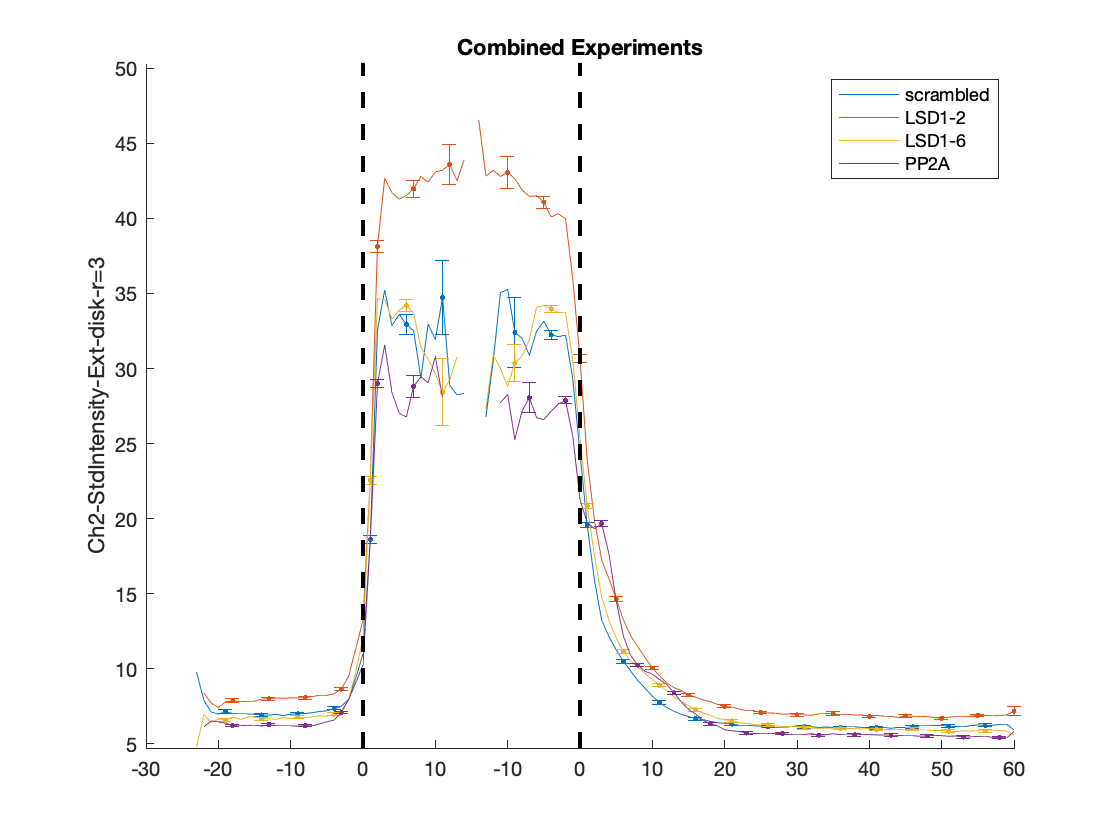

Supplement: S1 File — All existing single features and time series features are contained and accessible from an HTML-based overview file. Extract the archive to a folder of your choice and open the HTML file in the root directory using any web browser. (ZIP) [file pone.0270923.s022.zip › Plots/LSD1_FusedProjects_CARSync_AdditionalFeatures_Ch2-StdIntensity-Ext-disk-r=3_LinePlots.png]

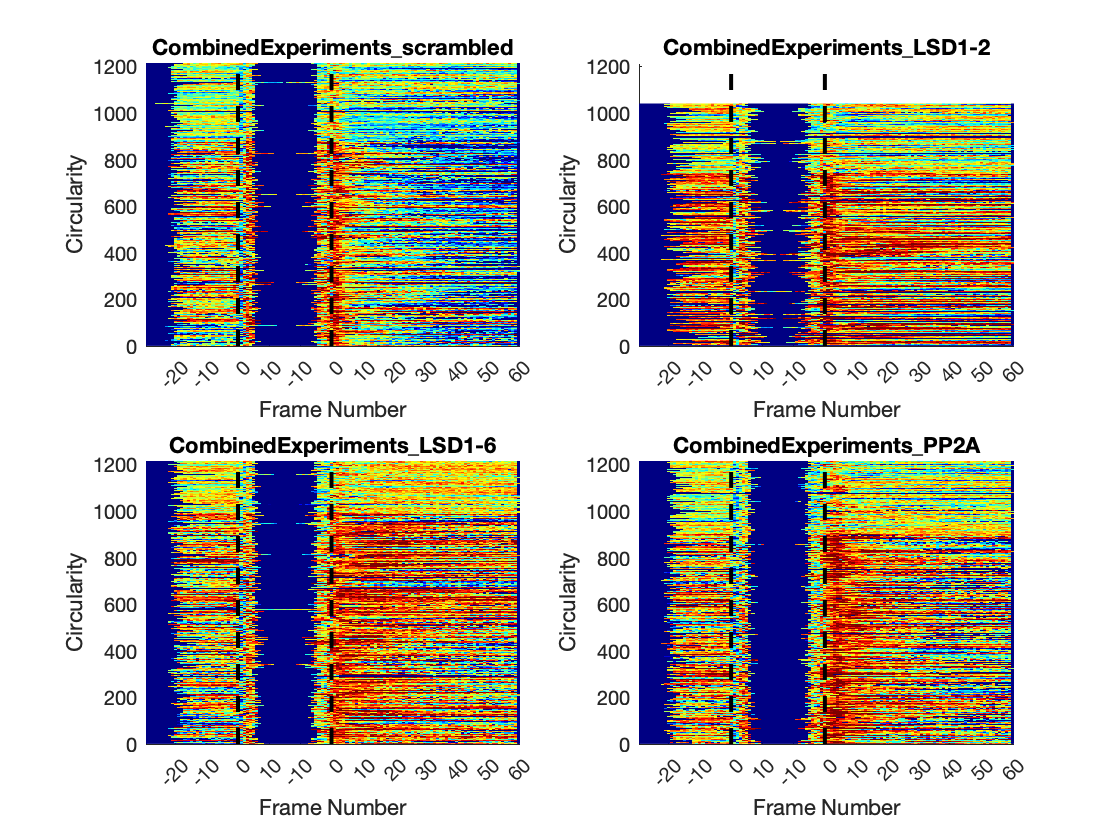

Supplement: S1 File — All existing single features and time series features are contained and accessible from an HTML-based overview file. Extract the archive to a folder of your choice and open the HTML file in the root directory using any web browser. (ZIP) [file pone.0270923.s022.zip › Plots/LSD1_FusedProjects_CARSync_AdditionalFeatures_Circularity_HeatMaps.png]

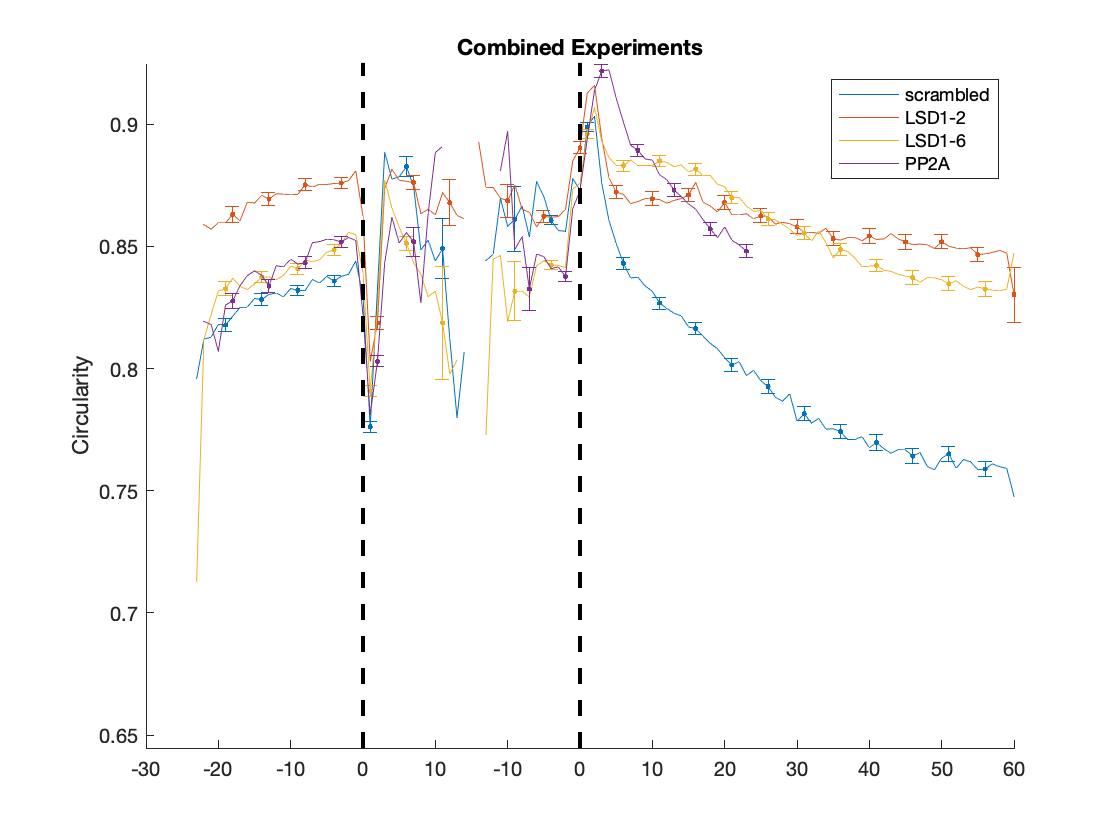

Supplement: S1 File — All existing single features and time series features are contained and accessible from an HTML-based overview file. Extract the archive to a folder of your choice and open the HTML file in the root directory using any web browser. (ZIP) [file pone.0270923.s022.zip › Plots/LSD1_FusedProjects_CARSync_AdditionalFeatures_Circularity_LinePlots.png]

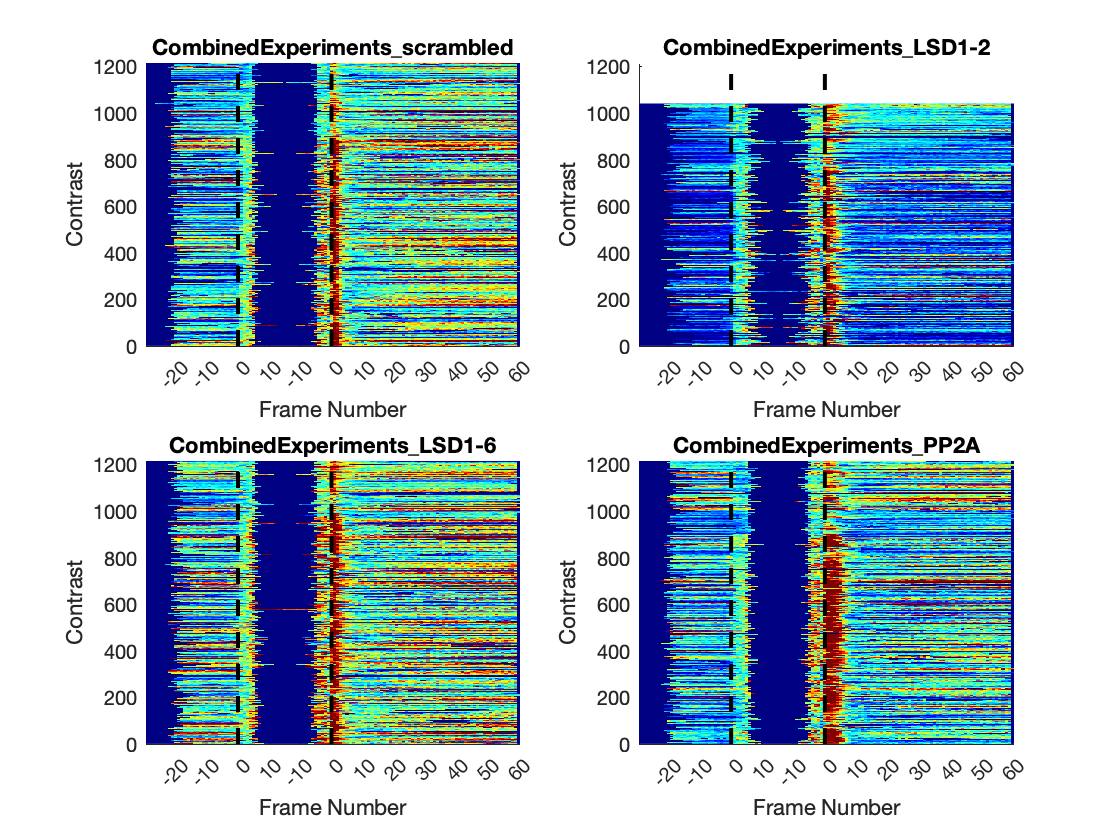

Supplement: S1 File — All existing single features and time series features are contained and accessible from an HTML-based overview file. Extract the archive to a folder of your choice and open the HTML file in the root directory using any web browser. (ZIP) [file pone.0270923.s022.zip › Plots/LSD1_FusedProjects_CARSync_AdditionalFeatures_Contrast_HeatMaps.png]

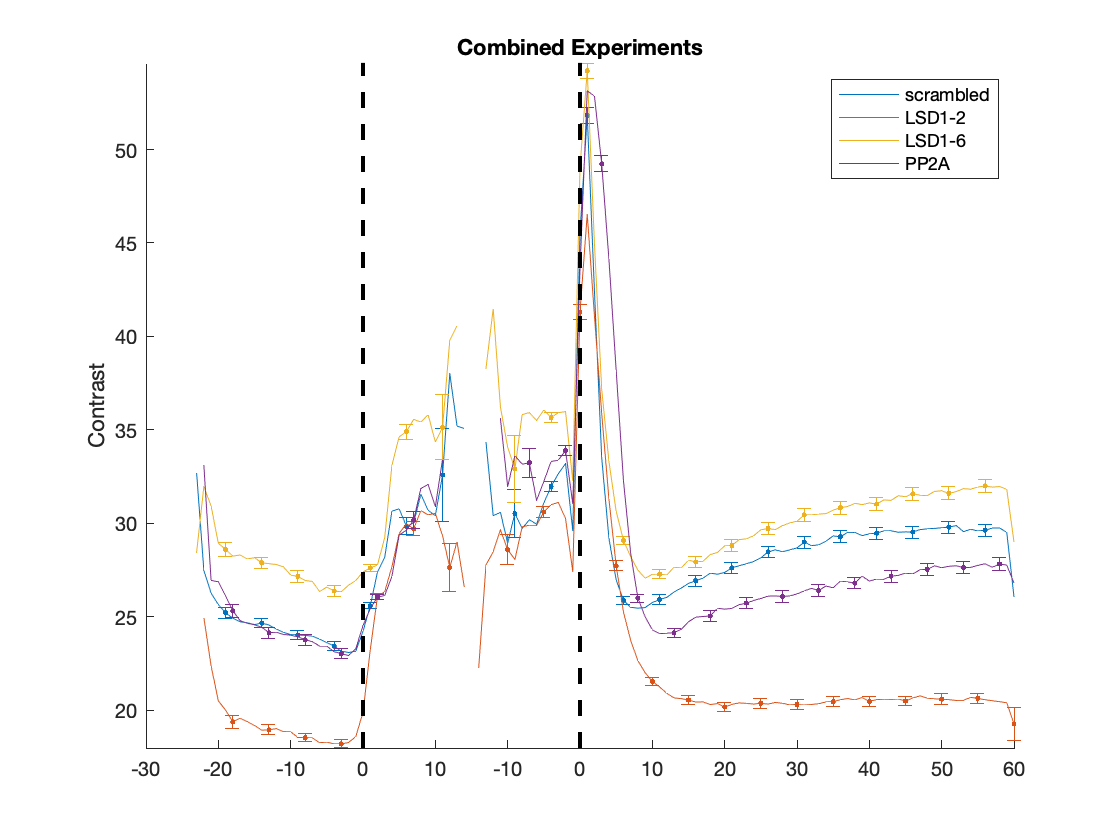

Supplement: S1 File — All existing single features and time series features are contained and accessible from an HTML-based overview file. Extract the archive to a folder of your choice and open the HTML file in the root directory using any web browser. (ZIP) [file pone.0270923.s022.zip › Plots/LSD1_FusedProjects_CARSync_AdditionalFeatures_Contrast_LinePlots.png]

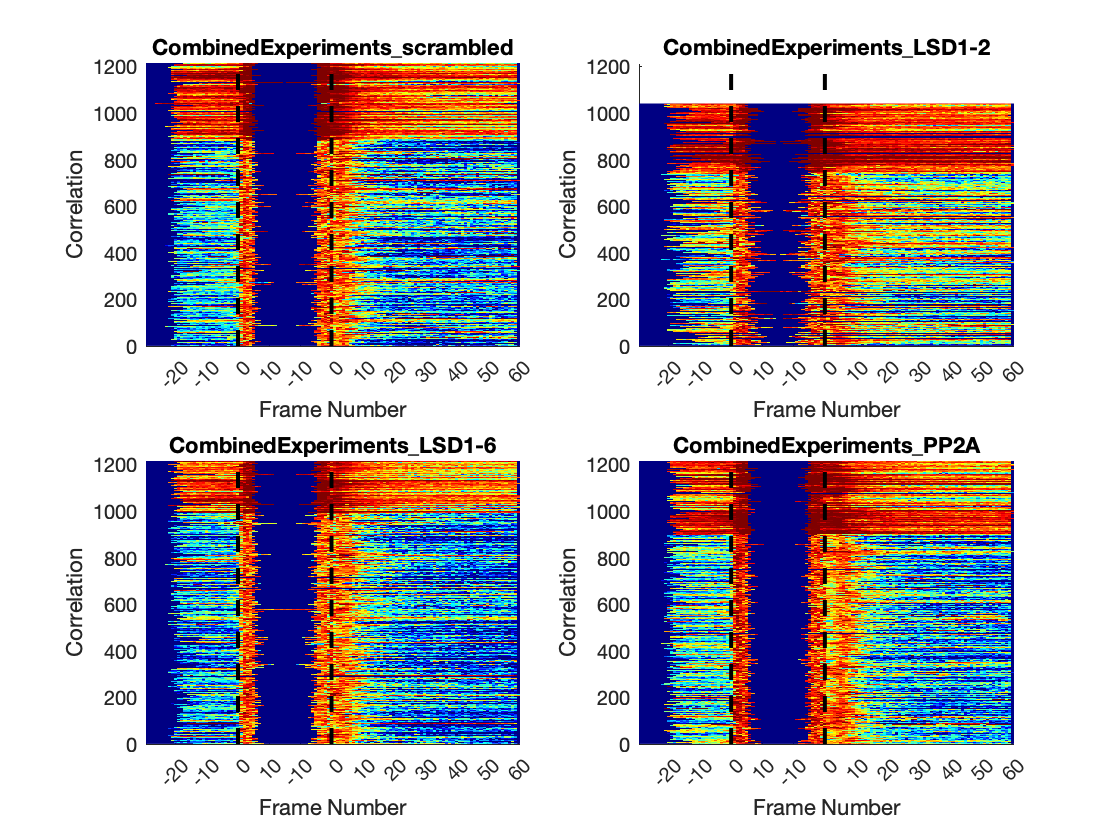

Supplement: S1 File — All existing single features and time series features are contained and accessible from an HTML-based overview file. Extract the archive to a folder of your choice and open the HTML file in the root directory using any web browser. (ZIP) [file pone.0270923.s022.zip › Plots/LSD1_FusedProjects_CARSync_AdditionalFeatures_Correlation_HeatMaps.png]

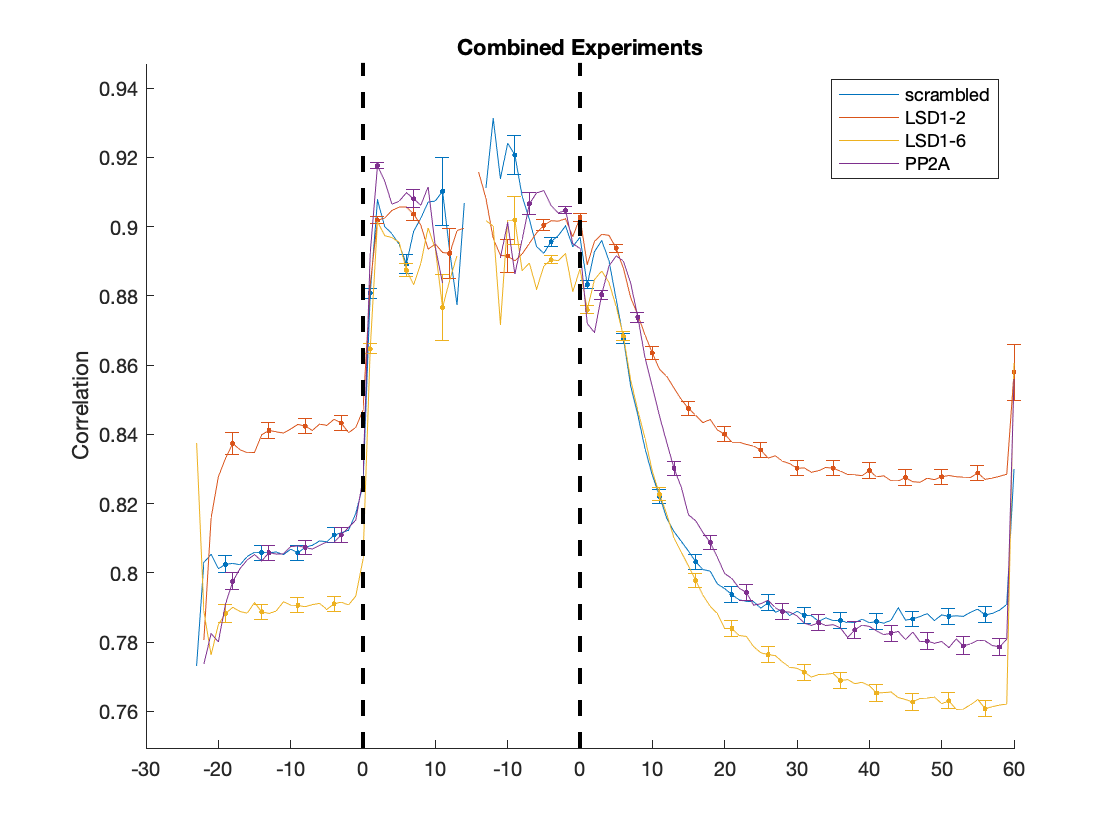

Supplement: S1 File — All existing single features and time series features are contained and accessible from an HTML-based overview file. Extract the archive to a folder of your choice and open the HTML file in the root directory using any web browser. (ZIP) [file pone.0270923.s022.zip › Plots/LSD1_FusedProjects_CARSync_AdditionalFeatures_Correlation_LinePlots.png]

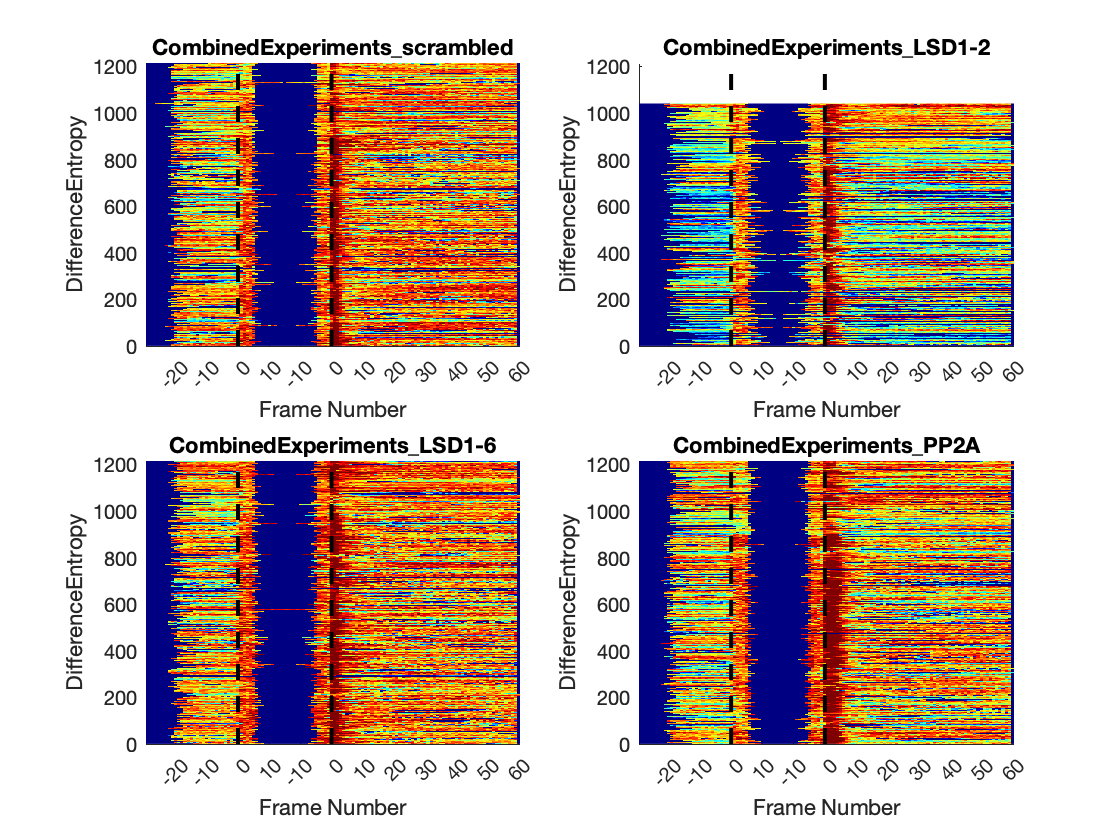

Supplement: S1 File — All existing single features and time series features are contained and accessible from an HTML-based overview file. Extract the archive to a folder of your choice and open the HTML file in the root directory using any web browser. (ZIP) [file pone.0270923.s022.zip › Plots/LSD1_FusedProjects_CARSync_AdditionalFeatures_DifferenceEntropy_HeatMaps.png]

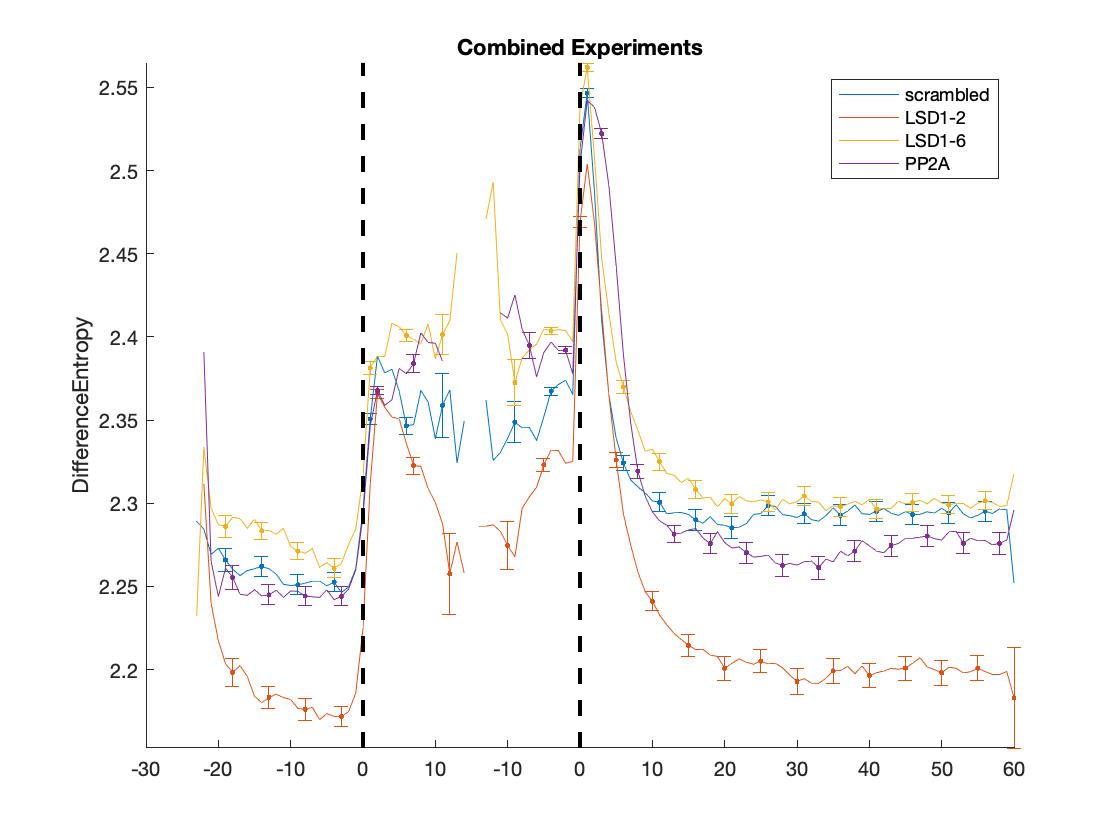

Supplement: S1 File — All existing single features and time series features are contained and accessible from an HTML-based overview file. Extract the archive to a folder of your choice and open the HTML file in the root directory using any web browser. (ZIP) [file pone.0270923.s022.zip › Plots/LSD1_FusedProjects_CARSync_AdditionalFeatures_DifferenceEntropy_LinePlots.png]

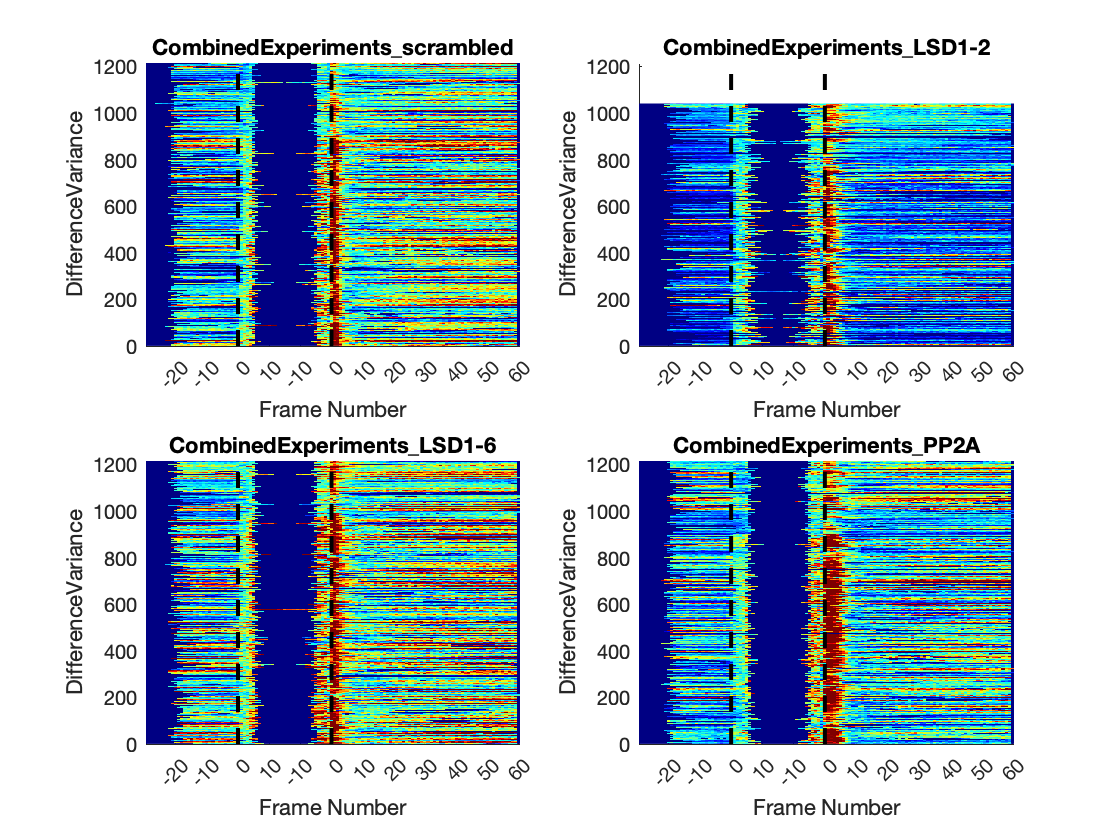

Supplement: S1 File — All existing single features and time series features are contained and accessible from an HTML-based overview file. Extract the archive to a folder of your choice and open the HTML file in the root directory using any web browser. (ZIP) [file pone.0270923.s022.zip › Plots/LSD1_FusedProjects_CARSync_AdditionalFeatures_DifferenceVariance_HeatMaps.png]

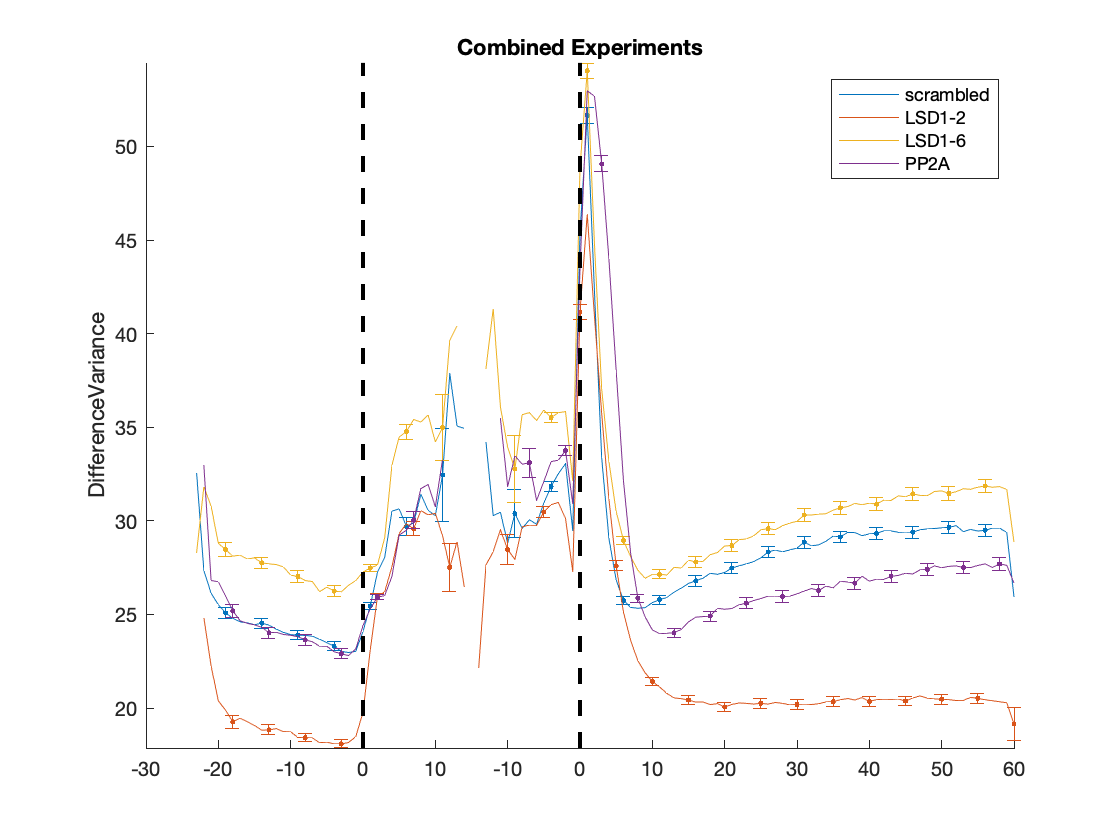

Supplement: S1 File — All existing single features and time series features are contained and accessible from an HTML-based overview file. Extract the archive to a folder of your choice and open the HTML file in the root directory using any web browser. (ZIP) [file pone.0270923.s022.zip › Plots/LSD1_FusedProjects_CARSync_AdditionalFeatures_DifferenceVariance_LinePlots.png]

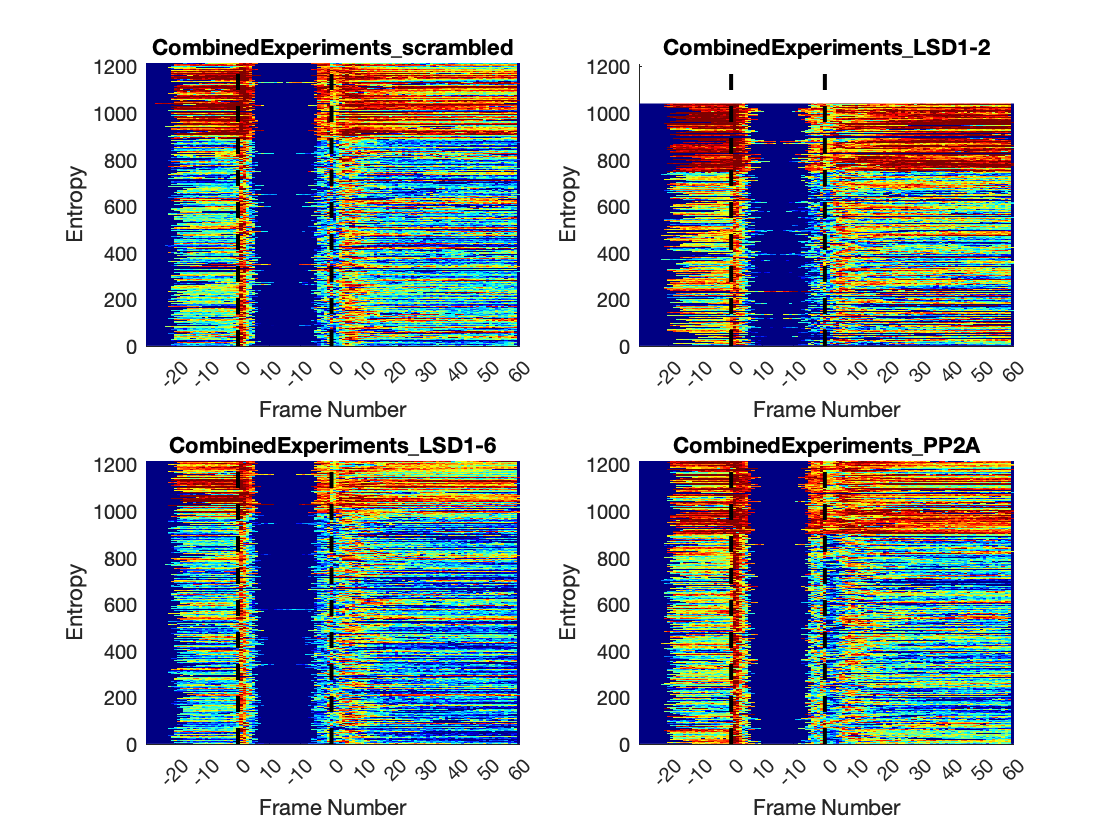

Supplement: S1 File — All existing single features and time series features are contained and accessible from an HTML-based overview file. Extract the archive to a folder of your choice and open the HTML file in the root directory using any web browser. (ZIP) [file pone.0270923.s022.zip › Plots/LSD1_FusedProjects_CARSync_AdditionalFeatures_Entropy_HeatMaps.png]

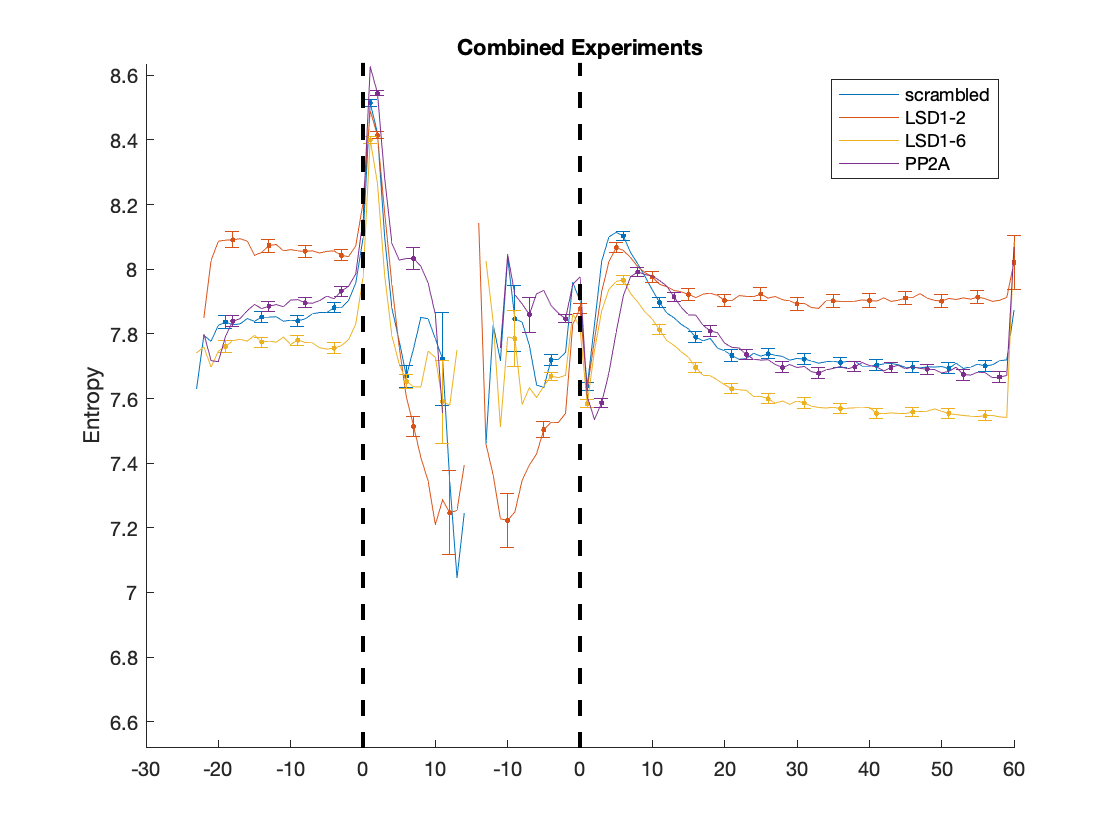

Supplement: S1 File — All existing single features and time series features are contained and accessible from an HTML-based overview file. Extract the archive to a folder of your choice and open the HTML file in the root directory using any web browser. (ZIP) [file pone.0270923.s022.zip › Plots/LSD1_FusedProjects_CARSync_AdditionalFeatures_Entropy_LinePlots.png]

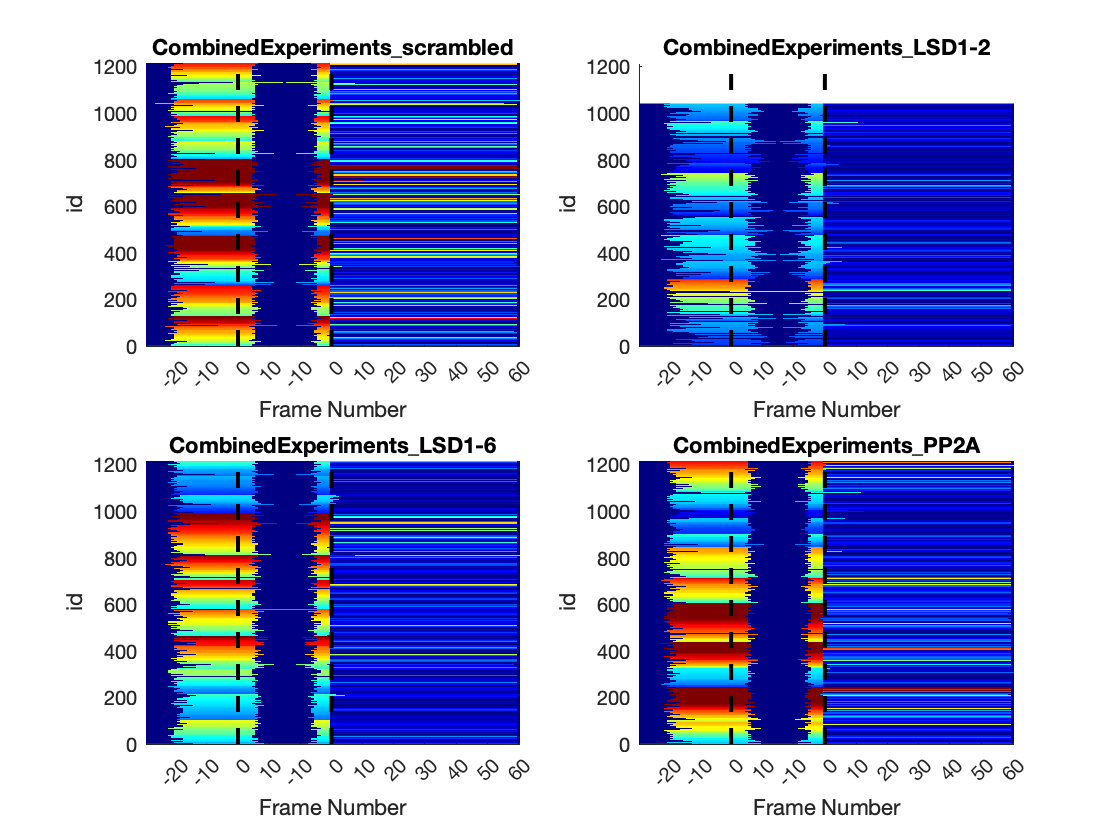

Supplement: S1 File — All existing single features and time series features are contained and accessible from an HTML-based overview file. Extract the archive to a folder of your choice and open the HTML file in the root directory using any web browser. (ZIP) [file pone.0270923.s022.zip › Plots/LSD1_FusedProjects_CARSync_AdditionalFeatures_id_HeatMaps.png]

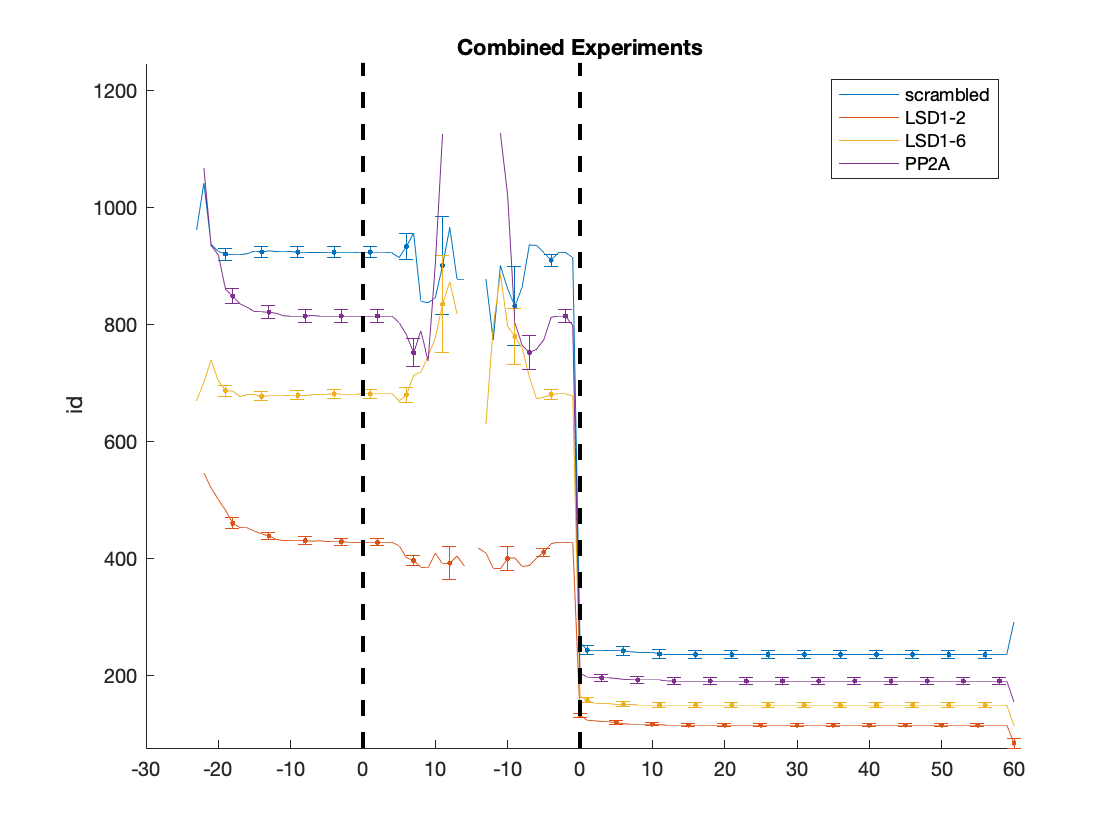

Supplement: S1 File — All existing single features and time series features are contained and accessible from an HTML-based overview file. Extract the archive to a folder of your choice and open the HTML file in the root directory using any web browser. (ZIP) [file pone.0270923.s022.zip › Plots/LSD1_FusedProjects_CARSync_AdditionalFeatures_id_LinePlots.png]

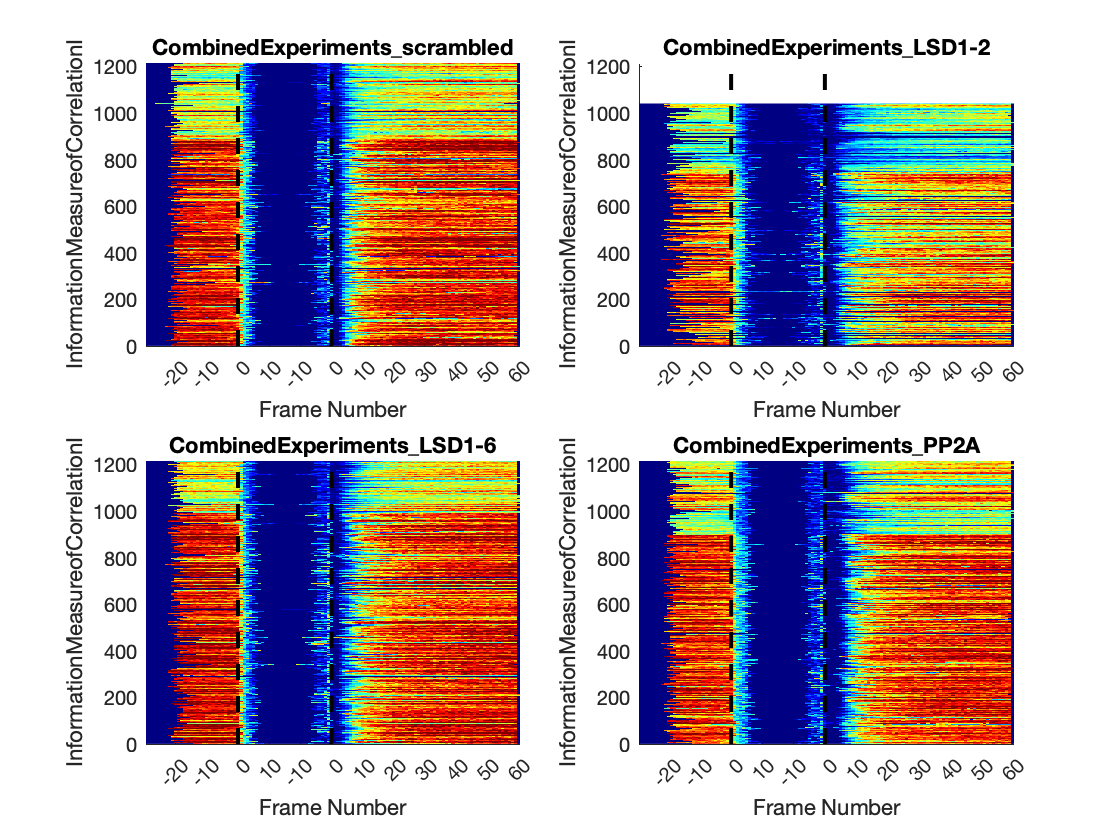

Supplement: S1 File — All existing single features and time series features are contained and accessible from an HTML-based overview file. Extract the archive to a folder of your choice and open the HTML file in the root directory using any web browser. (ZIP) [file pone.0270923.s022.zip › Plots/LSD1_FusedProjects_CARSync_AdditionalFeatures_InformationMeasureofCorrelationI_HeatMaps.png]

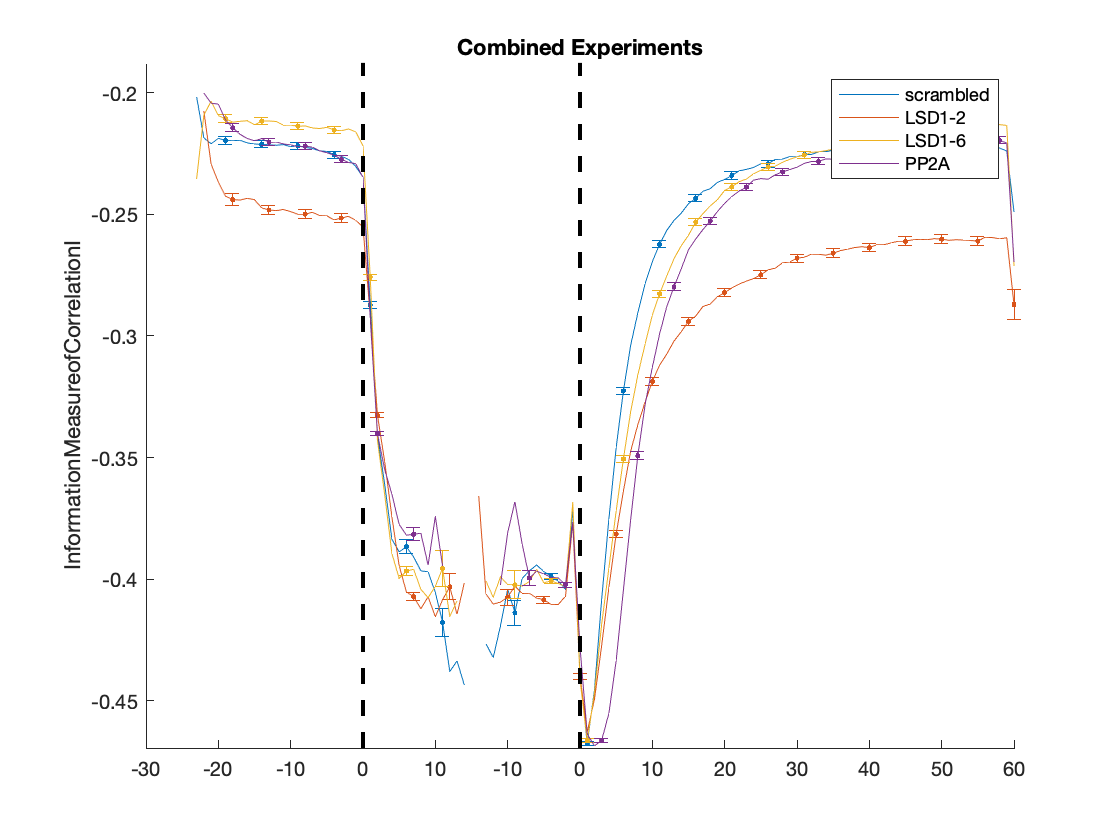

Supplement: S1 File — All existing single features and time series features are contained and accessible from an HTML-based overview file. Extract the archive to a folder of your choice and open the HTML file in the root directory using any web browser. (ZIP) [file pone.0270923.s022.zip › Plots/LSD1_FusedProjects_CARSync_AdditionalFeatures_InformationMeasureofCorrelationI_LinePlots.png]

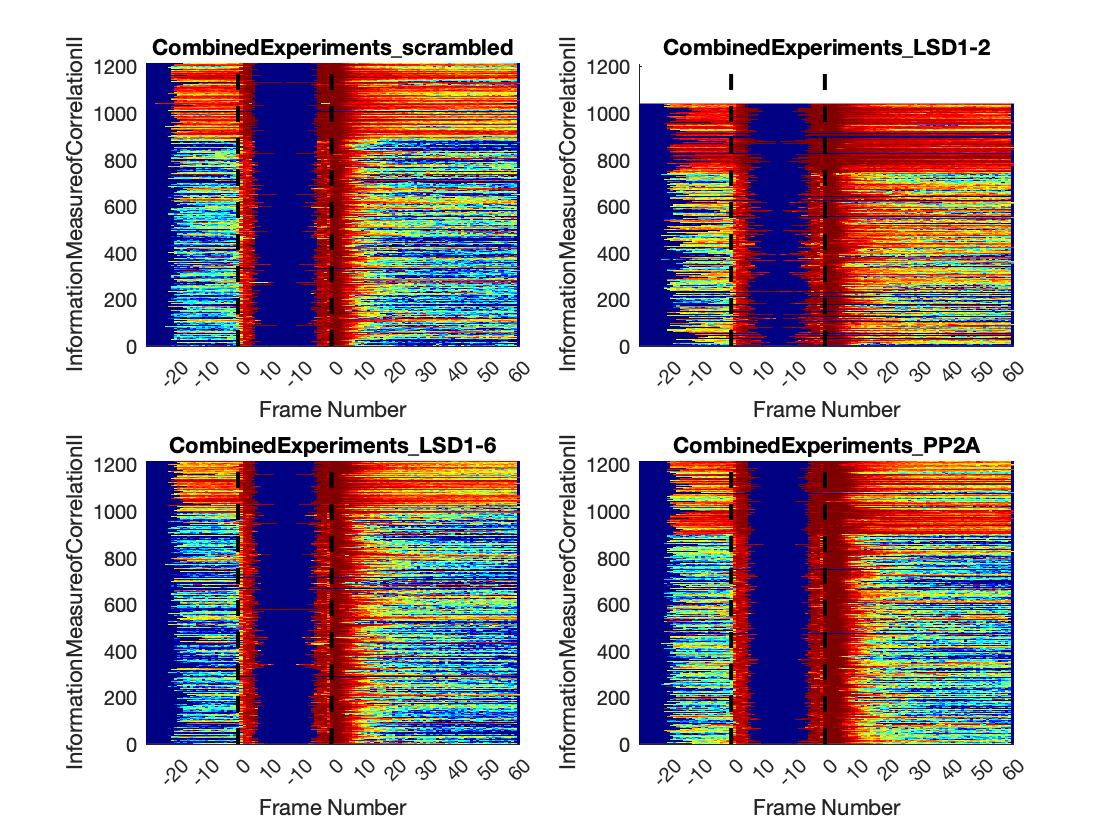

Supplement: S1 File — All existing single features and time series features are contained and accessible from an HTML-based overview file. Extract the archive to a folder of your choice and open the HTML file in the root directory using any web browser. (ZIP) [file pone.0270923.s022.zip › Plots/LSD1_FusedProjects_CARSync_AdditionalFeatures_InformationMeasureofCorrelationII_HeatMaps.png]

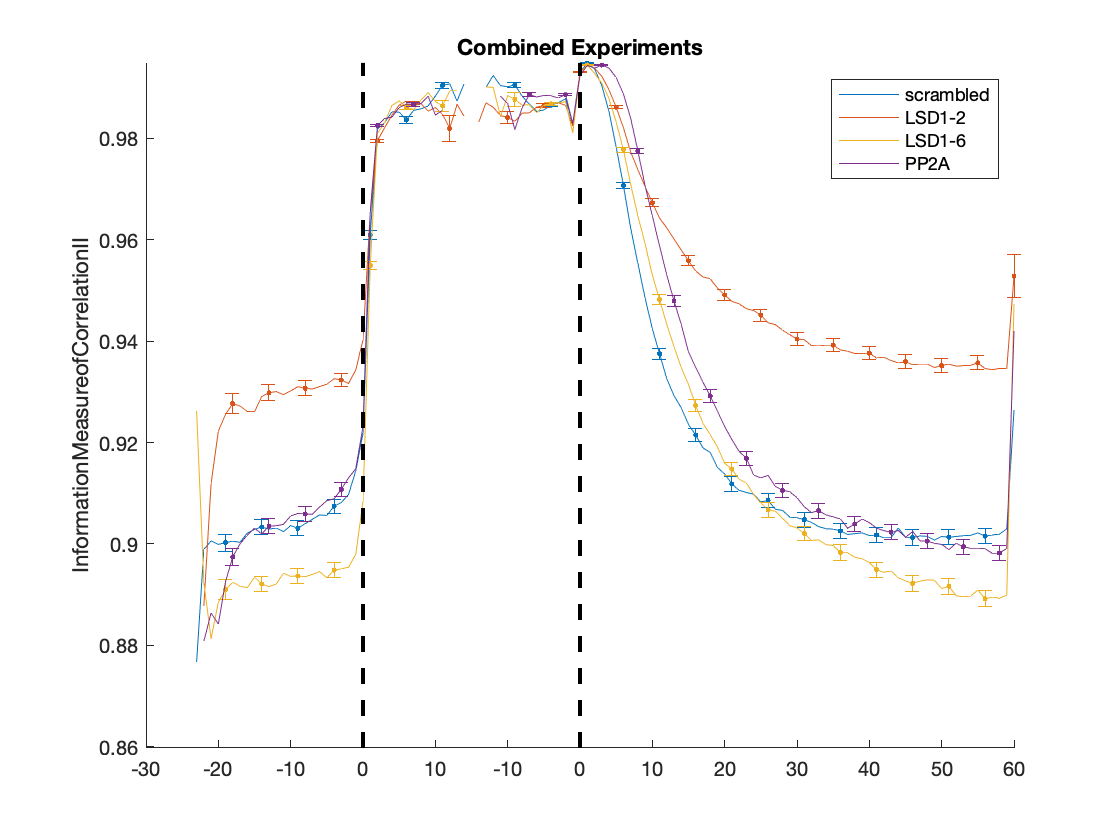

Supplement: S1 File — All existing single features and time series features are contained and accessible from an HTML-based overview file. Extract the archive to a folder of your choice and open the HTML file in the root directory using any web browser. (ZIP) [file pone.0270923.s022.zip › Plots/LSD1_FusedProjects_CARSync_AdditionalFeatures_InformationMeasureofCorrelationII_LinePlots.png]

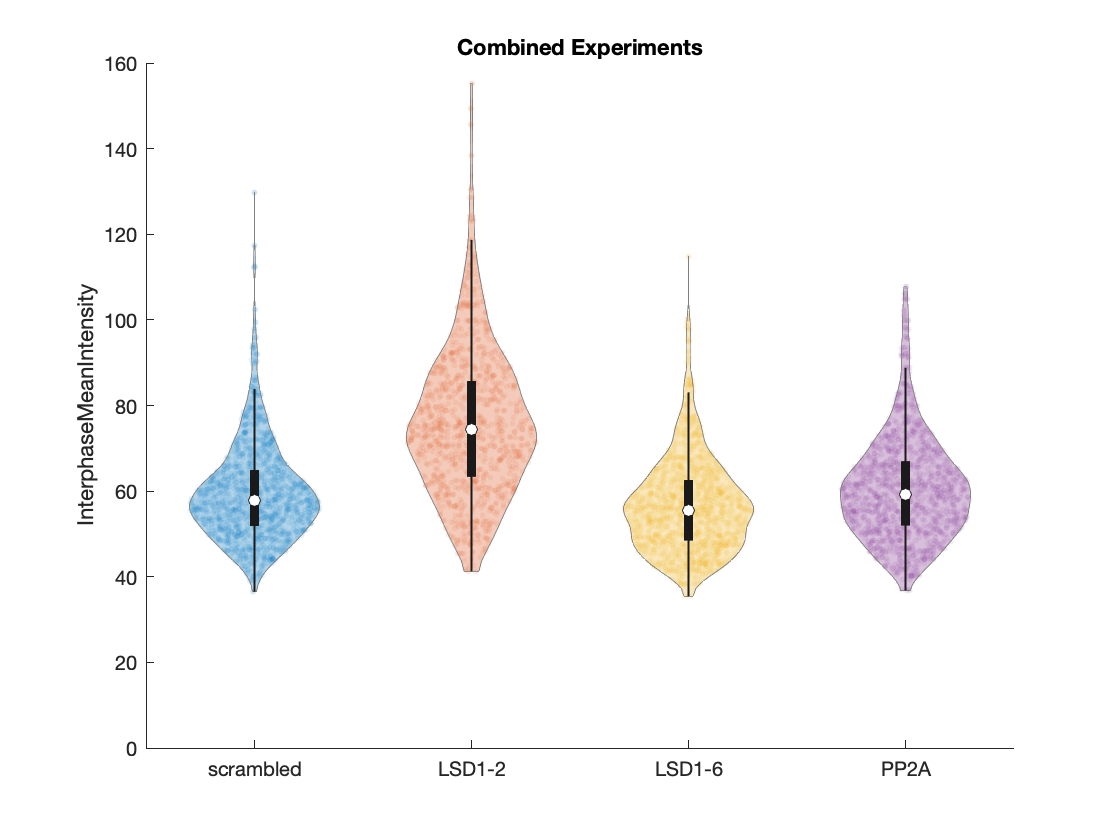

Supplement: S1 File — All existing single features and time series features are contained and accessible from an HTML-based overview file. Extract the archive to a folder of your choice and open the HTML file in the root directory using any web browser. (ZIP) [file pone.0270923.s022.zip › Plots/LSD1_FusedProjects_CARSync_AdditionalFeatures_InterphaseMeanIntensity_BoxPlots.png]

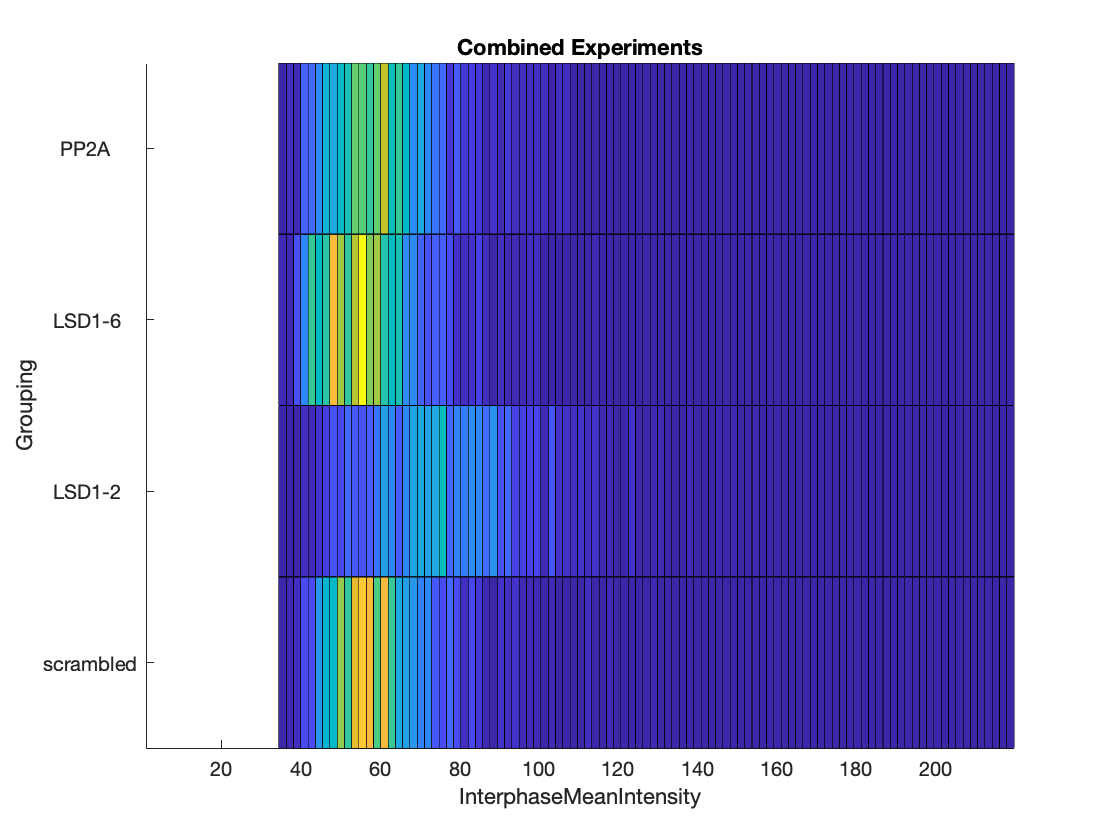

Supplement: S1 File — All existing single features and time series features are contained and accessible from an HTML-based overview file. Extract the archive to a folder of your choice and open the HTML file in the root directory using any web browser. (ZIP) [file pone.0270923.s022.zip › Plots/LSD1_FusedProjects_CARSync_AdditionalFeatures_InterphaseMeanIntensity_Histograms.png]

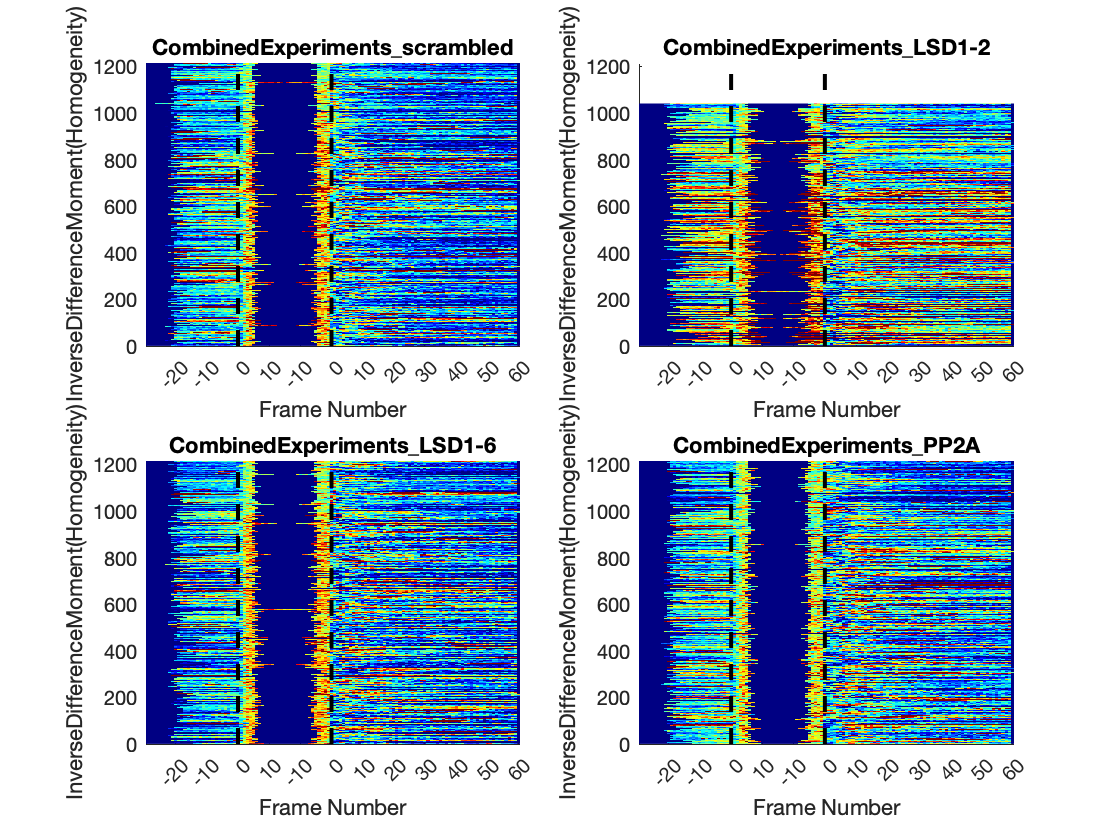

Supplement: S1 File — All existing single features and time series features are contained and accessible from an HTML-based overview file. Extract the archive to a folder of your choice and open the HTML file in the root directory using any web browser. (ZIP) [file pone.0270923.s022.zip › Plots/LSD1_FusedProjects_CARSync_AdditionalFeatures_InverseDifferenceMoment(Homogeneity)_HeatMaps.png]

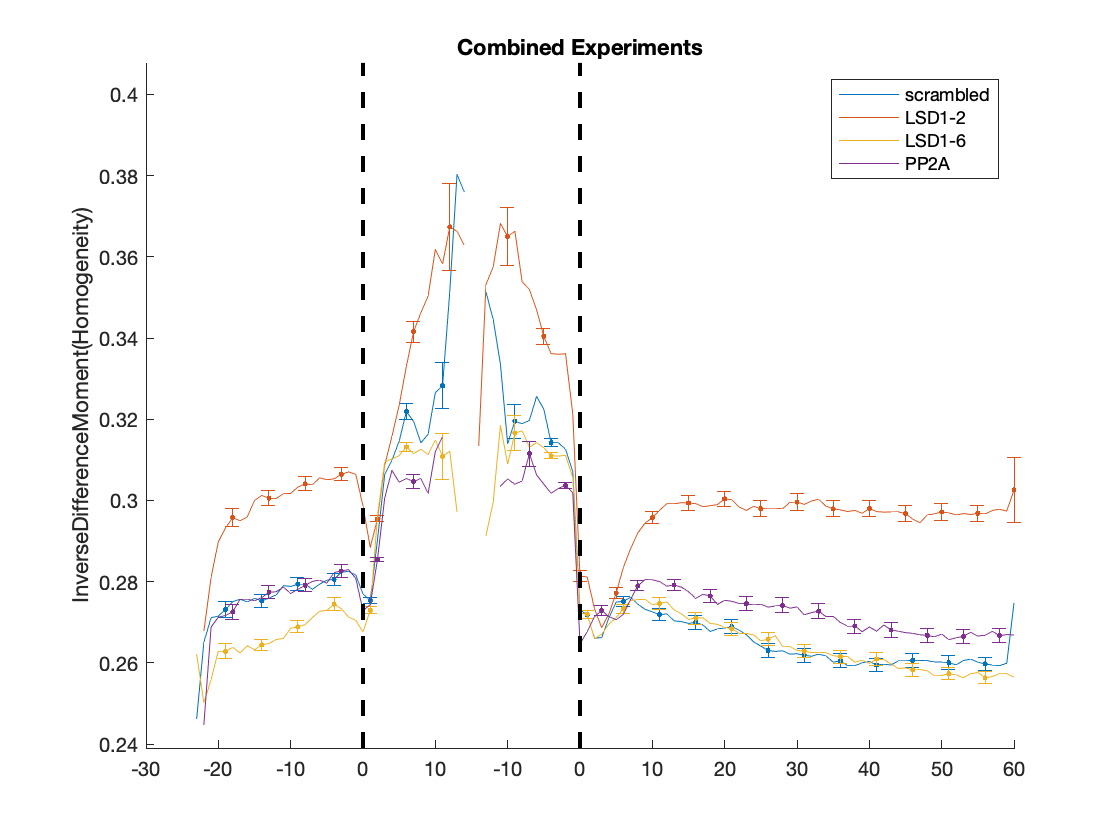

Supplement: S1 File — All existing single features and time series features are contained and accessible from an HTML-based overview file. Extract the archive to a folder of your choice and open the HTML file in the root directory using any web browser. (ZIP) [file pone.0270923.s022.zip › Plots/LSD1_FusedProjects_CARSync_AdditionalFeatures_InverseDifferenceMoment(Homogeneity)_LinePlots.png]

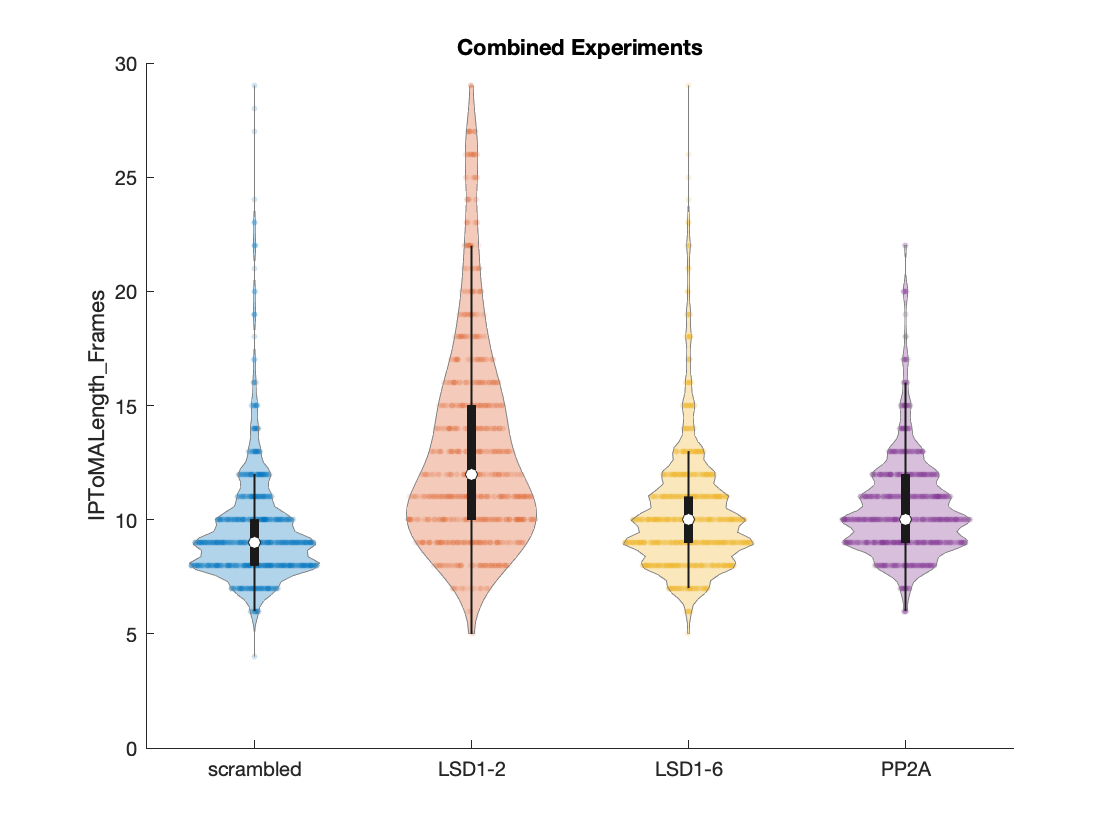

Supplement: S1 File — All existing single features and time series features are contained and accessible from an HTML-based overview file. Extract the archive to a folder of your choice and open the HTML file in the root directory using any web browser. (ZIP) [file pone.0270923.s022.zip › Plots/LSD1_FusedProjects_CARSync_AdditionalFeatures_IPToMALength_Frames_BoxPlots.png]

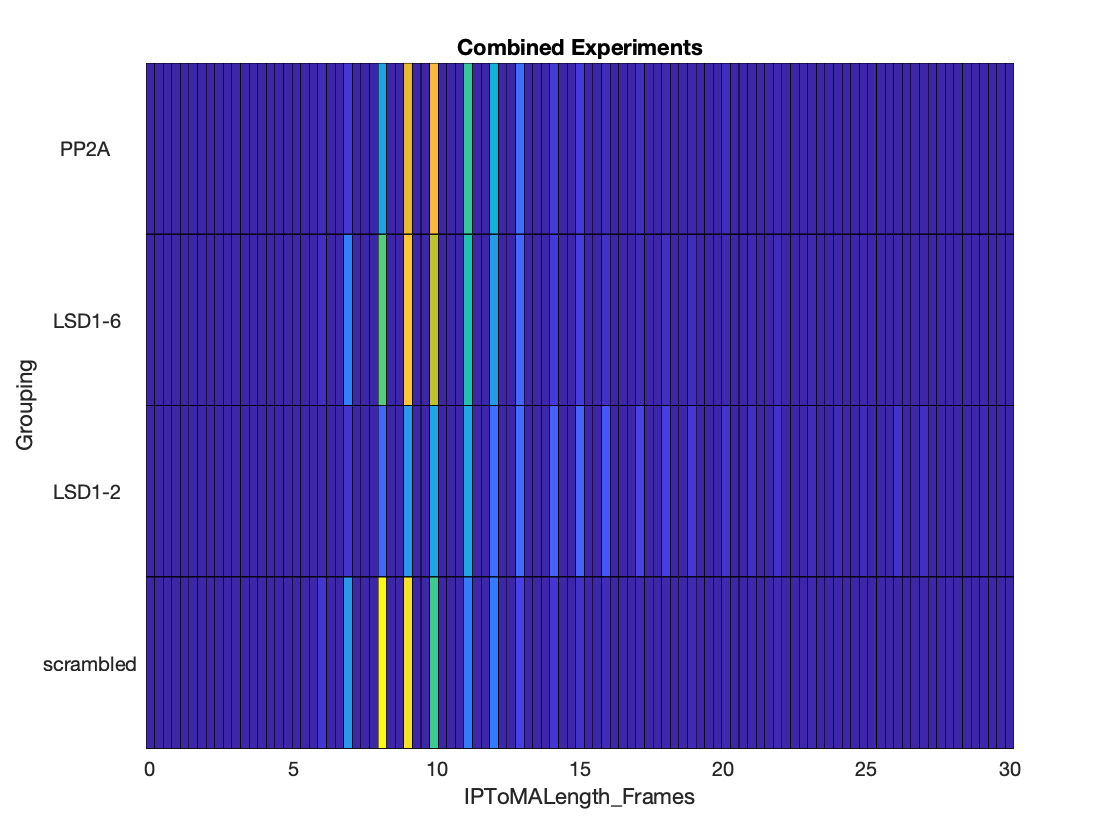

Supplement: S1 File — All existing single features and time series features are contained and accessible from an HTML-based overview file. Extract the archive to a folder of your choice and open the HTML file in the root directory using any web browser. (ZIP) [file pone.0270923.s022.zip › Plots/LSD1_FusedProjects_CARSync_AdditionalFeatures_IPToMALength_Frames_Histograms.png]

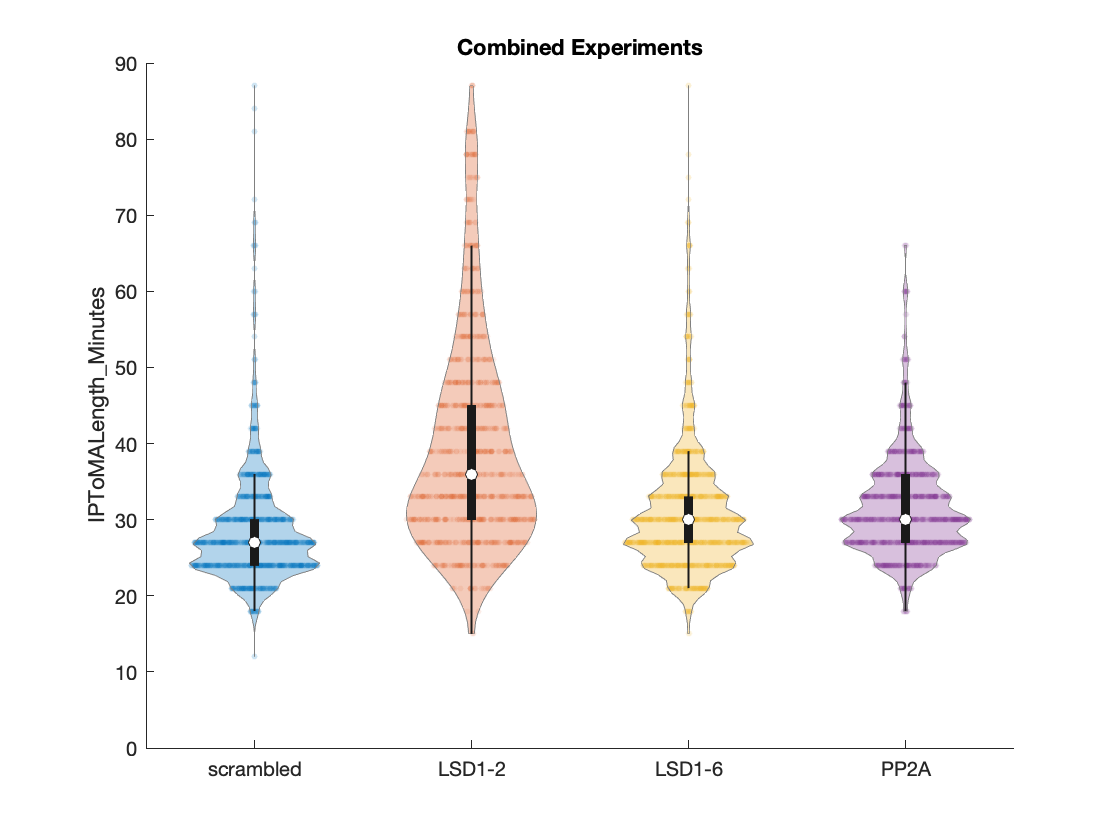

Supplement: S1 File — All existing single features and time series features are contained and accessible from an HTML-based overview file. Extract the archive to a folder of your choice and open the HTML file in the root directory using any web browser. (ZIP) [file pone.0270923.s022.zip › Plots/LSD1_FusedProjects_CARSync_AdditionalFeatures_IPToMALength_Minutes_BoxPlots.png]

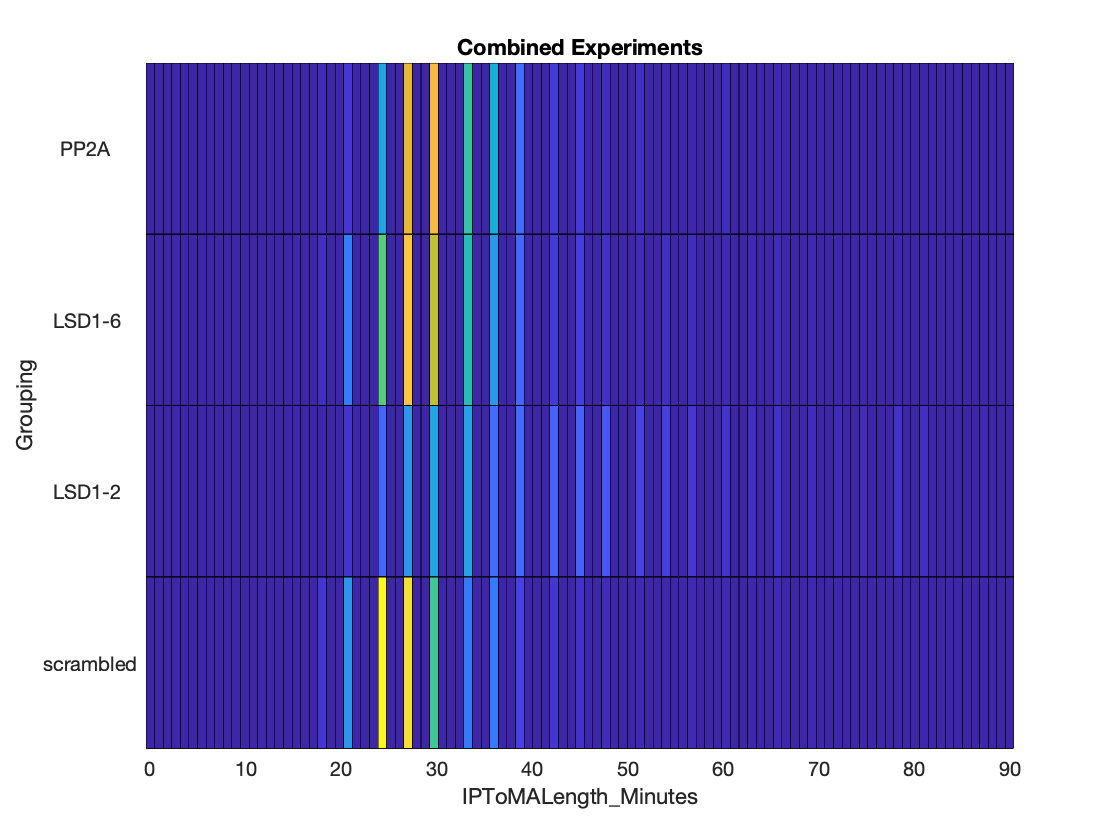

Supplement: S1 File — All existing single features and time series features are contained and accessible from an HTML-based overview file. Extract the archive to a folder of your choice and open the HTML file in the root directory using any web browser. (ZIP) [file pone.0270923.s022.zip › Plots/LSD1_FusedProjects_CARSync_AdditionalFeatures_IPToMALength_Minutes_Histograms.png]

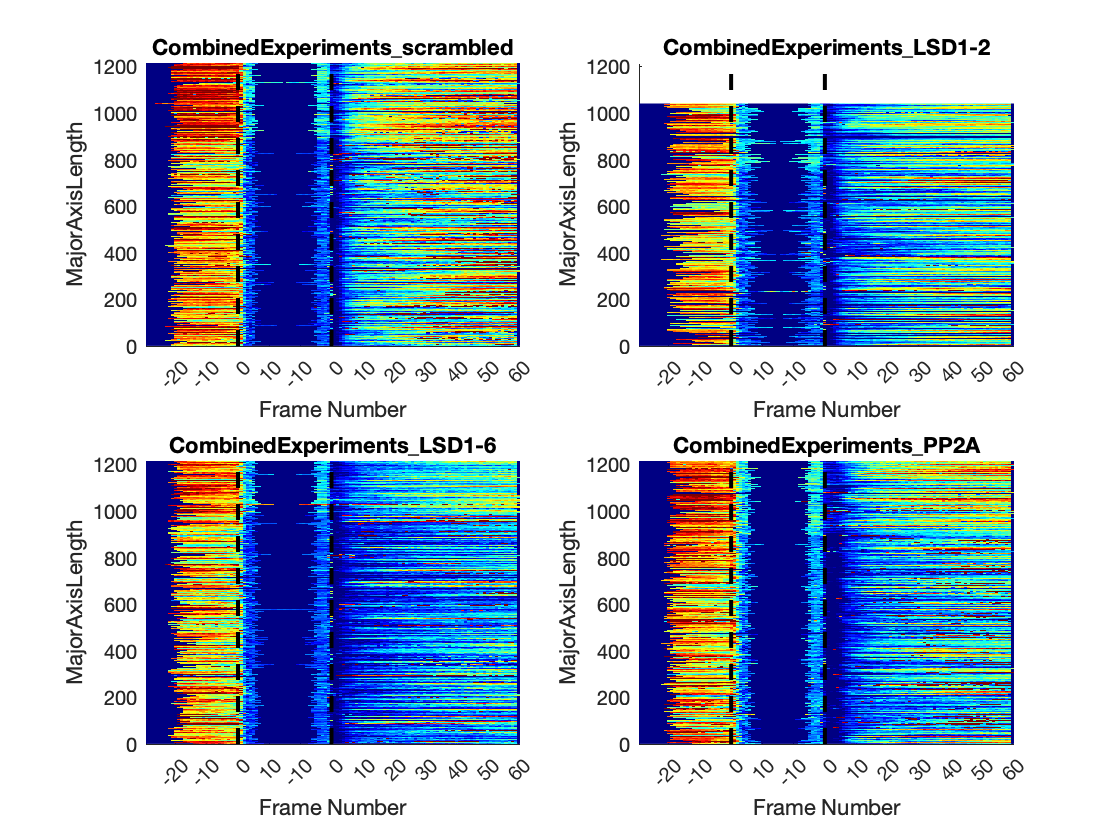

Supplement: S1 File — All existing single features and time series features are contained and accessible from an HTML-based overview file. Extract the archive to a folder of your choice and open the HTML file in the root directory using any web browser. (ZIP) [file pone.0270923.s022.zip › Plots/LSD1_FusedProjects_CARSync_AdditionalFeatures_MajorAxisLength_HeatMaps.png]

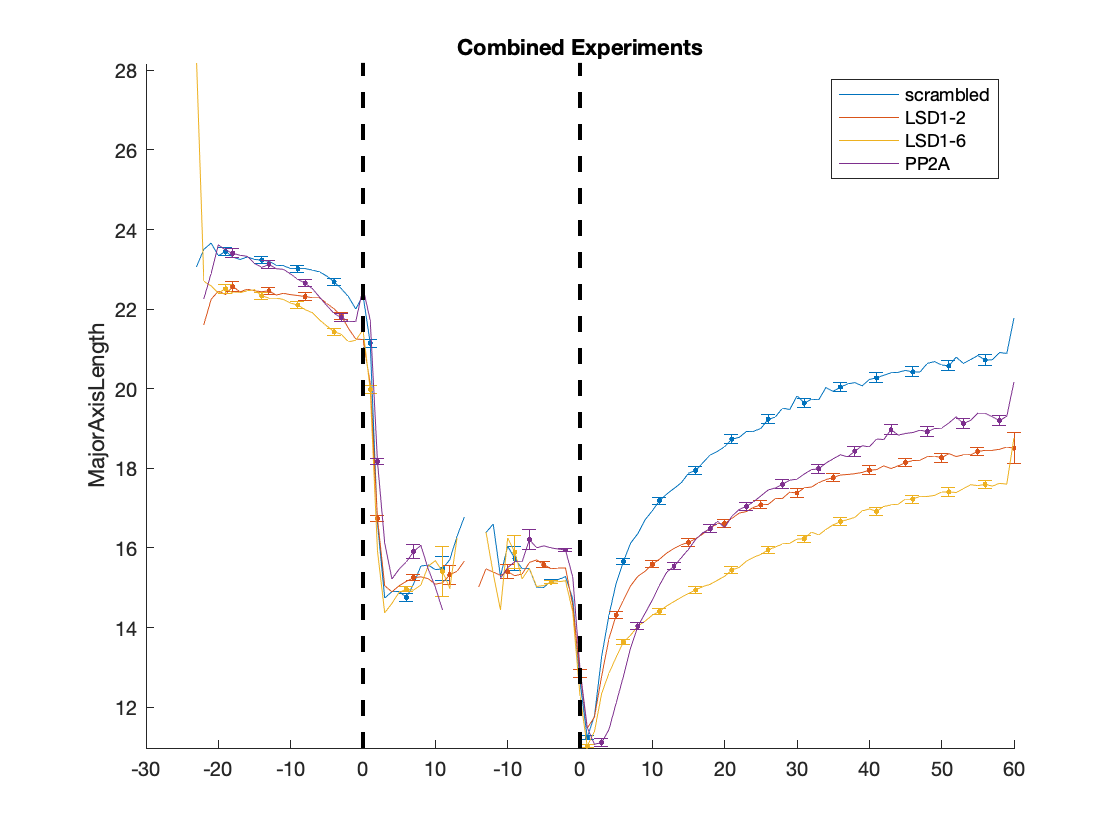

Supplement: S1 File — All existing single features and time series features are contained and accessible from an HTML-based overview file. Extract the archive to a folder of your choice and open the HTML file in the root directory using any web browser. (ZIP) [file pone.0270923.s022.zip › Plots/LSD1_FusedProjects_CARSync_AdditionalFeatures_MajorAxisLength_LinePlots.png]

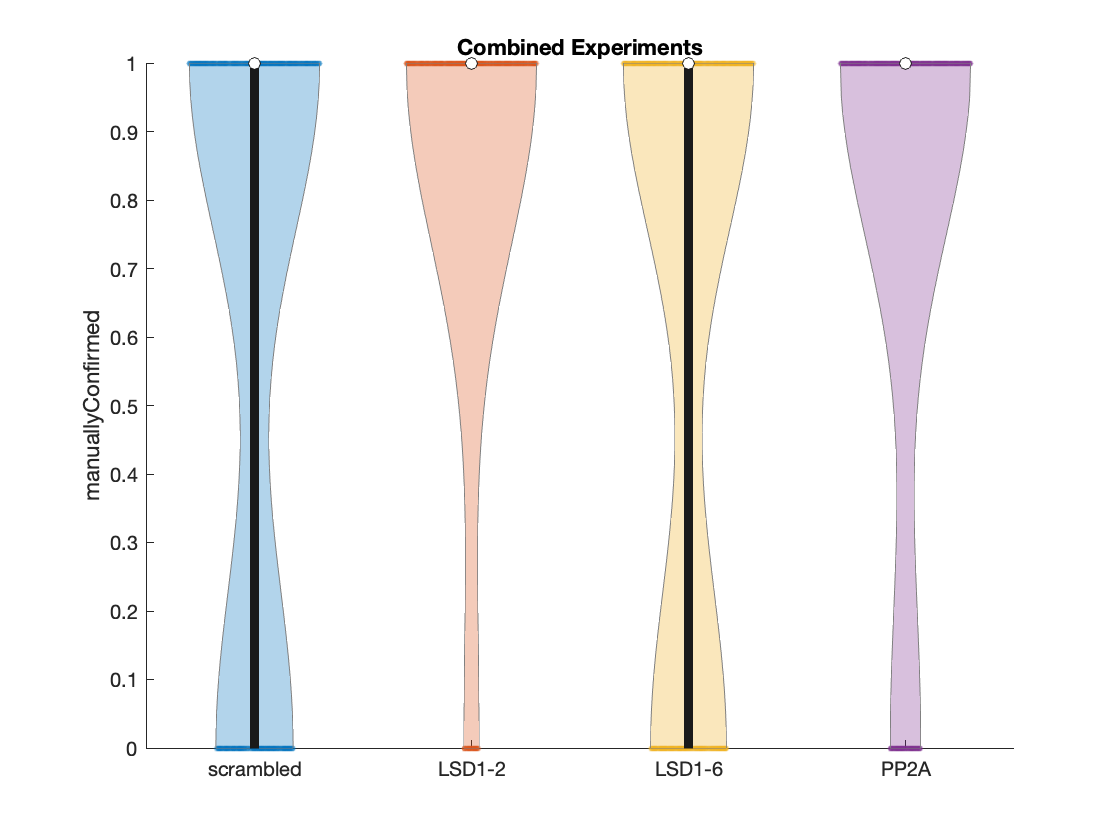

Supplement: S1 File — All existing single features and time series features are contained and accessible from an HTML-based overview file. Extract the archive to a folder of your choice and open the HTML file in the root directory using any web browser. (ZIP) [file pone.0270923.s022.zip › Plots/LSD1_FusedProjects_CARSync_AdditionalFeatures_manuallyConfirmed_BoxPlots.png]

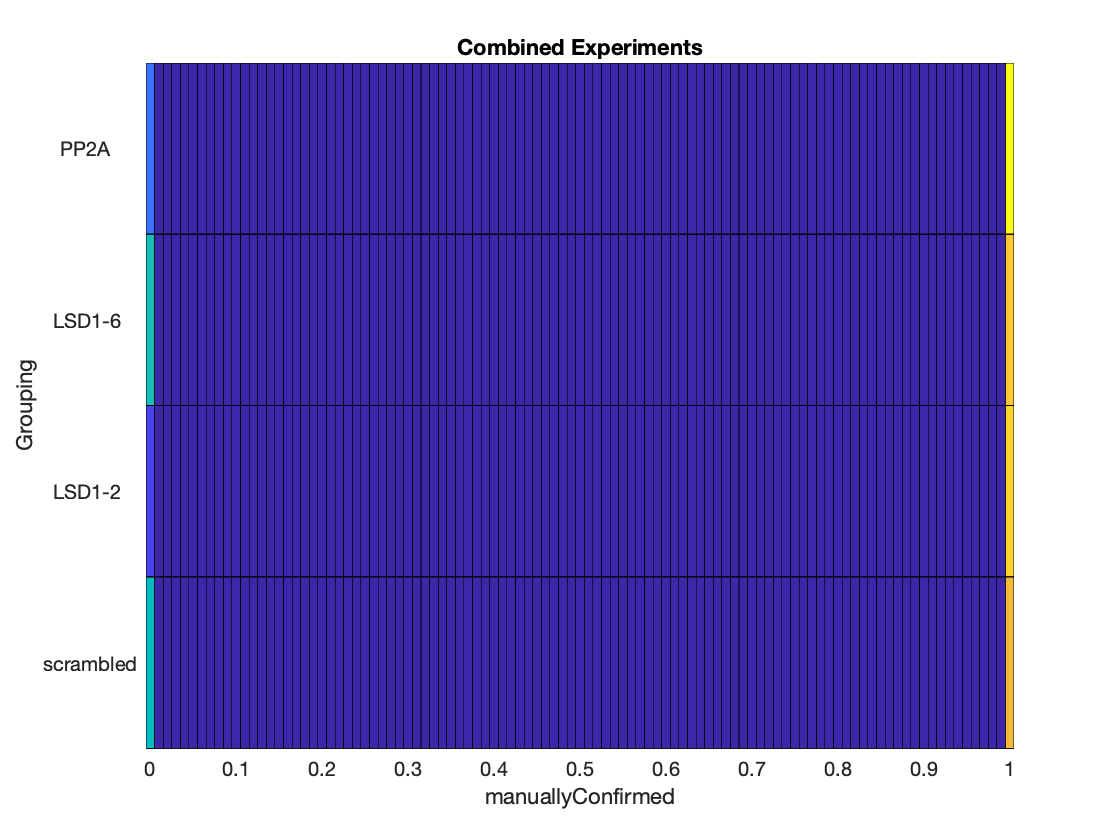

Supplement: S1 File — All existing single features and time series features are contained and accessible from an HTML-based overview file. Extract the archive to a folder of your choice and open the HTML file in the root directory using any web browser. (ZIP) [file pone.0270923.s022.zip › Plots/LSD1_FusedProjects_CARSync_AdditionalFeatures_manuallyConfirmed_Histograms.png]

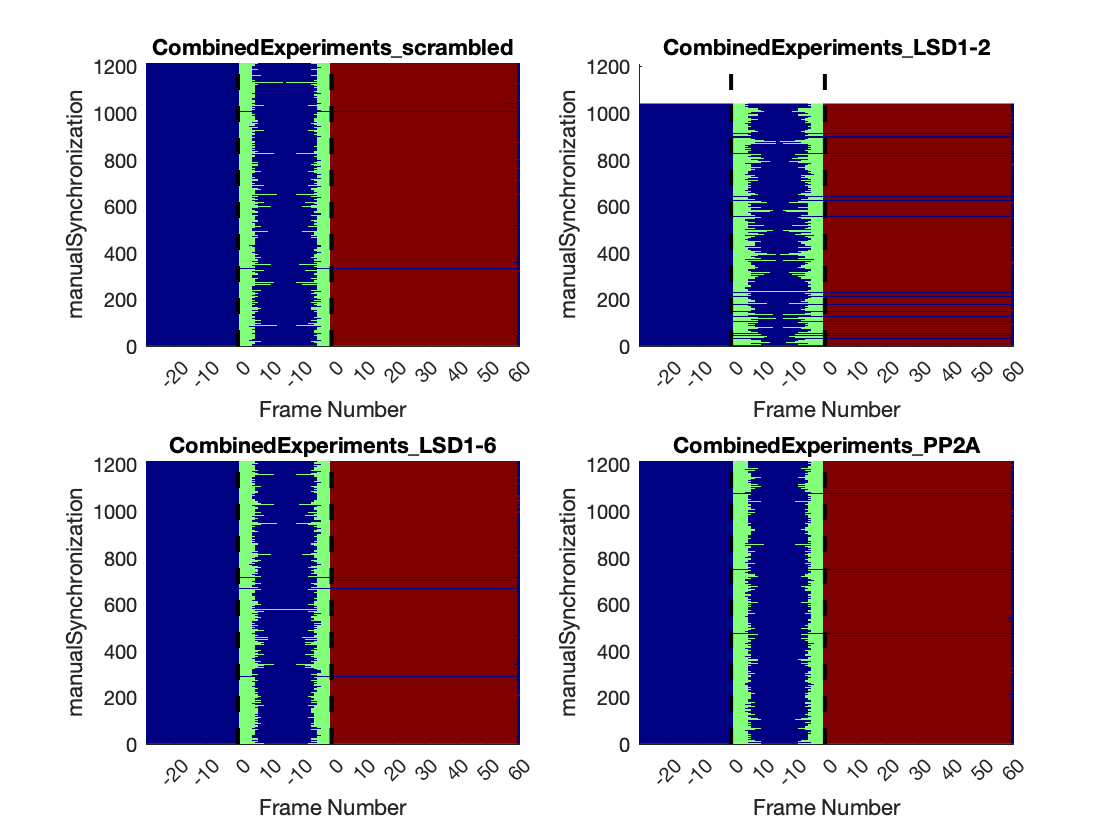

Supplement: S1 File — All existing single features and time series features are contained and accessible from an HTML-based overview file. Extract the archive to a folder of your choice and open the HTML file in the root directory using any web browser. (ZIP) [file pone.0270923.s022.zip › Plots/LSD1_FusedProjects_CARSync_AdditionalFeatures_manualSynchronization_HeatMaps.png]

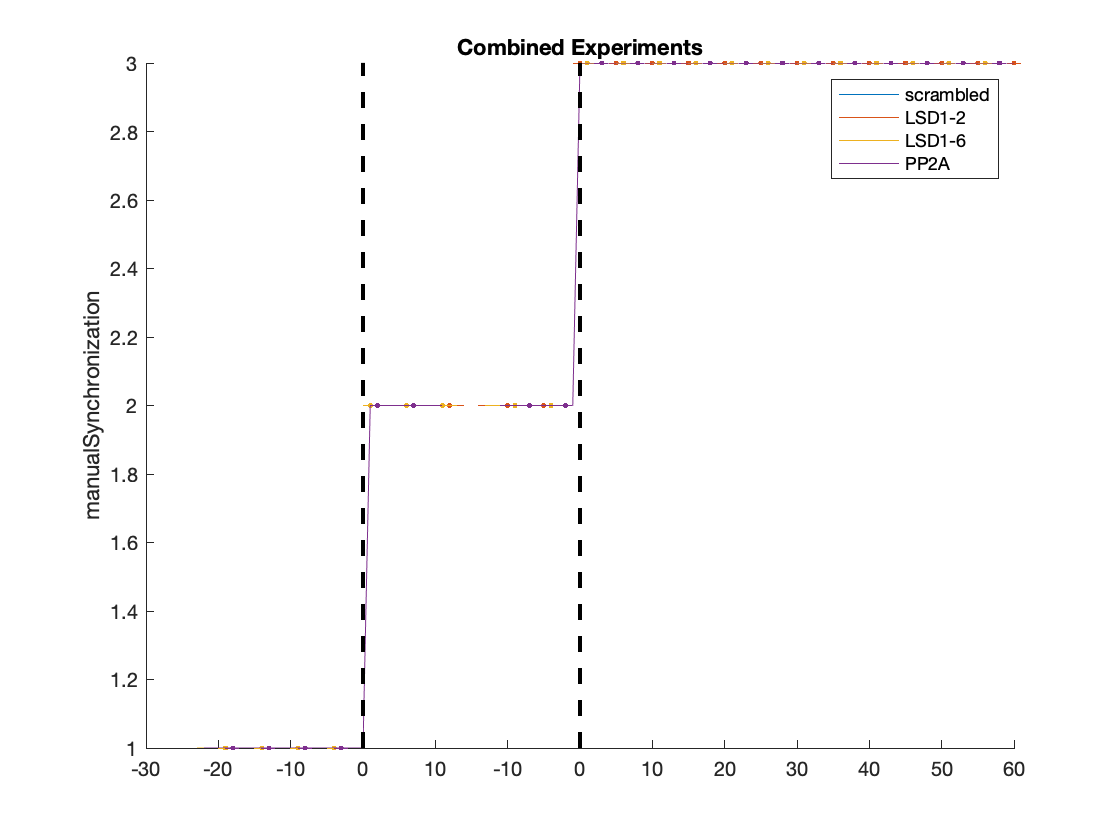

Supplement: S1 File — All existing single features and time series features are contained and accessible from an HTML-based overview file. Extract the archive to a folder of your choice and open the HTML file in the root directory using any web browser. (ZIP) [file pone.0270923.s022.zip › Plots/LSD1_FusedProjects_CARSync_AdditionalFeatures_manualSynchronization_LinePlots.png]

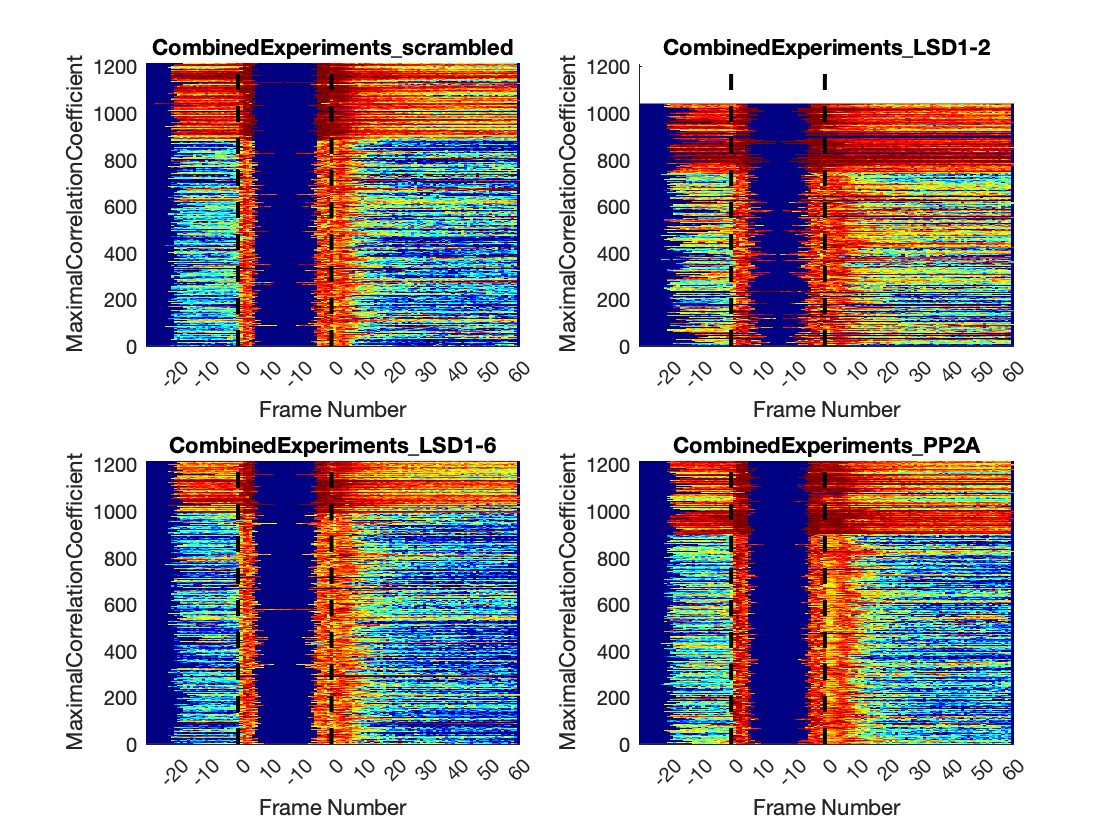

Supplement: S1 File — All existing single features and time series features are contained and accessible from an HTML-based overview file. Extract the archive to a folder of your choice and open the HTML file in the root directory using any web browser. (ZIP) [file pone.0270923.s022.zip › Plots/LSD1_FusedProjects_CARSync_AdditionalFeatures_MaximalCorrelationCoefficient_HeatMaps.png]

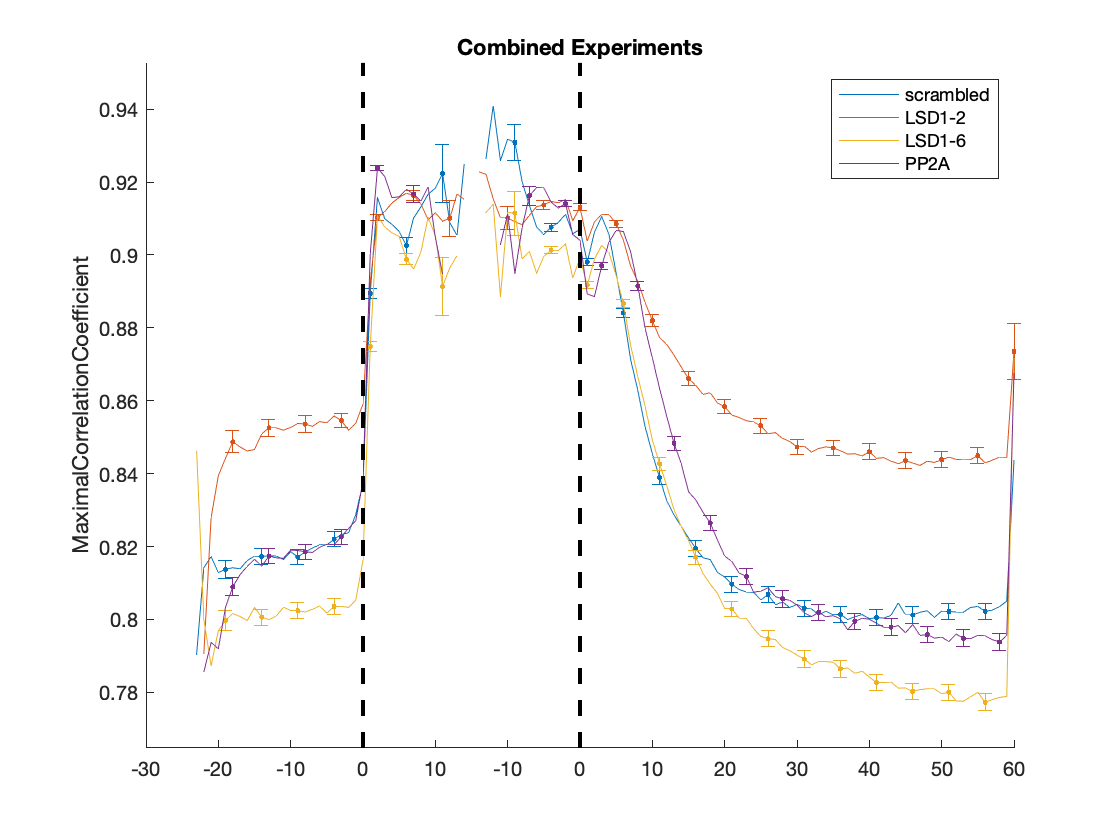

Supplement: S1 File — All existing single features and time series features are contained and accessible from an HTML-based overview file. Extract the archive to a folder of your choice and open the HTML file in the root directory using any web browser. (ZIP) [file pone.0270923.s022.zip › Plots/LSD1_FusedProjects_CARSync_AdditionalFeatures_MaximalCorrelationCoefficient_LinePlots.png]

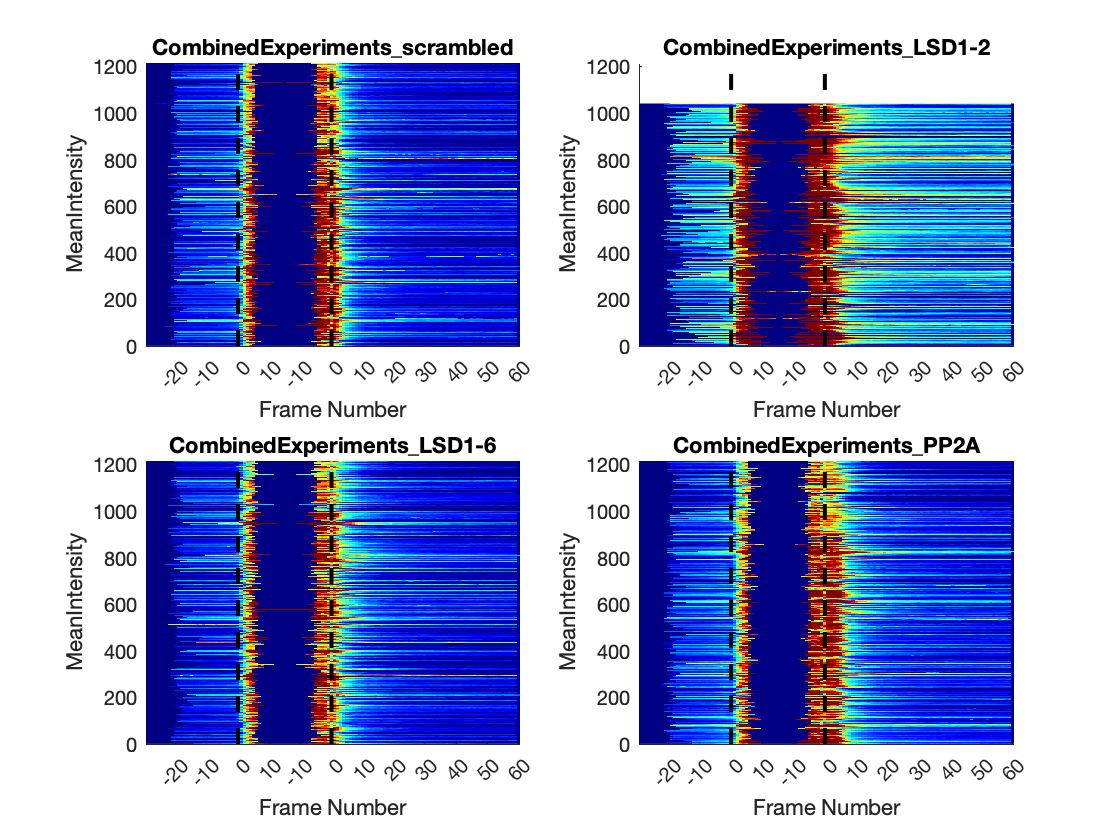

Supplement: S1 File — All existing single features and time series features are contained and accessible from an HTML-based overview file. Extract the archive to a folder of your choice and open the HTML file in the root directory using any web browser. (ZIP) [file pone.0270923.s022.zip › Plots/LSD1_FusedProjects_CARSync_AdditionalFeatures_MeanIntensity_HeatMaps.png]

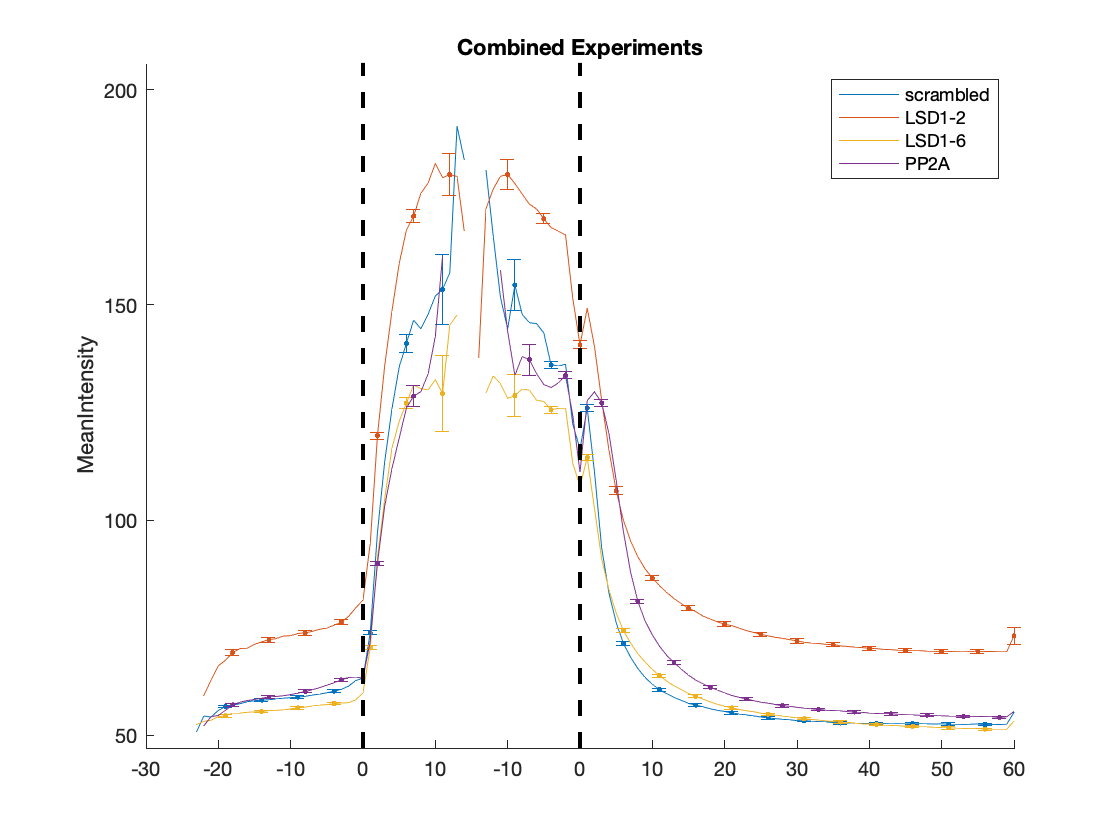

Supplement: S1 File — All existing single features and time series features are contained and accessible from an HTML-based overview file. Extract the archive to a folder of your choice and open the HTML file in the root directory using any web browser. (ZIP) [file pone.0270923.s022.zip › Plots/LSD1_FusedProjects_CARSync_AdditionalFeatures_MeanIntensity_LinePlots.png]

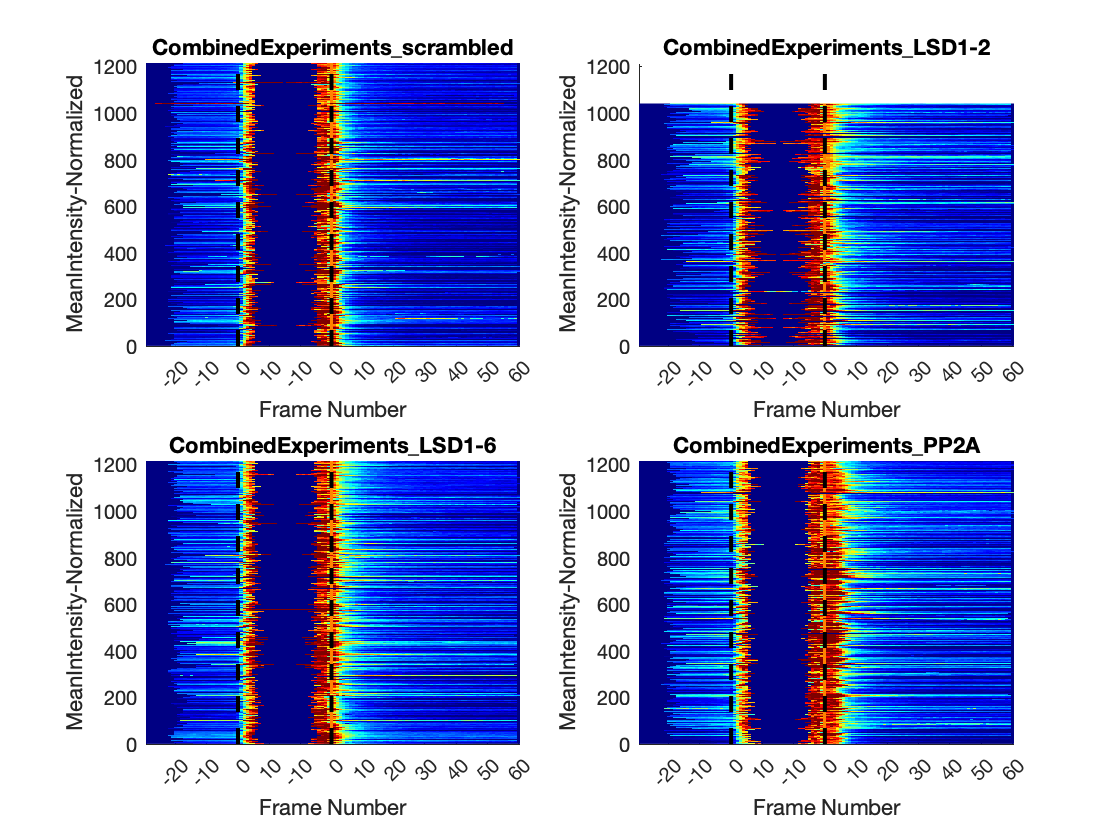

Supplement: S1 File — All existing single features and time series features are contained and accessible from an HTML-based overview file. Extract the archive to a folder of your choice and open the HTML file in the root directory using any web browser. (ZIP) [file pone.0270923.s022.zip › Plots/LSD1_FusedProjects_CARSync_AdditionalFeatures_MeanIntensity-Normalized_HeatMaps.png]

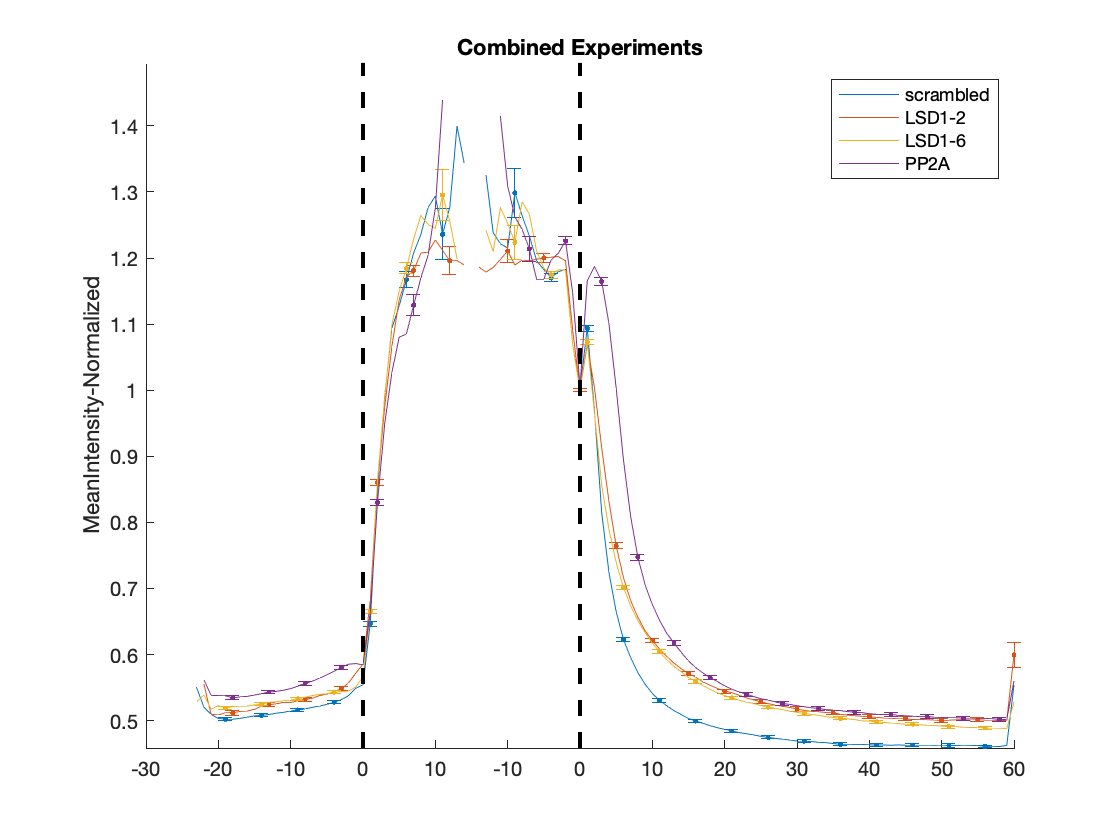

Supplement: S1 File — All existing single features and time series features are contained and accessible from an HTML-based overview file. Extract the archive to a folder of your choice and open the HTML file in the root directory using any web browser. (ZIP) [file pone.0270923.s022.zip › Plots/LSD1_FusedProjects_CARSync_AdditionalFeatures_MeanIntensity-Normalized_LinePlots.png]

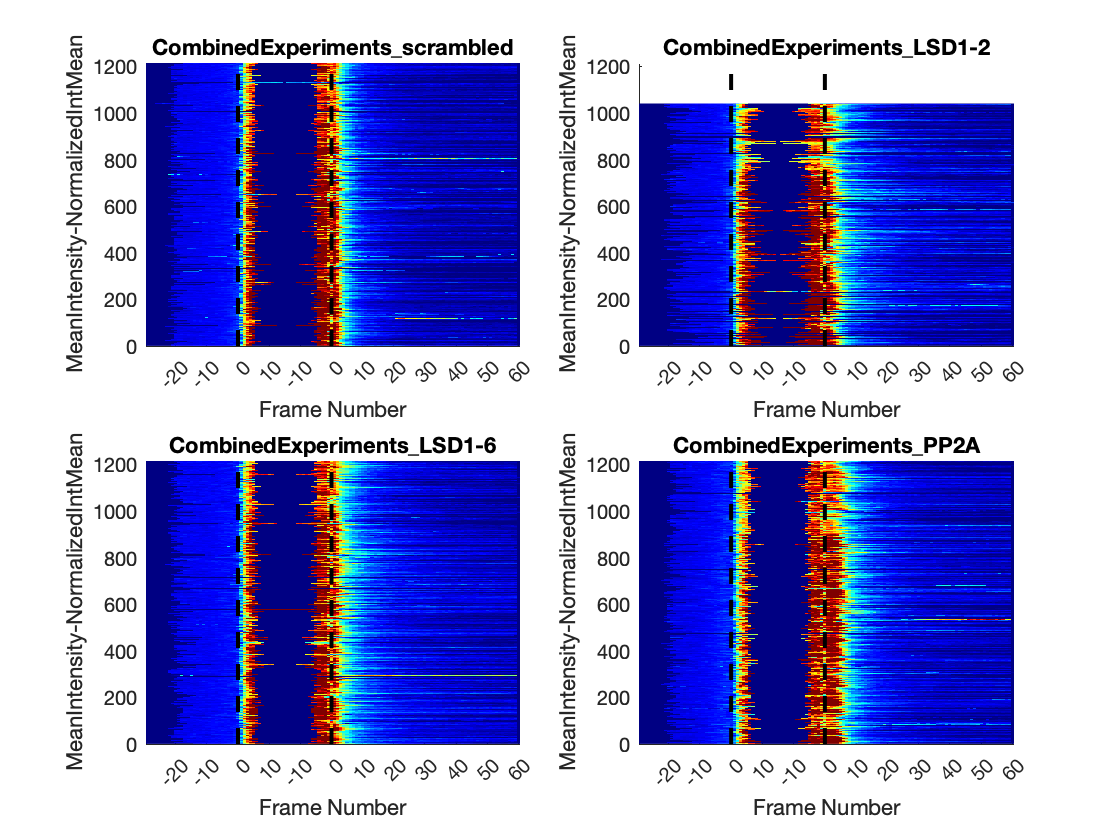

Supplement: S1 File — All existing single features and time series features are contained and accessible from an HTML-based overview file. Extract the archive to a folder of your choice and open the HTML file in the root directory using any web browser. (ZIP) [file pone.0270923.s022.zip › Plots/LSD1_FusedProjects_CARSync_AdditionalFeatures_MeanIntensity-NormalizedIntMean_HeatMaps.png]

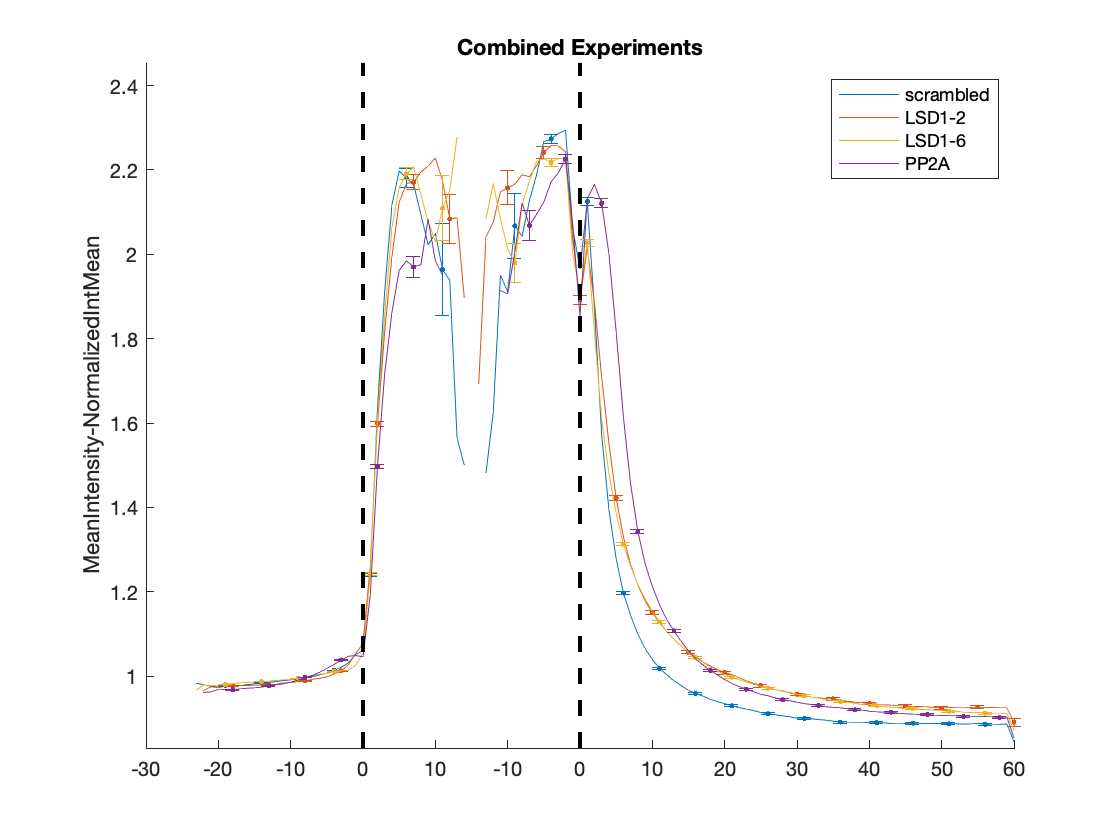

Supplement: S1 File — All existing single features and time series features are contained and accessible from an HTML-based overview file. Extract the archive to a folder of your choice and open the HTML file in the root directory using any web browser. (ZIP) [file pone.0270923.s022.zip › Plots/LSD1_FusedProjects_CARSync_AdditionalFeatures_MeanIntensity-NormalizedIntMean_LinePlots.png]

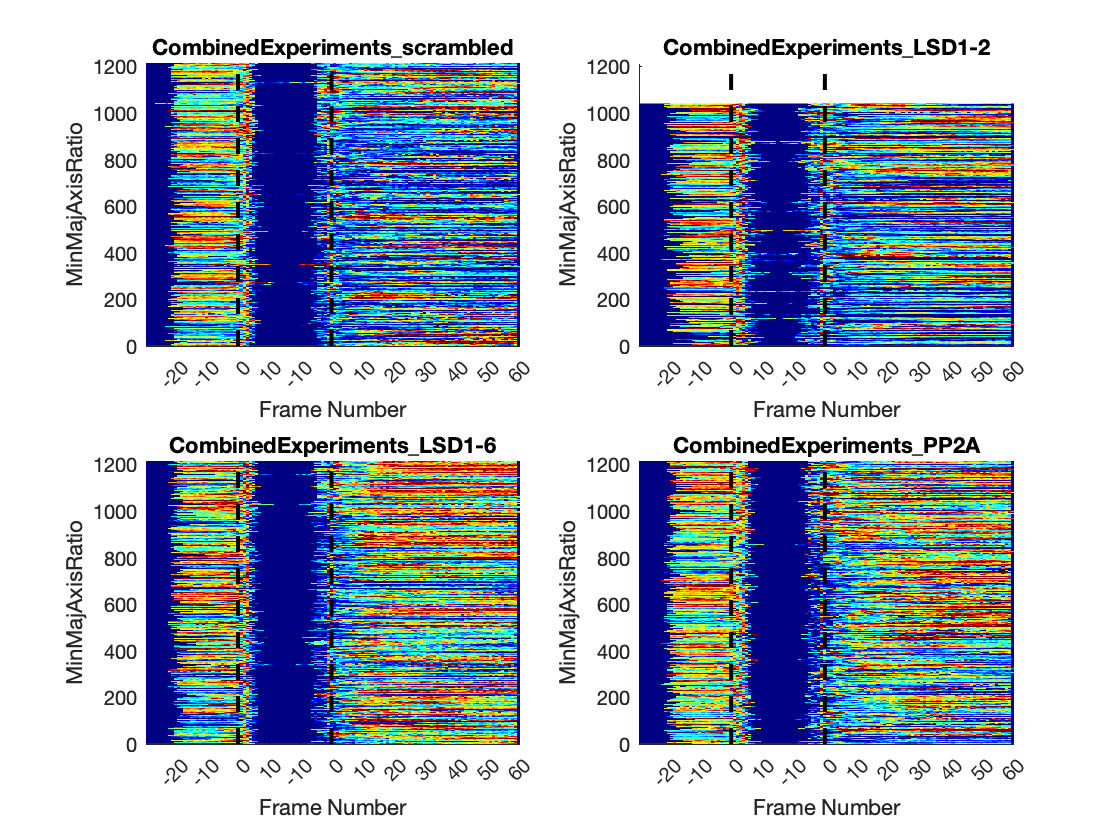

Supplement: S1 File — All existing single features and time series features are contained and accessible from an HTML-based overview file. Extract the archive to a folder of your choice and open the HTML file in the root directory using any web browser. (ZIP) [file pone.0270923.s022.zip › Plots/LSD1_FusedProjects_CARSync_AdditionalFeatures_MinMajAxisRatio_HeatMaps.png]

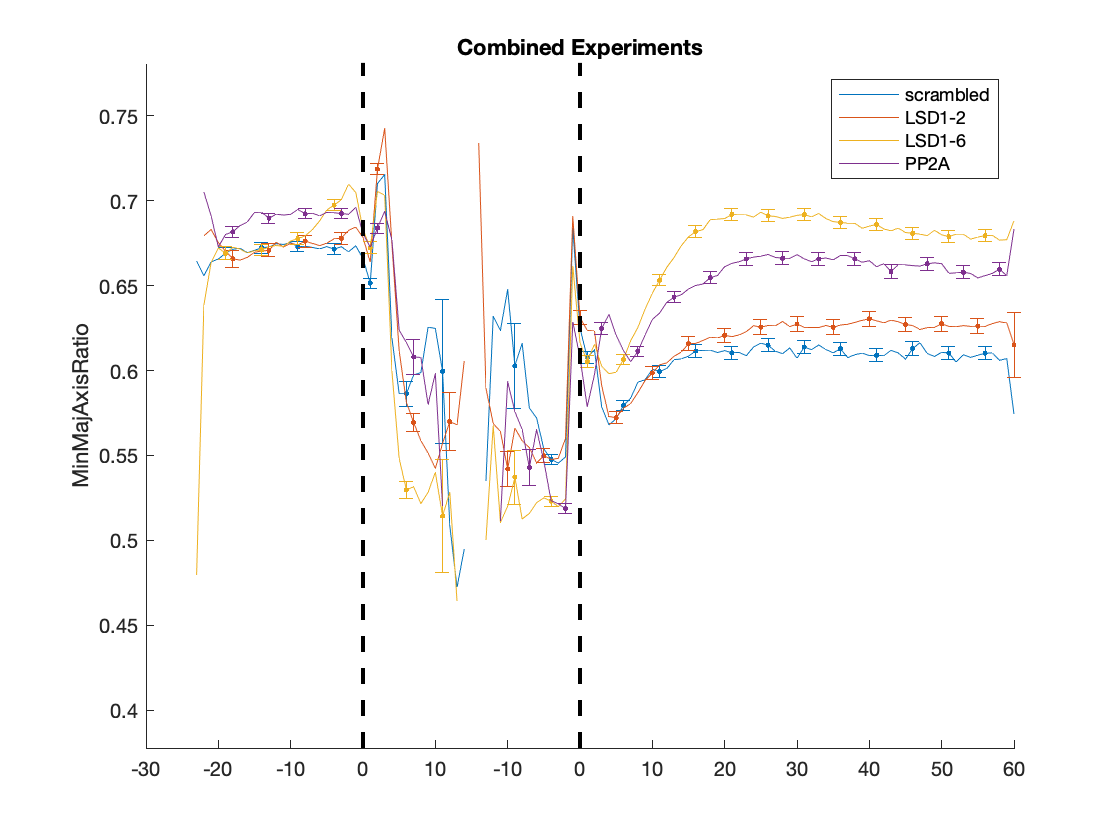

Supplement: S1 File — All existing single features and time series features are contained and accessible from an HTML-based overview file. Extract the archive to a folder of your choice and open the HTML file in the root directory using any web browser. (ZIP) [file pone.0270923.s022.zip › Plots/LSD1_FusedProjects_CARSync_AdditionalFeatures_MinMajAxisRatio_LinePlots.png]

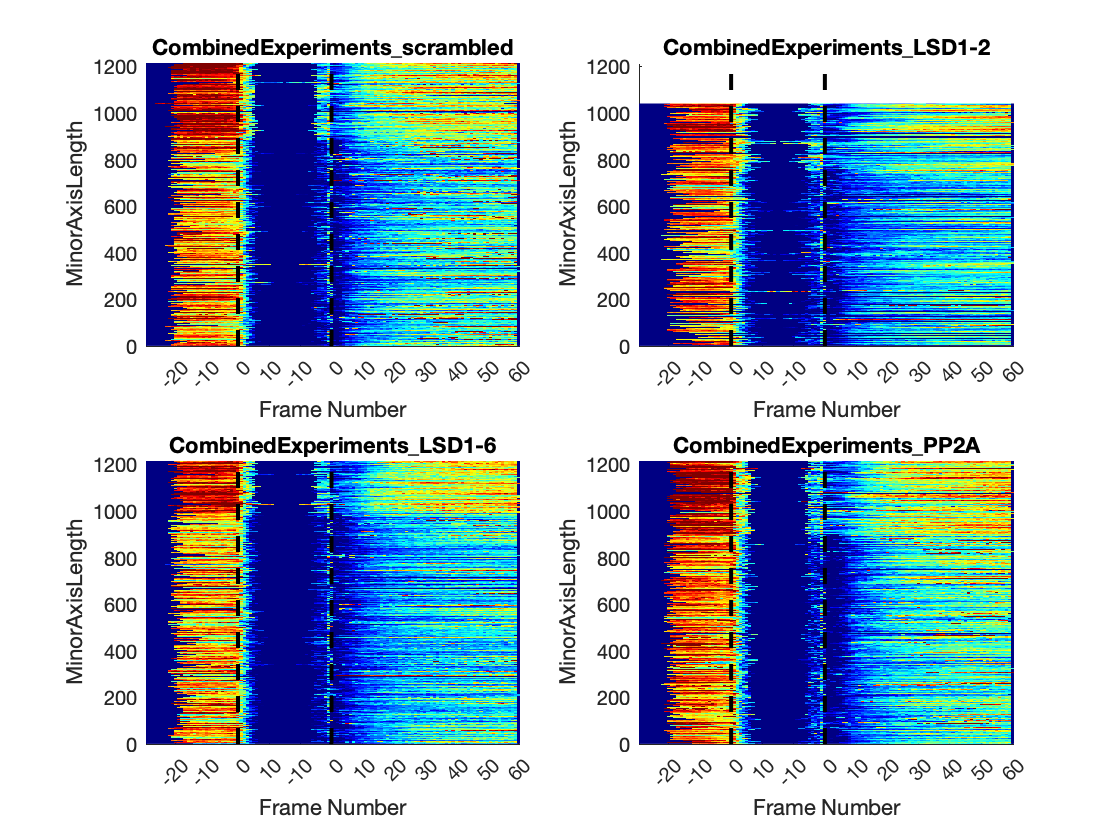

Supplement: S1 File — All existing single features and time series features are contained and accessible from an HTML-based overview file. Extract the archive to a folder of your choice and open the HTML file in the root directory using any web browser. (ZIP) [file pone.0270923.s022.zip › Plots/LSD1_FusedProjects_CARSync_AdditionalFeatures_MinorAxisLength_HeatMaps.png]

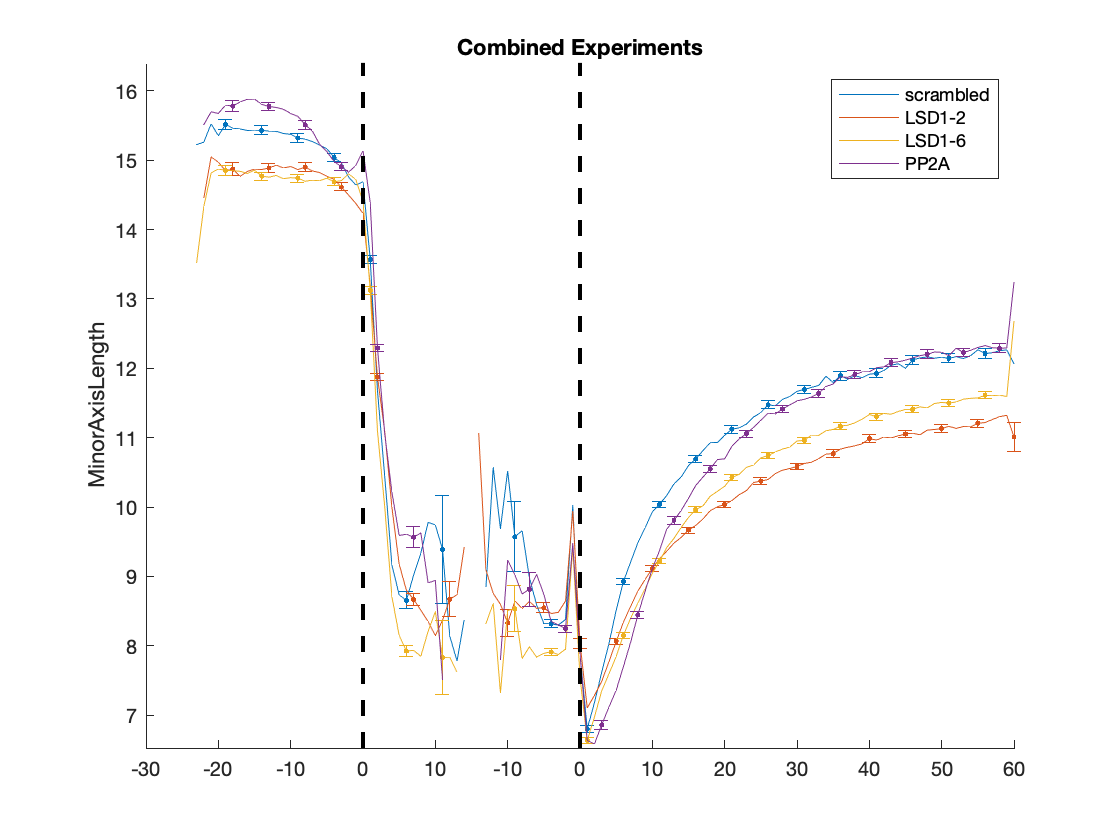

Supplement: S1 File — All existing single features and time series features are contained and accessible from an HTML-based overview file. Extract the archive to a folder of your choice and open the HTML file in the root directory using any web browser. (ZIP) [file pone.0270923.s022.zip › Plots/LSD1_FusedProjects_CARSync_AdditionalFeatures_MinorAxisLength_LinePlots.png]

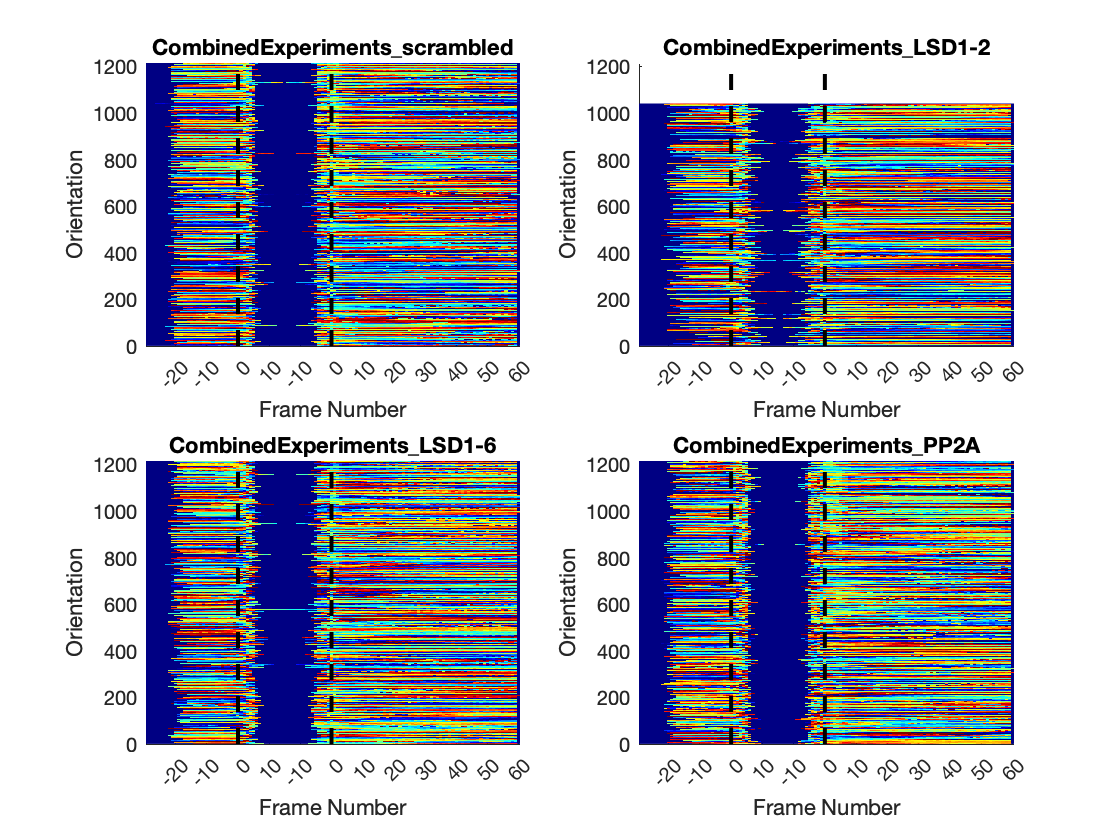

Supplement: S1 File — All existing single features and time series features are contained and accessible from an HTML-based overview file. Extract the archive to a folder of your choice and open the HTML file in the root directory using any web browser. (ZIP) [file pone.0270923.s022.zip › Plots/LSD1_FusedProjects_CARSync_AdditionalFeatures_Orientation_HeatMaps.png]

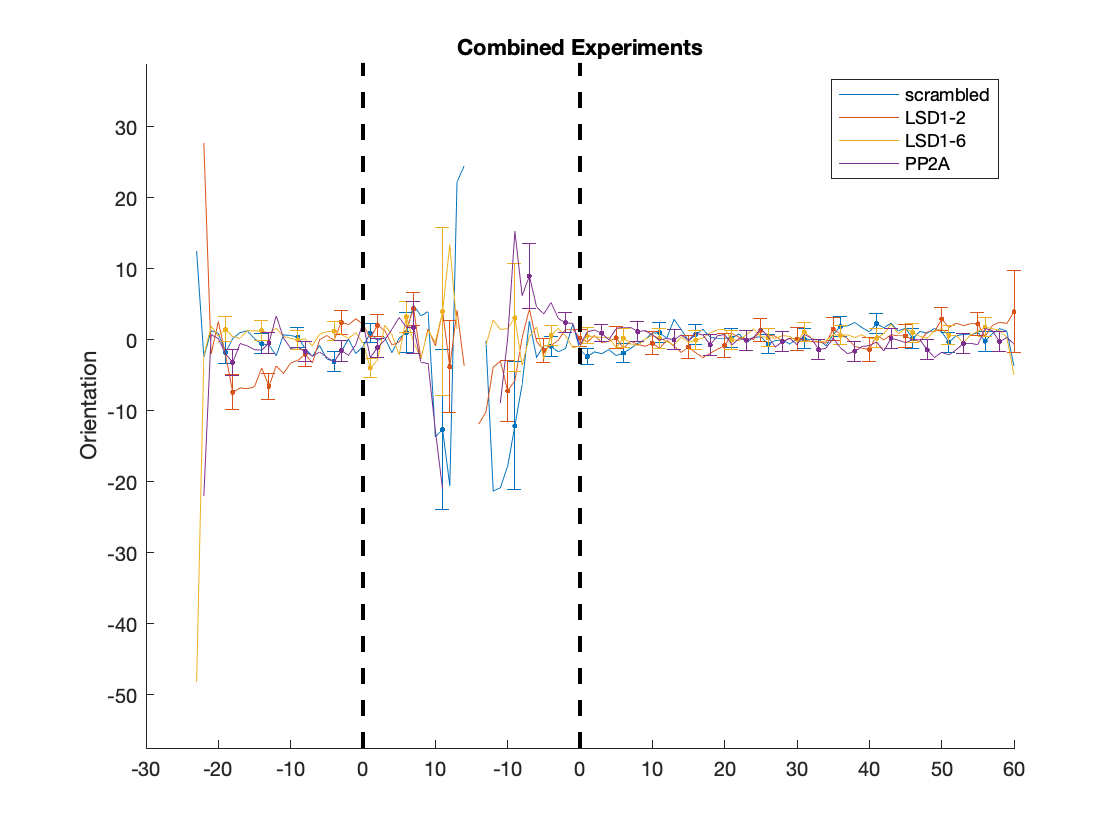

Supplement: S1 File — All existing single features and time series features are contained and accessible from an HTML-based overview file. Extract the archive to a folder of your choice and open the HTML file in the root directory using any web browser. (ZIP) [file pone.0270923.s022.zip › Plots/LSD1_FusedProjects_CARSync_AdditionalFeatures_Orientation_LinePlots.png]

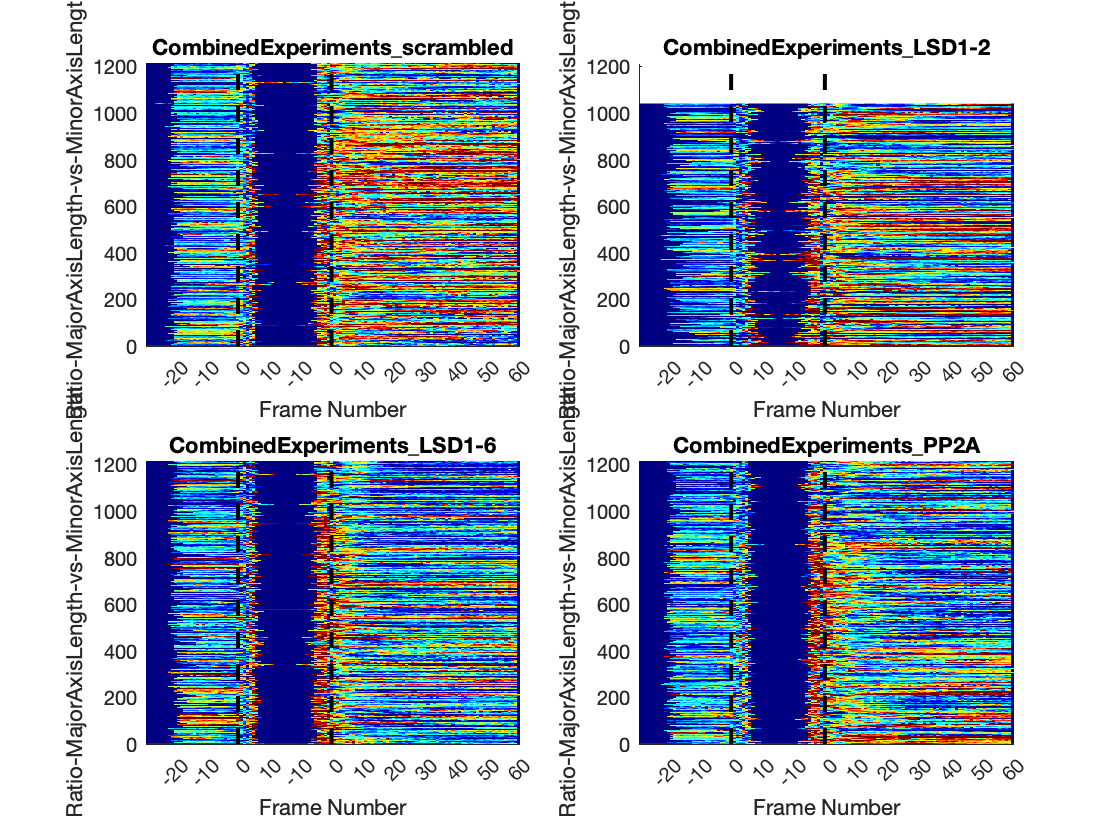

Supplement: S1 File — All existing single features and time series features are contained and accessible from an HTML-based overview file. Extract the archive to a folder of your choice and open the HTML file in the root directory using any web browser. (ZIP) [file pone.0270923.s022.zip › Plots/LSD1_FusedProjects_CARSync_AdditionalFeatures_Ratio-MajorAxisLength-vs-MinorAxisLength_HeatMaps.png]

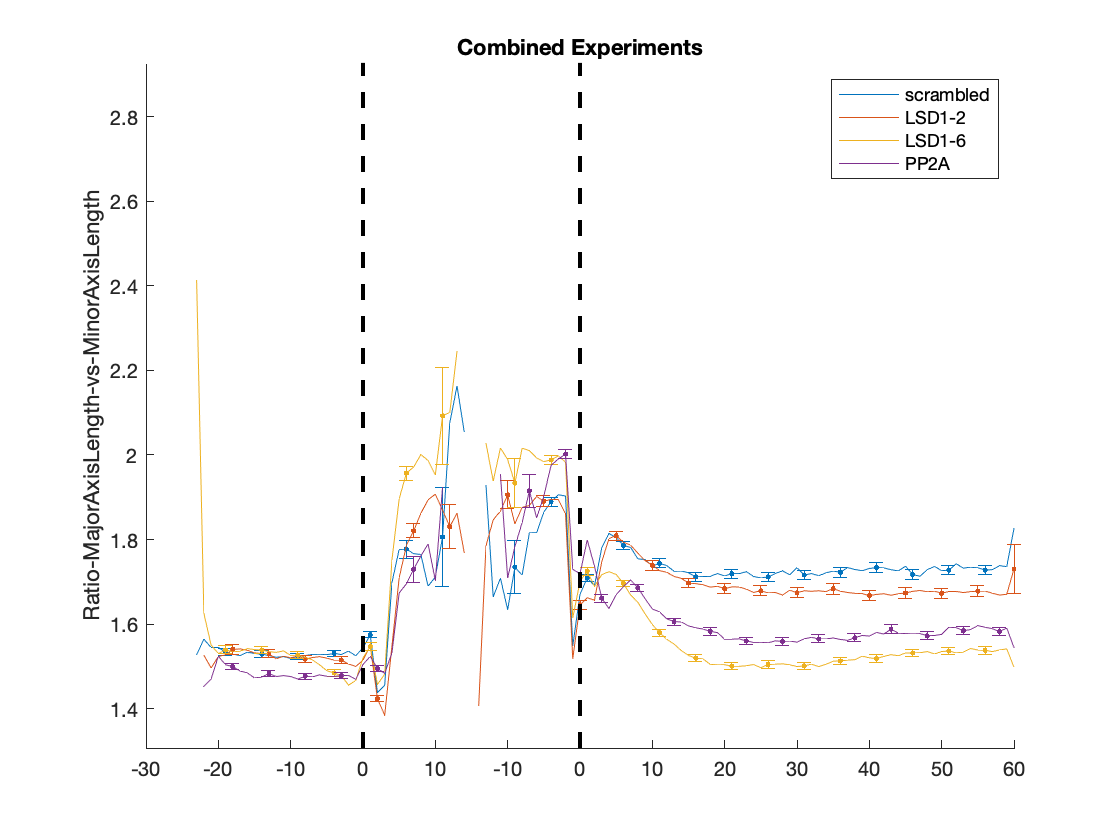

Supplement: S1 File — All existing single features and time series features are contained and accessible from an HTML-based overview file. Extract the archive to a folder of your choice and open the HTML file in the root directory using any web browser. (ZIP) [file pone.0270923.s022.zip › Plots/LSD1_FusedProjects_CARSync_AdditionalFeatures_Ratio-MajorAxisLength-vs-MinorAxisLength_LinePlots.png]

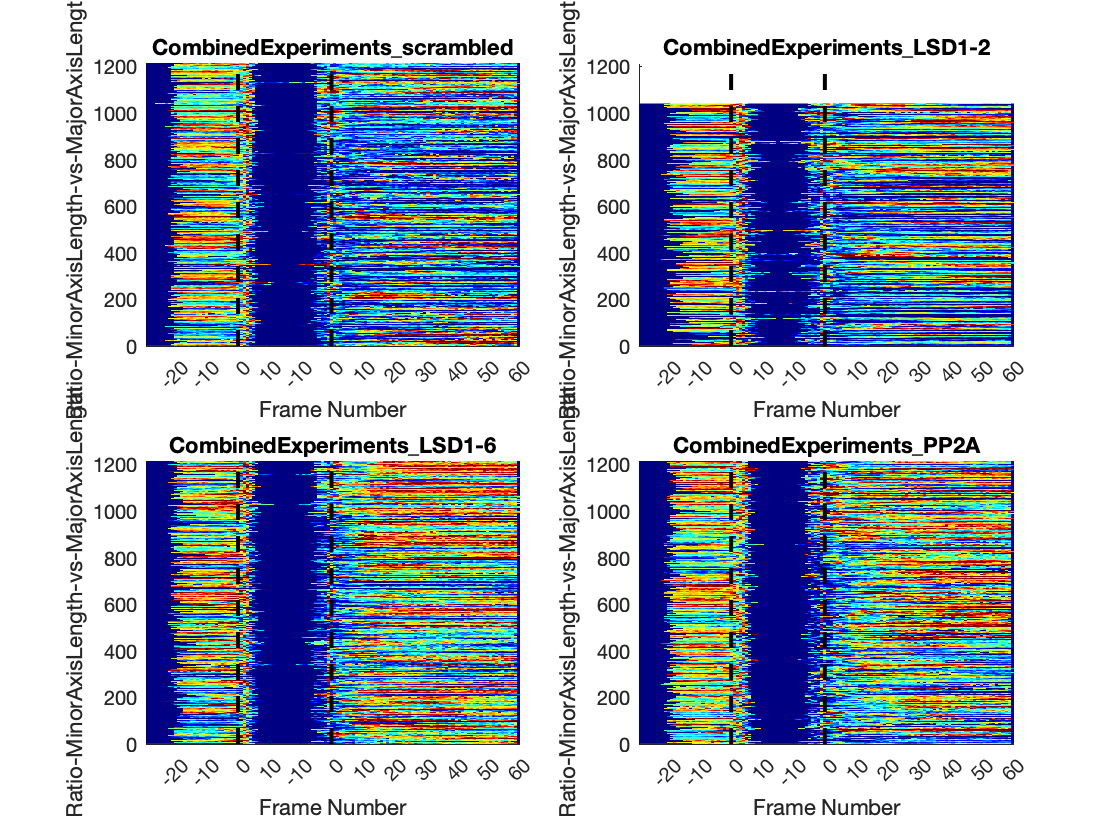

Supplement: S1 File — All existing single features and time series features are contained and accessible from an HTML-based overview file. Extract the archive to a folder of your choice and open the HTML file in the root directory using any web browser. (ZIP) [file pone.0270923.s022.zip › Plots/LSD1_FusedProjects_CARSync_AdditionalFeatures_Ratio-MinorAxisLength-vs-MajorAxisLength_HeatMaps.png]

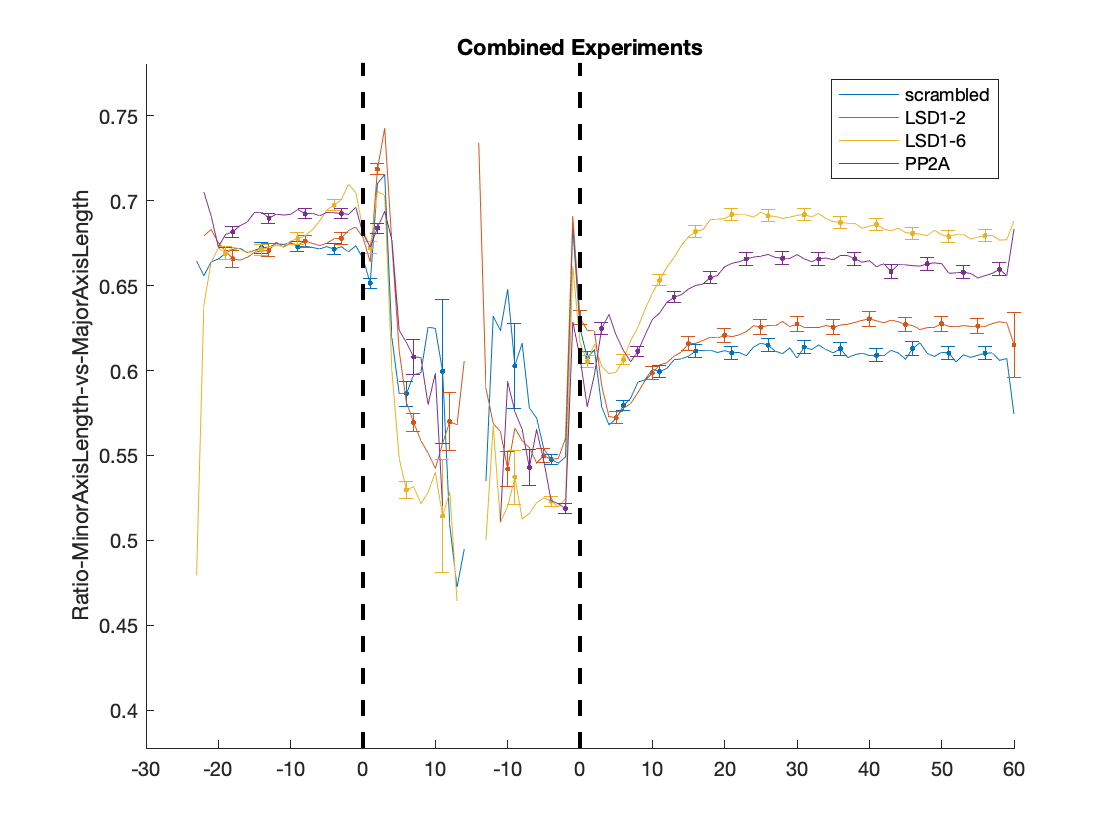

Supplement: S1 File — All existing single features and time series features are contained and accessible from an HTML-based overview file. Extract the archive to a folder of your choice and open the HTML file in the root directory using any web browser. (ZIP) [file pone.0270923.s022.zip › Plots/LSD1_FusedProjects_CARSync_AdditionalFeatures_Ratio-MinorAxisLength-vs-MajorAxisLength_LinePlots.png]

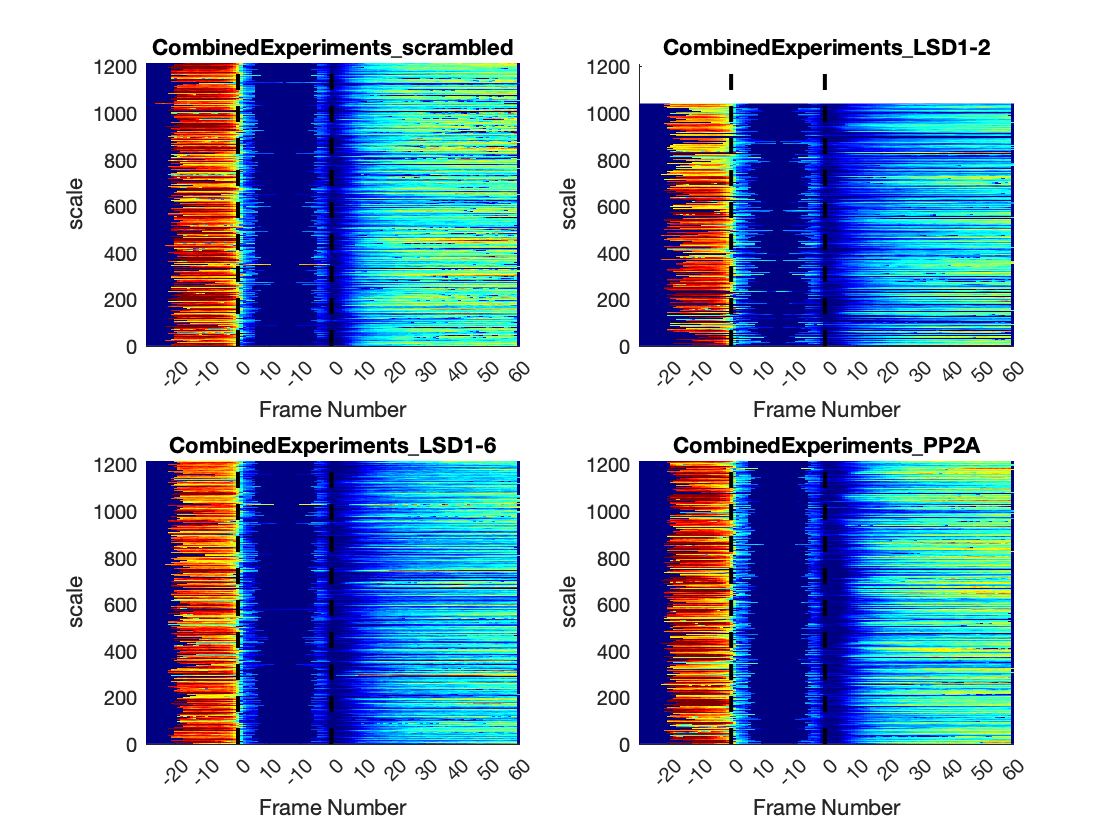

Supplement: S1 File — All existing single features and time series features are contained and accessible from an HTML-based overview file. Extract the archive to a folder of your choice and open the HTML file in the root directory using any web browser. (ZIP) [file pone.0270923.s022.zip › Plots/LSD1_FusedProjects_CARSync_AdditionalFeatures_scale_HeatMaps.png]

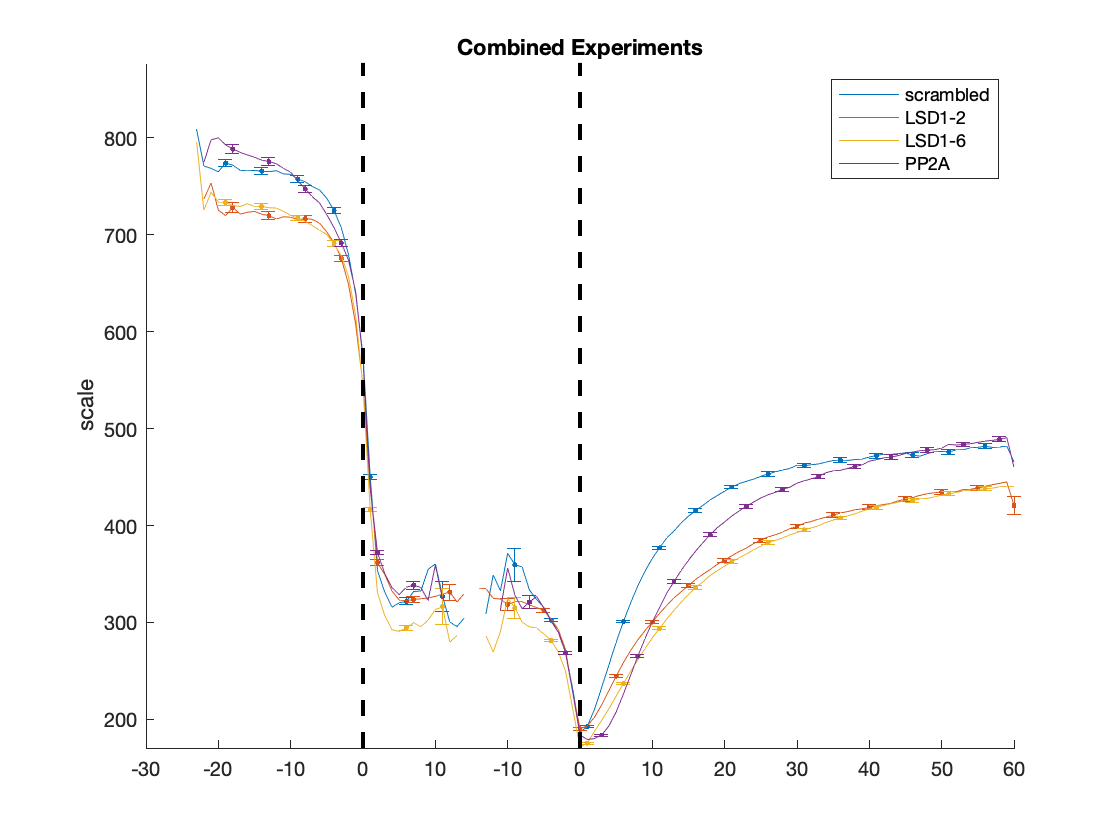

Supplement: S1 File — All existing single features and time series features are contained and accessible from an HTML-based overview file. Extract the archive to a folder of your choice and open the HTML file in the root directory using any web browser. (ZIP) [file pone.0270923.s022.zip › Plots/LSD1_FusedProjects_CARSync_AdditionalFeatures_scale_LinePlots.png]

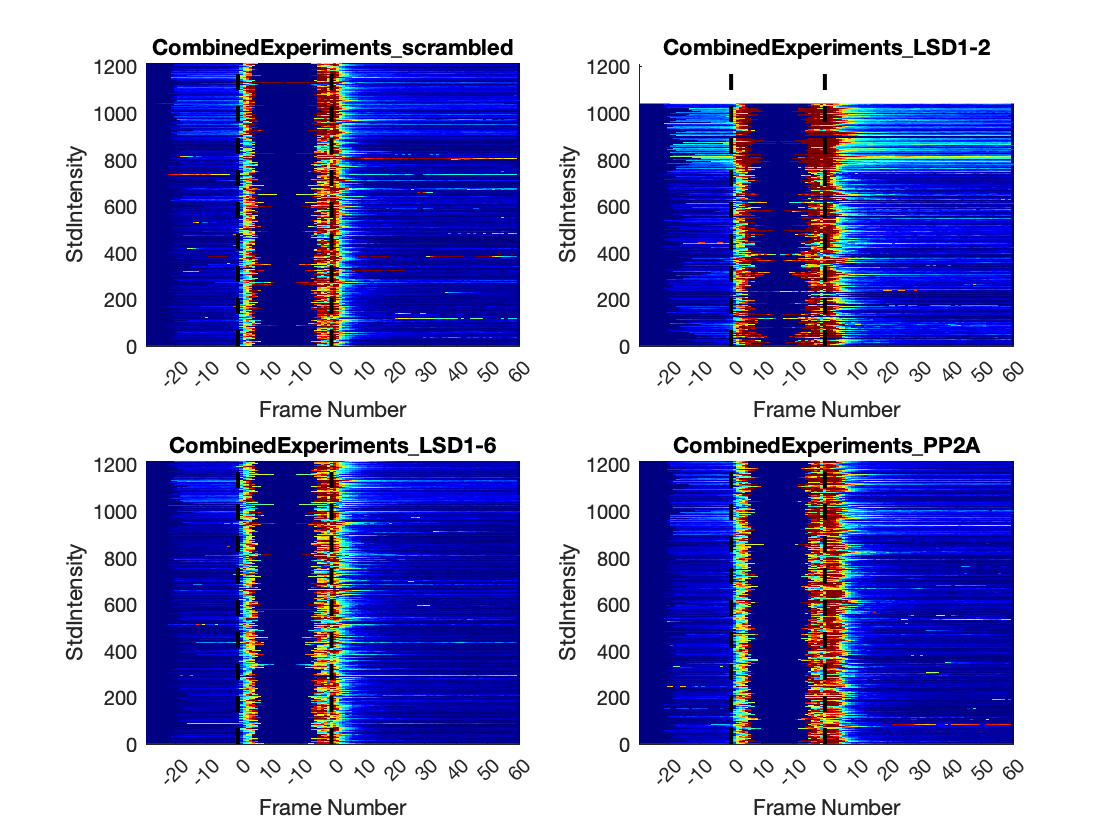

Supplement: S1 File — All existing single features and time series features are contained and accessible from an HTML-based overview file. Extract the archive to a folder of your choice and open the HTML file in the root directory using any web browser. (ZIP) [file pone.0270923.s022.zip › Plots/LSD1_FusedProjects_CARSync_AdditionalFeatures_StdIntensity_HeatMaps.png]

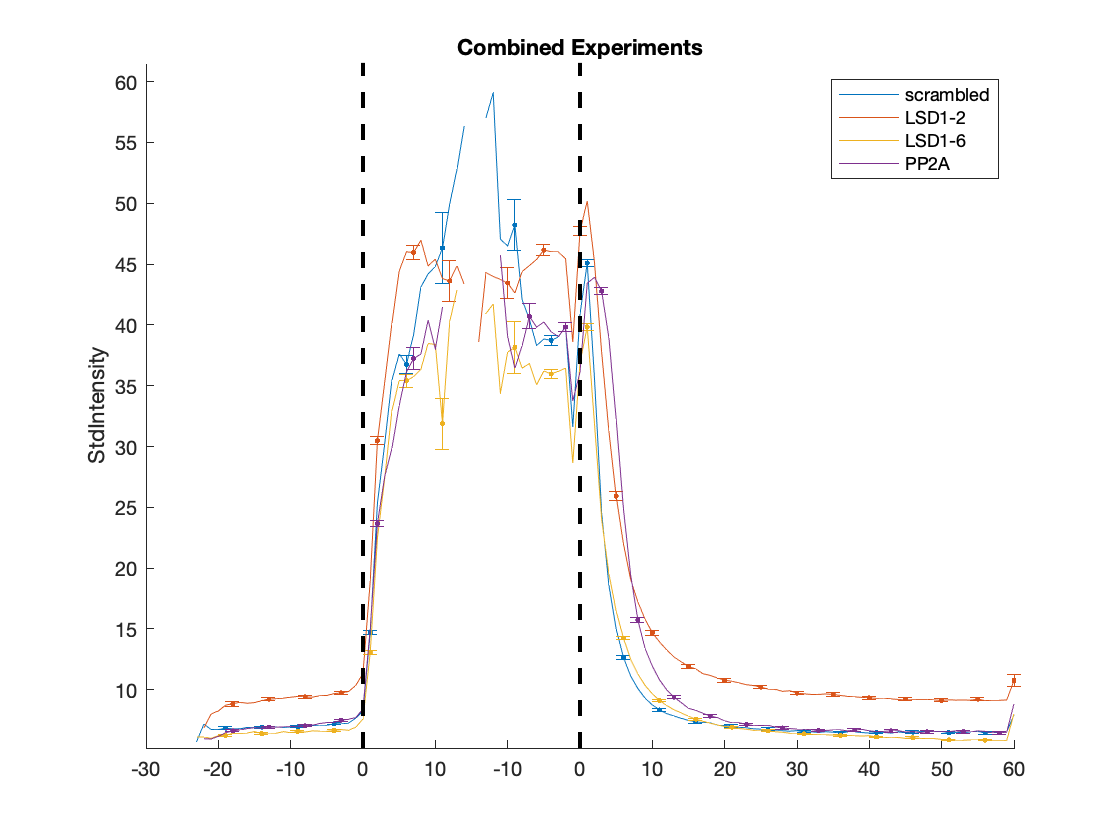

Supplement: S1 File — All existing single features and time series features are contained and accessible from an HTML-based overview file. Extract the archive to a folder of your choice and open the HTML file in the root directory using any web browser. (ZIP) [file pone.0270923.s022.zip › Plots/LSD1_FusedProjects_CARSync_AdditionalFeatures_StdIntensity_LinePlots.png]

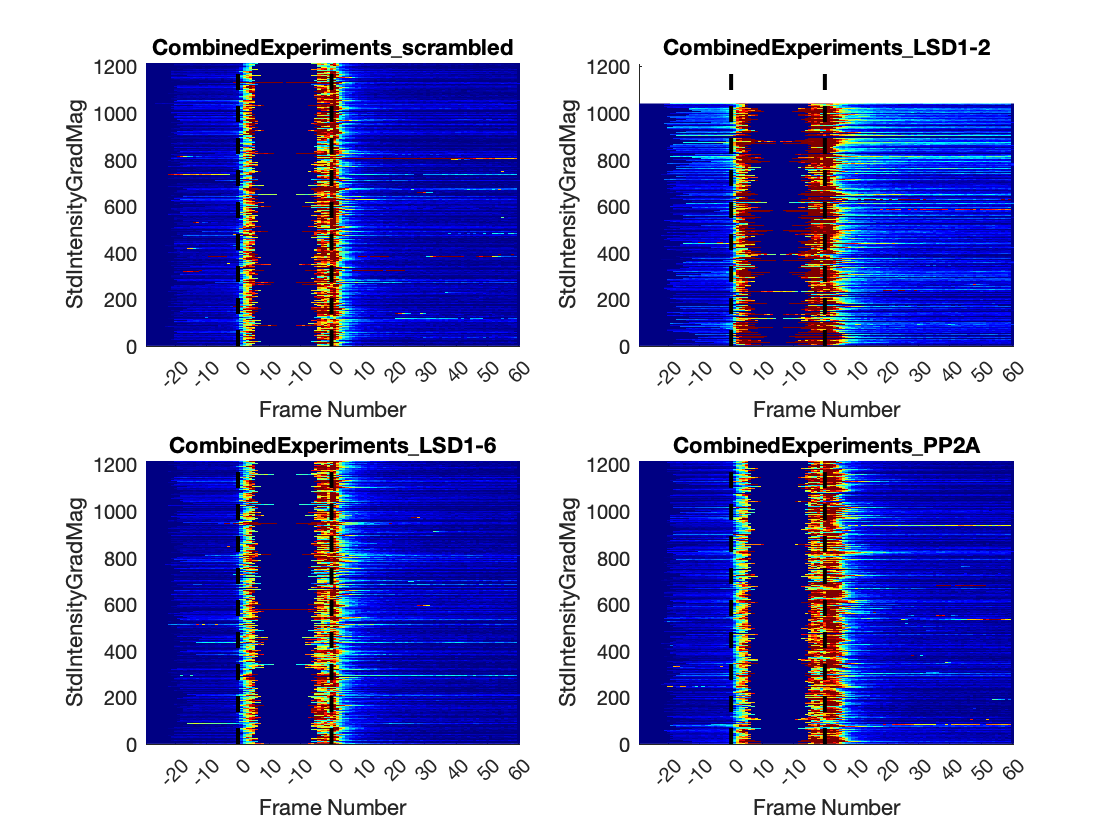

Supplement: S1 File — All existing single features and time series features are contained and accessible from an HTML-based overview file. Extract the archive to a folder of your choice and open the HTML file in the root directory using any web browser. (ZIP) [file pone.0270923.s022.zip › Plots/LSD1_FusedProjects_CARSync_AdditionalFeatures_StdIntensityGradMag_HeatMaps.png]

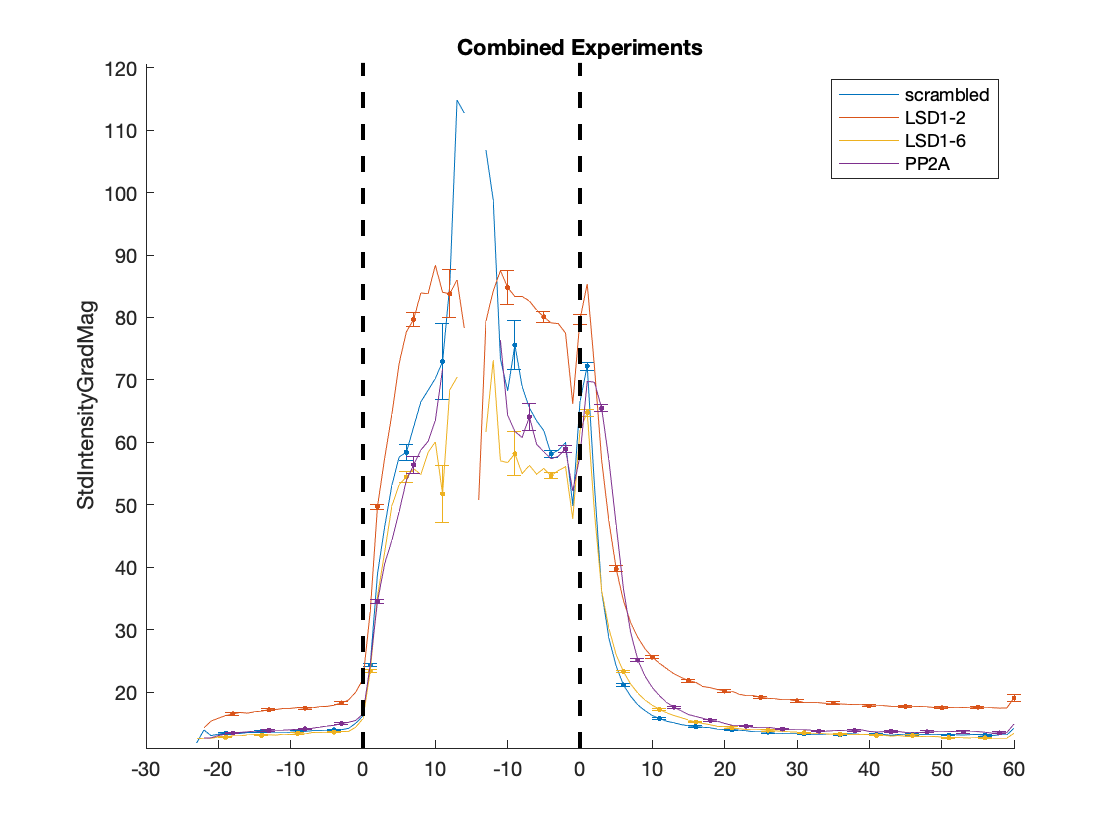

Supplement: S1 File — All existing single features and time series features are contained and accessible from an HTML-based overview file. Extract the archive to a folder of your choice and open the HTML file in the root directory using any web browser. (ZIP) [file pone.0270923.s022.zip › Plots/LSD1_FusedProjects_CARSync_AdditionalFeatures_StdIntensityGradMag_LinePlots.png]

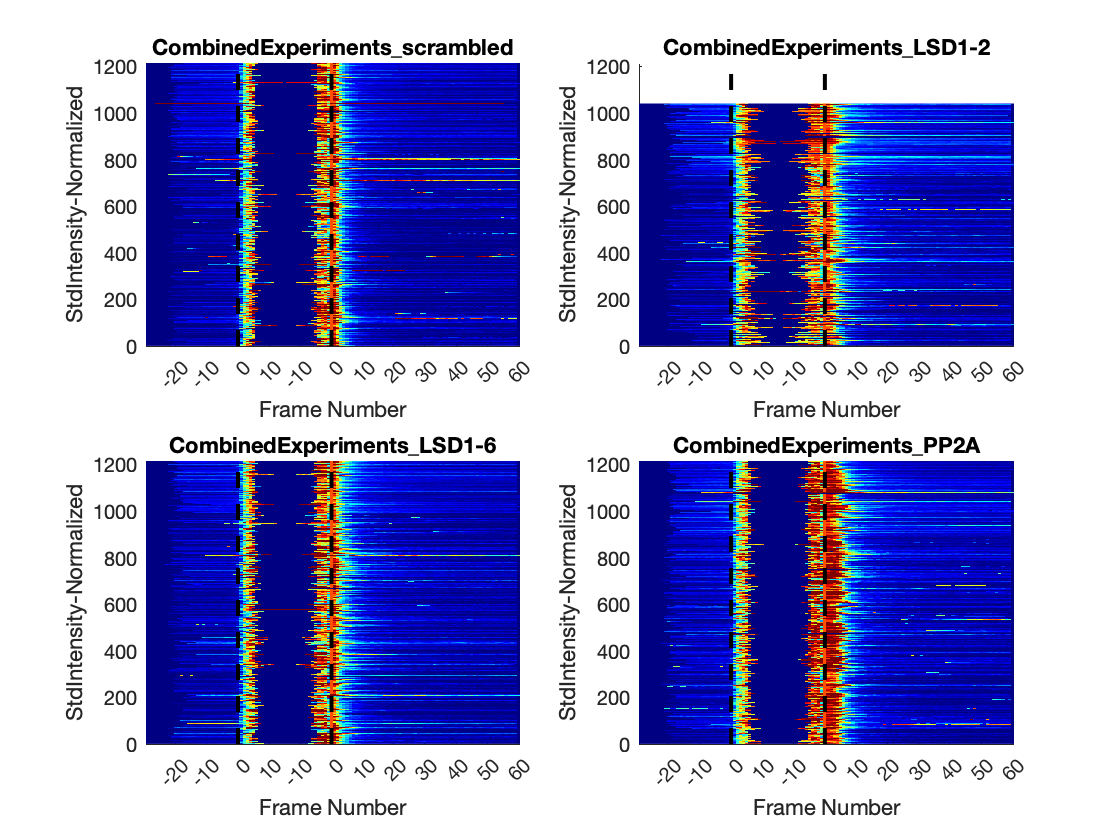

Supplement: S1 File — All existing single features and time series features are contained and accessible from an HTML-based overview file. Extract the archive to a folder of your choice and open the HTML file in the root directory using any web browser. (ZIP) [file pone.0270923.s022.zip › Plots/LSD1_FusedProjects_CARSync_AdditionalFeatures_StdIntensity-Normalized_HeatMaps.png]

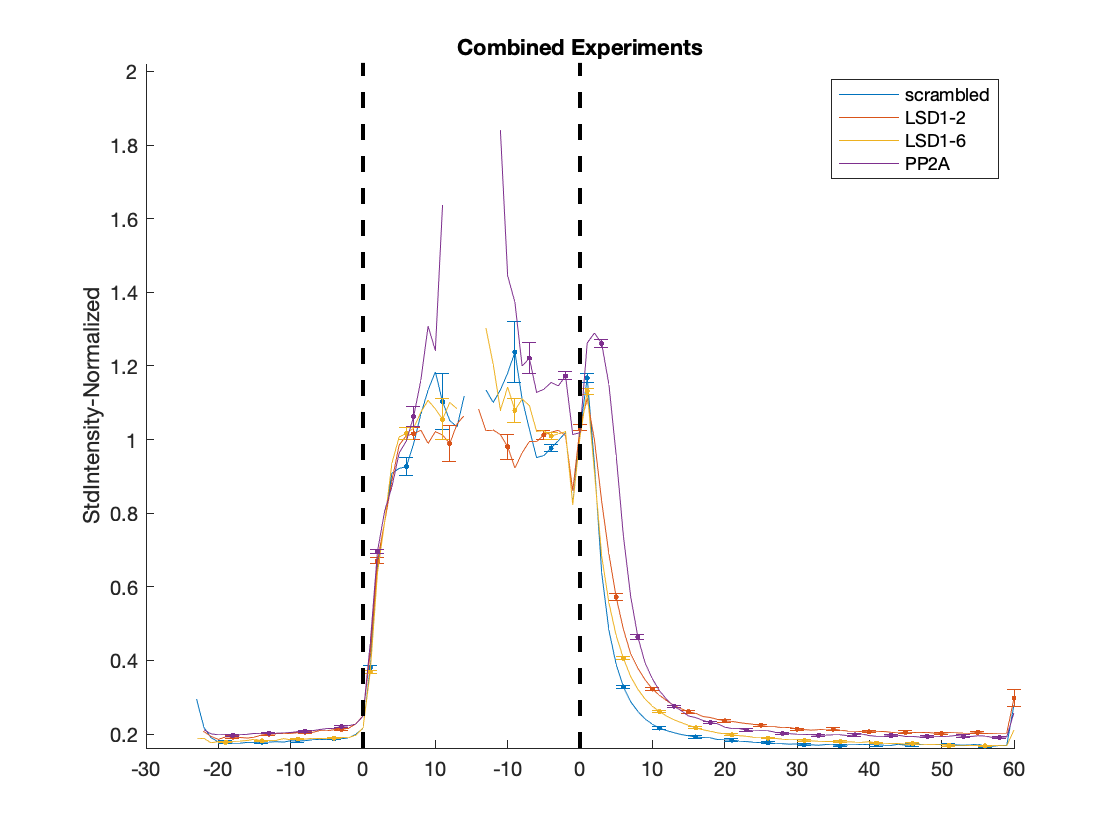

Supplement: S1 File — All existing single features and time series features are contained and accessible from an HTML-based overview file. Extract the archive to a folder of your choice and open the HTML file in the root directory using any web browser. (ZIP) [file pone.0270923.s022.zip › Plots/LSD1_FusedProjects_CARSync_AdditionalFeatures_StdIntensity-Normalized_LinePlots.png]

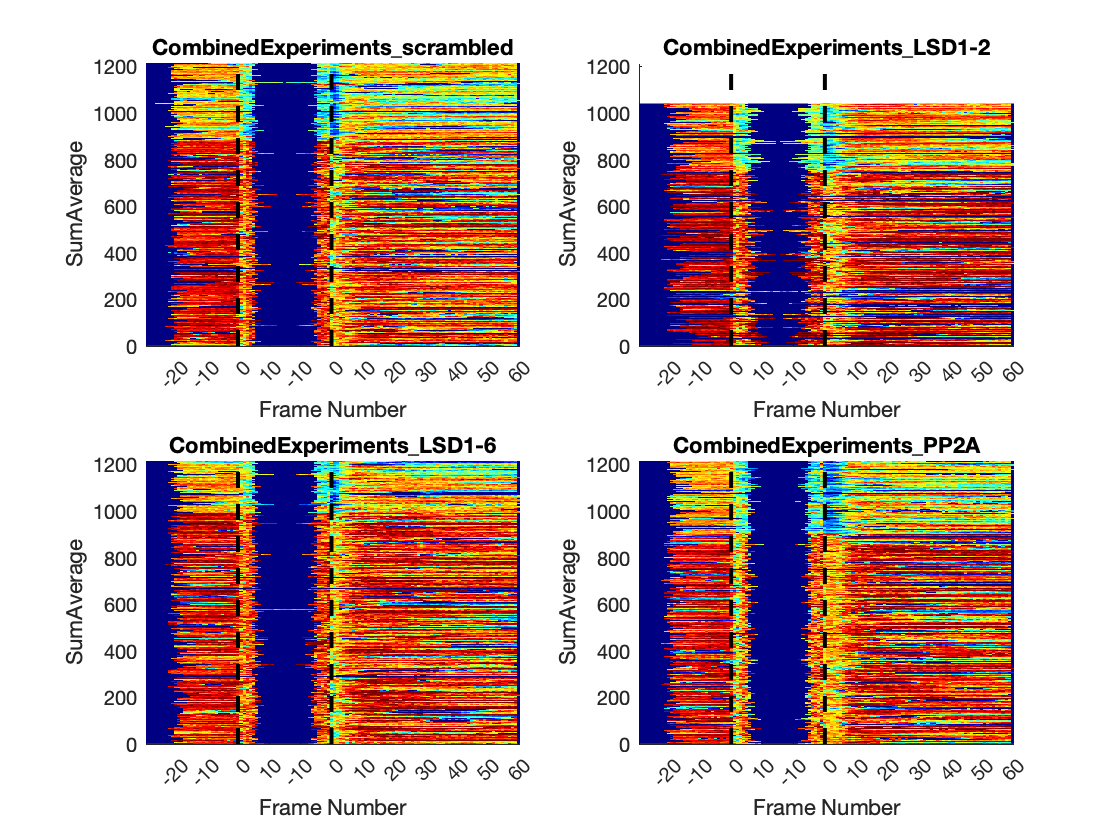

Supplement: S1 File — All existing single features and time series features are contained and accessible from an HTML-based overview file. Extract the archive to a folder of your choice and open the HTML file in the root directory using any web browser. (ZIP) [file pone.0270923.s022.zip › Plots/LSD1_FusedProjects_CARSync_AdditionalFeatures_SumAverage_HeatMaps.png]

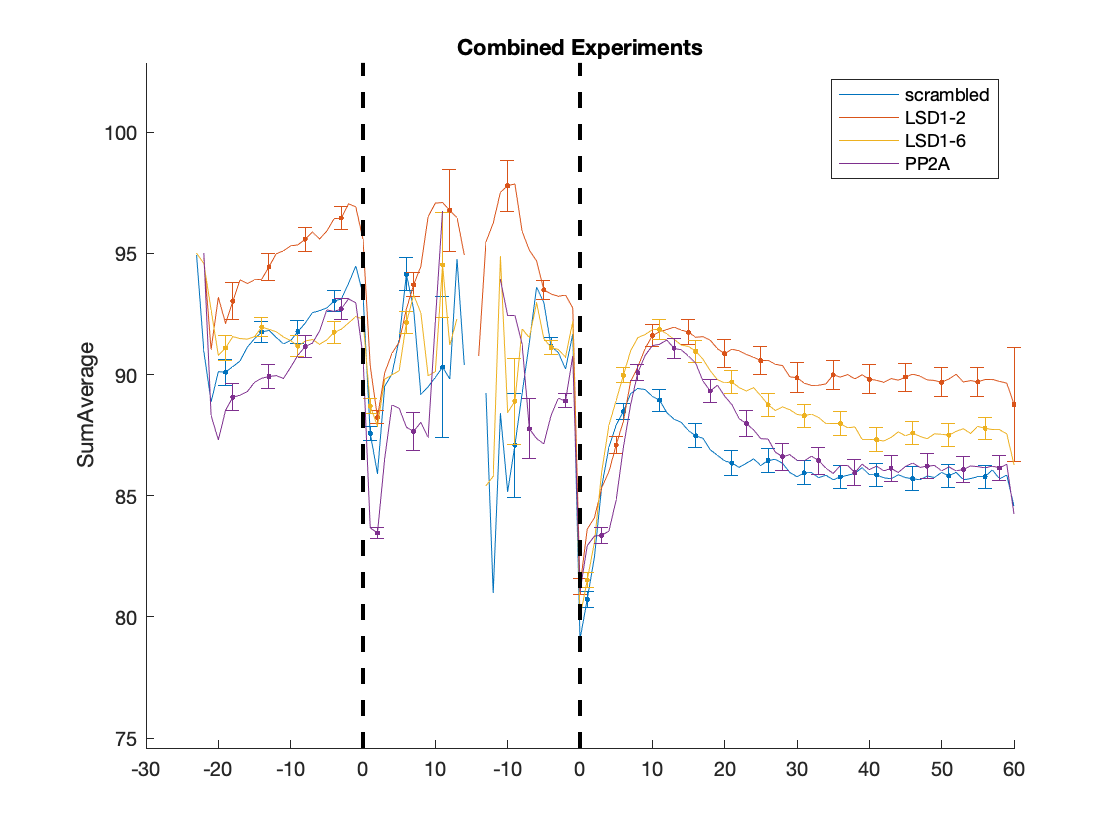

Supplement: S1 File — All existing single features and time series features are contained and accessible from an HTML-based overview file. Extract the archive to a folder of your choice and open the HTML file in the root directory using any web browser. (ZIP) [file pone.0270923.s022.zip › Plots/LSD1_FusedProjects_CARSync_AdditionalFeatures_SumAverage_LinePlots.png]

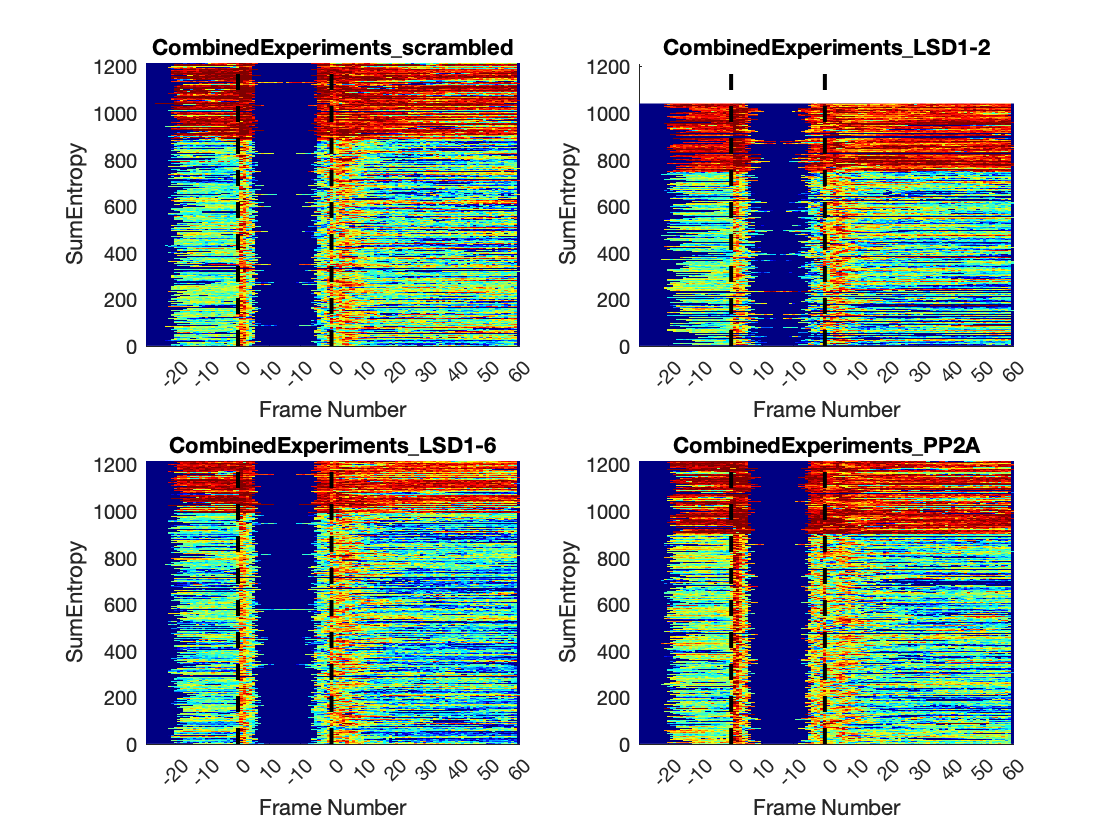

Supplement: S1 File — All existing single features and time series features are contained and accessible from an HTML-based overview file. Extract the archive to a folder of your choice and open the HTML file in the root directory using any web browser. (ZIP) [file pone.0270923.s022.zip › Plots/LSD1_FusedProjects_CARSync_AdditionalFeatures_SumEntropy_HeatMaps.png]

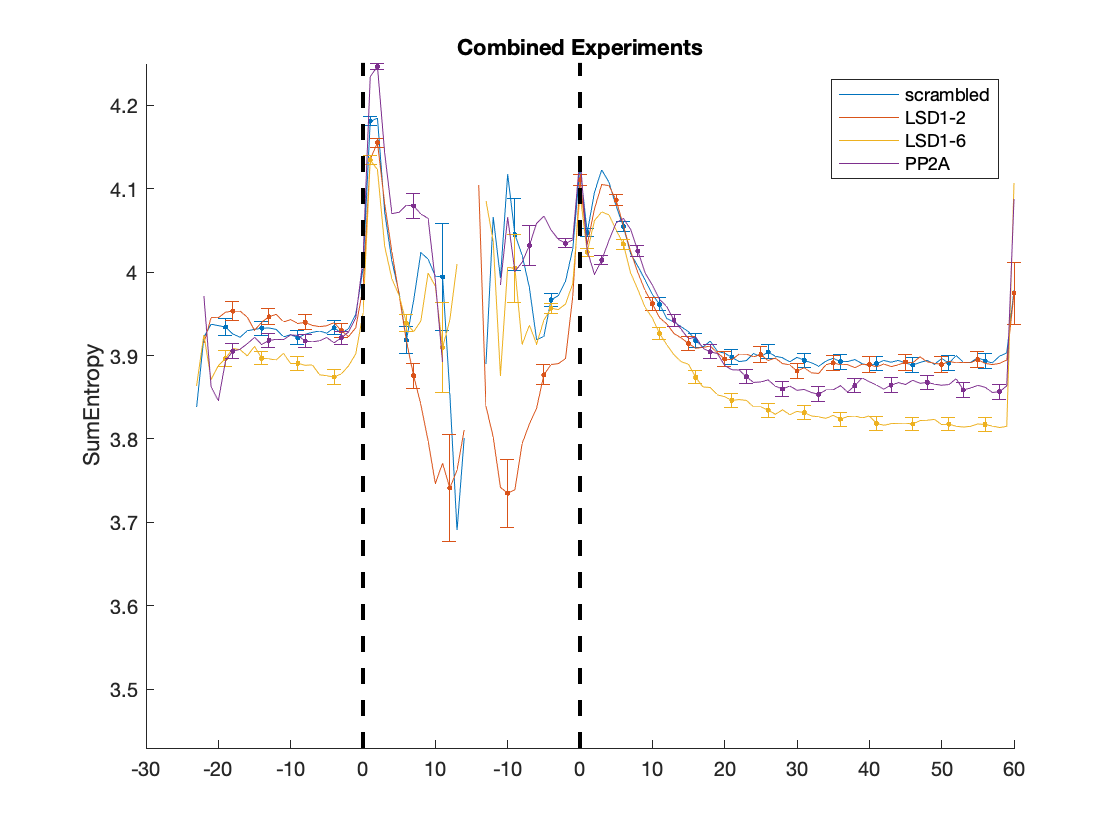

Supplement: S1 File — All existing single features and time series features are contained and accessible from an HTML-based overview file. Extract the archive to a folder of your choice and open the HTML file in the root directory using any web browser. (ZIP) [file pone.0270923.s022.zip › Plots/LSD1_FusedProjects_CARSync_AdditionalFeatures_SumEntropy_LinePlots.png]

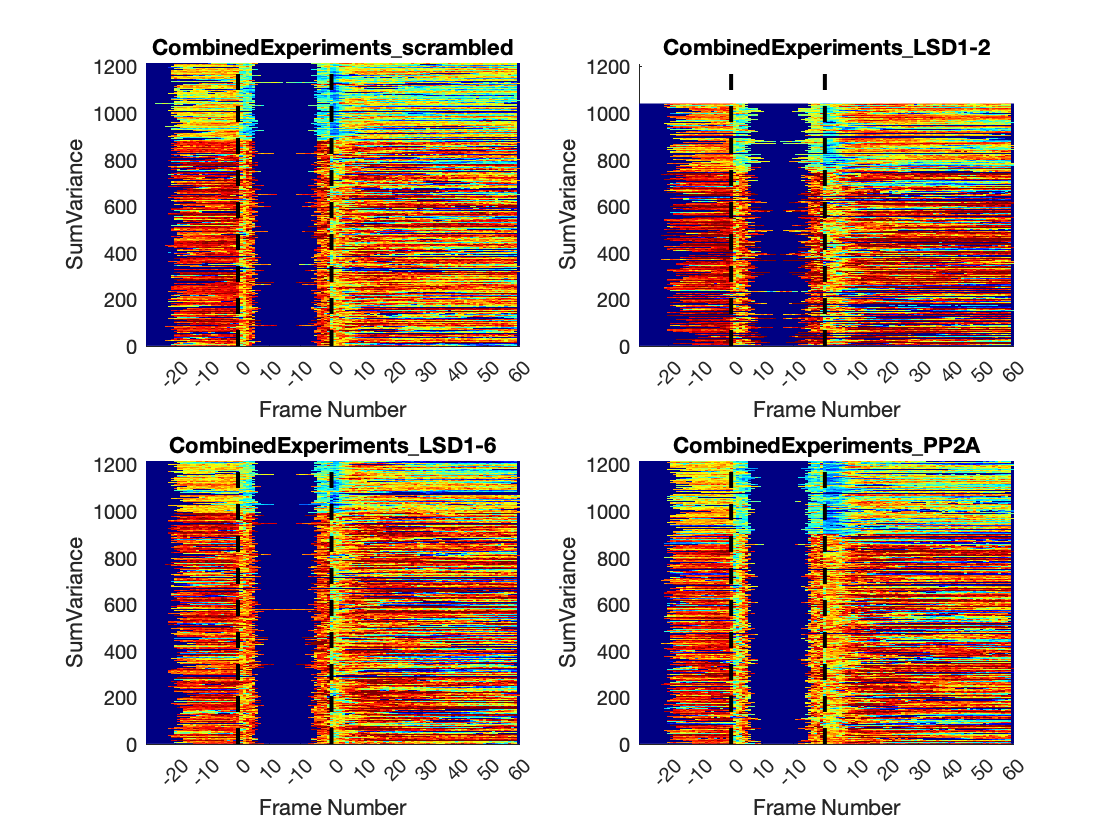

Supplement: S1 File — All existing single features and time series features are contained and accessible from an HTML-based overview file. Extract the archive to a folder of your choice and open the HTML file in the root directory using any web browser. (ZIP) [file pone.0270923.s022.zip › Plots/LSD1_FusedProjects_CARSync_AdditionalFeatures_SumVariance_HeatMaps.png]

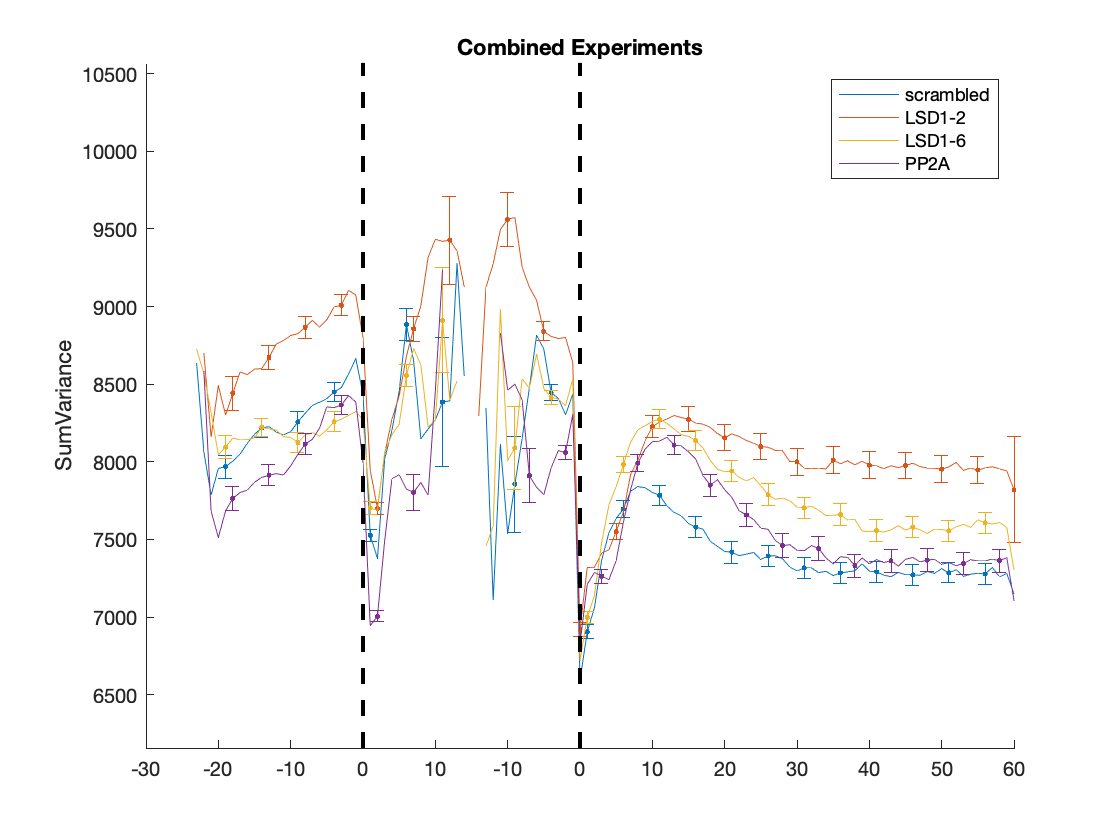

Supplement: S1 File — All existing single features and time series features are contained and accessible from an HTML-based overview file. Extract the archive to a folder of your choice and open the HTML file in the root directory using any web browser. (ZIP) [file pone.0270923.s022.zip › Plots/LSD1_FusedProjects_CARSync_AdditionalFeatures_SumVariance_LinePlots.png]

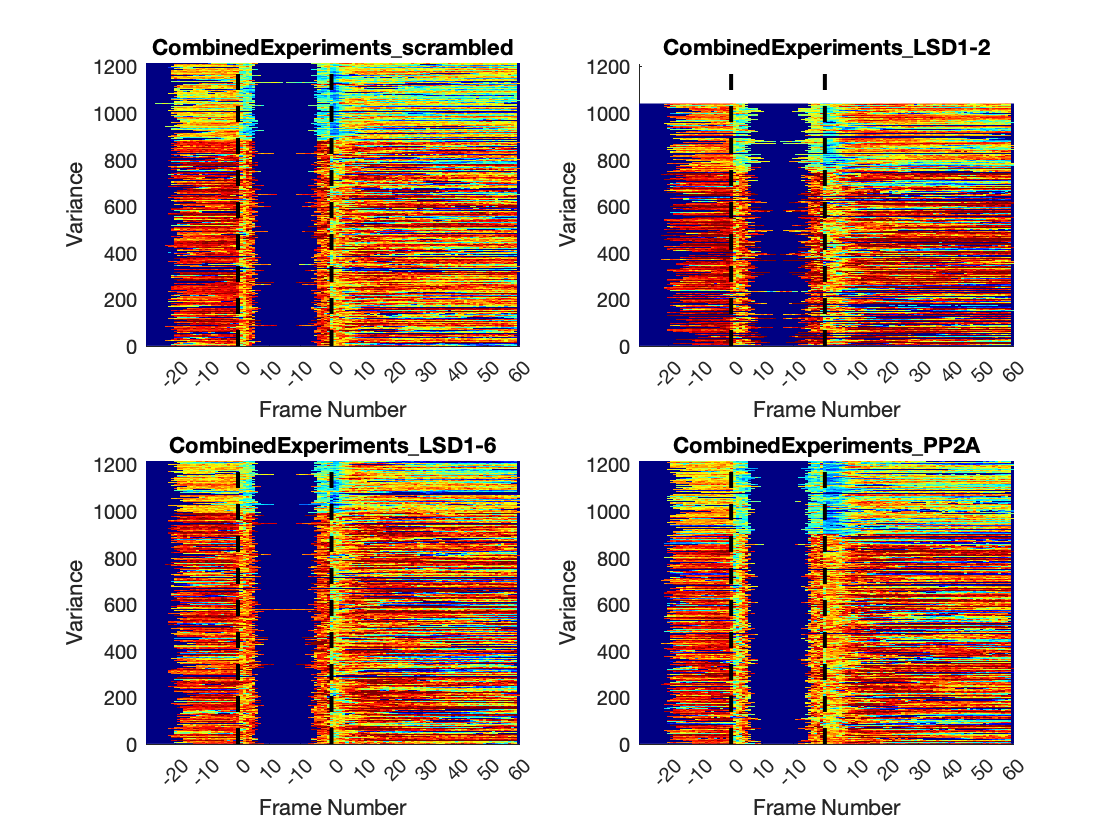

Supplement: S1 File — All existing single features and time series features are contained and accessible from an HTML-based overview file. Extract the archive to a folder of your choice and open the HTML file in the root directory using any web browser. (ZIP) [file pone.0270923.s022.zip › Plots/LSD1_FusedProjects_CARSync_AdditionalFeatures_Variance_HeatMaps.png]

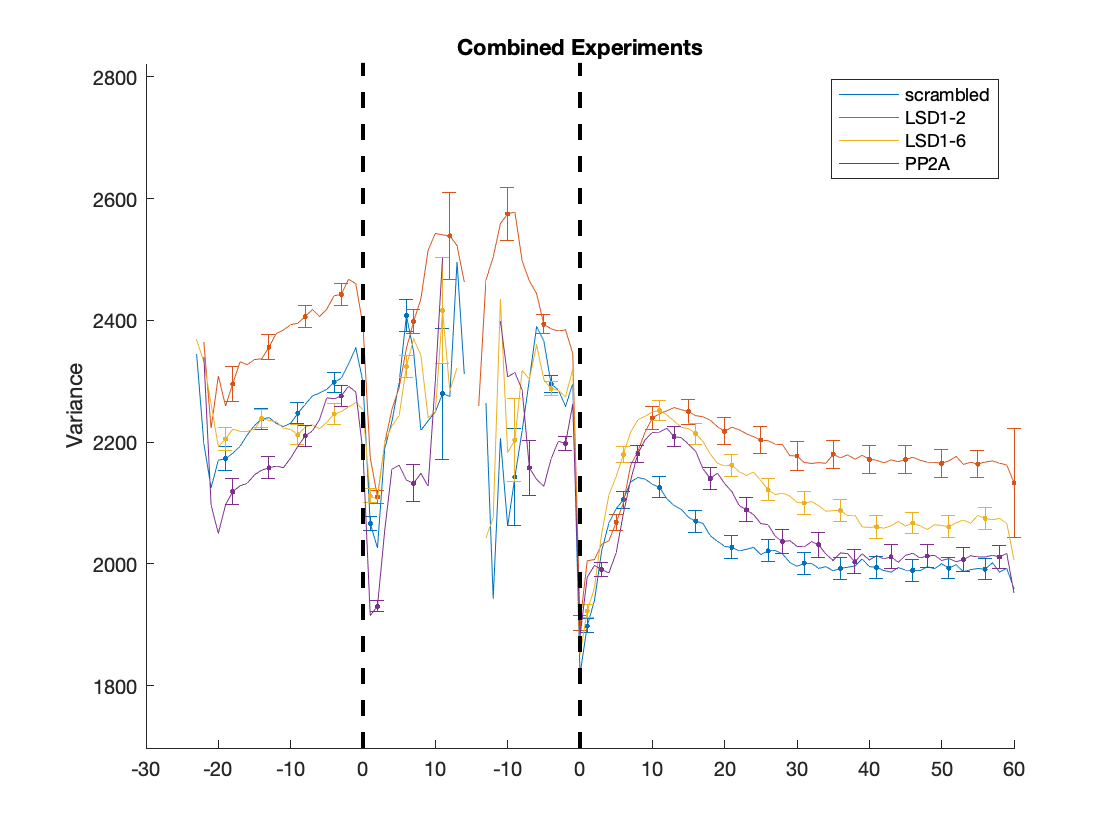

Supplement: S1 File — All existing single features and time series features are contained and accessible from an HTML-based overview file. Extract the archive to a folder of your choice and open the HTML file in the root directory using any web browser. (ZIP) [file pone.0270923.s022.zip › Plots/LSD1_FusedProjects_CARSync_AdditionalFeatures_Variance_LinePlots.png]

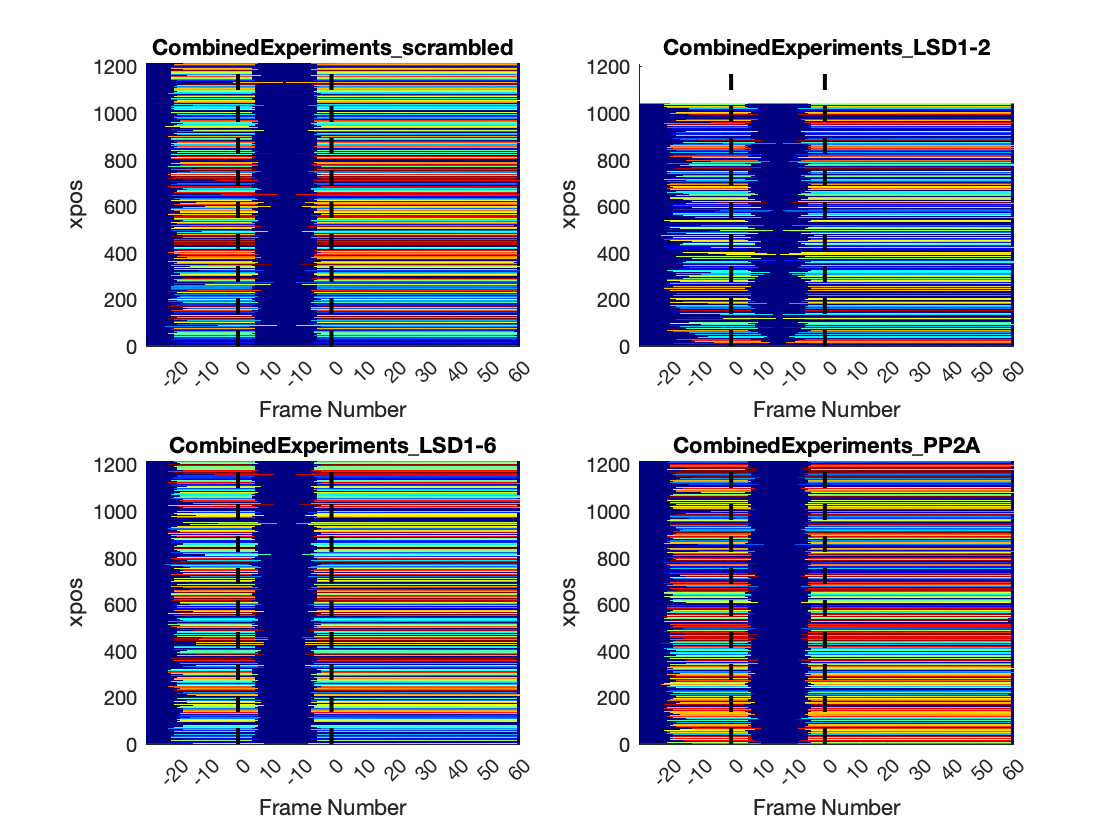

Supplement: S1 File — All existing single features and time series features are contained and accessible from an HTML-based overview file. Extract the archive to a folder of your choice and open the HTML file in the root directory using any web browser. (ZIP) [file pone.0270923.s022.zip › Plots/LSD1_FusedProjects_CARSync_AdditionalFeatures_xpos_HeatMaps.png]
