# Supplementary material for: LiveCellMiner: A new tool to analyze mitotic progression
Source: PLoS One. 2022 Jul 7;17(7):e0270923. doi: 10.1371/journal.pone.0270923 (PMC9262191; doi:10.1371/journal.pone.0270923)
Supplement: Attachment — Submitted filename: ResponseToReviewers.pdf [file pone.0270923.s024.pdf]

We thank all the reviewers for their valuable feedback on our manuscript. Please find the point-by-point response to all raised concerns below:

Reviewer #1:

In this manuscript, the authors describe a new analysis tool called LiveCellMiner that enables segmentation, tracking and analysis of fluorescence microscopy images (2D+t). They use both previously published and new datasets to illustrate the power of LiveCellMiner to extract useful information regarding mitotic phenotypes. Most impressively, LiveCellMiner can provide valuable insight into nuclear morphology, chromatin rotation and mitotic progress (prophase to metaphase as well as through anaphase onset to completion) without an apparent need for training on experiment-specific classifiers. In addition, it appears to perform well across different platforms. The authors primarily focus on three proteins and their previously described mitotic roles; they found that LiveCellMiner could corroborate published results on PP2A, LSD1 or RecQL4, while providing unexpected new insights in terms of nuclear morphologies and spindle rotations. In addition, the authors suggest that LiveCellMiner can perform well on high content datasets; here they provide visual outputs for data obtained from their own published experiments and demonstrate defects in nuclear morphology and mitotic progression for 5 genes.

Overall, LiveCellMiner seems to be a very useful tool to analyse morphological changes in the chromatin over time in live cell imaging experiments. As such it has the potential to be very useful to the research community.

Specific points:

1. The authors should clearly state the cell type(s) used in each figure of the study. My impression is that all the data are derived from HeLa cells but I may be wrong. If indeed LiveCellMiner was tested only on HeLa cells, it would be vital that it is also tried on other cell types such as diploid RPE1 or fibroblasts. Whereas HeLa cells are fairly straightforward to segment, track and image in time-lapse experiments, and show clearly distinguishable features, this is not necessarily the case for other cell types. For instance, some cell types move about a lot more and undergo massive morphological changes while doing so. Furthermore, there could be cases where the 3 mins frame frequency is unattainable (i.e. when imaging for 3-4 days, photo/laser toxicity may require frames of 5-8 mins) and therefore it would be important to see how LiveCellMiner performs in segmenting and tracking cells in such image sequences. Ultimately, for LiveCellMiner to fulfil its promise, it needs to work across a range of cell types and shapes. The authors could try LiveCellMiner on published datasets obtained from other cell types or generate their own data by treating various cell lines with established mitotic inhibitors (easier and simpler than siRNA).

We appreciate this suggestion, which was in fact planned for subsequent work. To provide evidence of LiveCellMiner's robustness and versatility with cell lines less amenable to segmentation and tracking than HeLa cells, we include the analysis of PP2A down-regulation in RPE cells as a supplementary figure (S9 Fig). In addition, following the reviewer's suggestions, we challenged LiveCellMiner with image sequences recorded every 8.5 min. For this, we have used an already published data set (Hériché *et al.*, 2014). Figure S10 shows the mitotic features of factors studied in that work (CDC20 and Aurora Kinase A (AurKA)). A comparison of the image quality and temporal resolution is provided in the newly added Figure S12.

2. I am confused about the part on the utility of LiveCellMiner to analyse data from high content screening. For these experiments the authors reference their own recent publication in Cells 2020, which does not include a high content screen. Fig S4 shows data for 7 siRNAs (2 scrambled and 5 targeting VPS72, PP2A, EP400, SRCAP and Ino80). Do the authors use HSC to refer to an HSC platform? This should be clearly stated in the manuscript.

In line with the previous comment, we reanalyzed several positions from a high-content screen previously published by Hériché et al., 2014 and the following paragraph was added:

“The concept of high-content screening implies that thousands of targets are tested in parallel. Often these setups require imaging for more than three days and slower frame frequency (*i.e.*, 5

to 8 min), which is needed to avoid phototoxicity and to provide enough time for the imaging loop through hundreds of positions. Longer times between frames might negatively impact segmentation and tracking performance resulting in inefficient phenotype recognition. Thus, we sought to test how LiveCellMiner performs with very large data sets of live-cell image sequences. For this, we took advantage from an original data set where they screened for genes involved in chromosome condensation at the beginning of mitosis in HeLa cells recorded every 8.5 min. Constrained by our computing capacity, we reanalyzed only 76 positions of this HCS data set corresponding to solid-phase transfection of siRNA against scramble controls and two well-known mitotic regulators: CDC20 and Aurora Kinase A (AurKA). LiveCellMiner performed reasonably well in segmentation and tracking (S5 Fig., S4 Table}, S12 Fig.), accomplishing full analysis of each position in about 30 min with a single workstation. Processing time could be considerably reduced by data-parallel computing, *e.g.*, by distributing the positions of a screen to separate nodes of a computing cluster. In line with available knowledge, LiveCellMiner detects a delay in the early mitotic progression for siRNA mediated downregulation of CDC20 (Hein et al., 2014) and AurKA (Marumoto et al., 2003) (S10 Fig. C). Our analysis also reflects the known role of CDC20 during mitosis exit (Held et al., 2010) as a delay in chromatin expansion after anaphase onset in CDC20 downregulated cells (S10 Fig. A,B,D and E). These results, along with those in S9 Fig., demonstrate the ability of LiveCellMiner to successfully and rapidly analyze data, across a range of cell types and frame frequencies, including those from HCS."

3. LiveCellMiner is an extension of a previous MatLab-based tool SciXMiner (released by the same group) and uses a number of previously released/published applications. For application, it is the ultimate tool that matters, but for the publication, readers should be able to see the truly novel aspects of LiveCellMiner. I therefore recommend including a clear summary of the innovations included in LiveCellMiner.

LiveCellMiner indeed uses a few preexisting methods like XPIWIT, Cellpose and SciXMiner for object detection, segmentation and project format. We added the following statement:

"LiveCellMiner is an extension package for the general-purpose data mining MATLAB toolbox SciXMiner (Mikut et al., 2017) and makes use of some existing tools for object detection and segmentation (Bartschat et al., 2016; Stringer et al., 2021) (see S7 Table for a detailed list of dependencies)."

And added a new Table S7 that clarifies the dependency of LiveCellMiner to previously existing tools:

**Functionality**	**Software**
Object Detection	XPIWIT
Segmentation	Cellpose + LiveCellMiner
Tracking	LiveCellMiner
Project structure	SciXMiner
Graphical User Interface	SciXMiner + LiveCellMiner
Project processing and data import	LiveCellMiner
Project Fusion	LiveCellMiner
Automatic Synchronization	LiveCellMiner
Manual Synchronization GUI	LiveCellMiner
Data Selection	SciXMiner
Visualizations	LiveCellMiner + (SciXMiner)
Report Generation	LiveCellMiner
Dedicated Features	LiveCellMiner
Statistical Analysis	LiveCellMiner + (SciXMiner)

**Newly Developed Software**	
LiveCellMiner	Moreno-Andrés, D., Bhattacharyya, A., Scheufen, A., & Stegmaier, J. (2021). LiveCellMiner: A New Tool to Analyze Mitotic Progression. bioRxiv.
**Third Party Software**	
XPIWIT	Bartschat, A., Hübner, E., Reischl, M., Mikut, R., & Stegmaier, J. (2016). XPIWIT—an XML pipeline wrapper for the Insight Toolkit. Bioinformatics, 32(2), 315-317.
Cellpose	Stringer, C., Wang, T., Michaelos, M., & Pachitariu, M. (2021). Cellpose: a generalist algorithm for cellular segmentation. *Nature methods*, *18*(1), 100-106.
SciXMiner	Mikut, R., Bartschat, A., Doneit, W., Ordiano, J. Á. G., Schott, B., Stegmaier, J., ... & Reischl, M. (2017). The MATLAB toolbox SciXMiner: User's manual and programmer's guide. arXiv preprint arXiv:1704.03298.

4. The manuscript is overall well written but certain parts would benefit from simplification/clarification. For instance, I am not sure I understand what the authors mean by

"The generated phenotypic classes work as closed entities, where no gradual time-dependent changes can be accurately quantified."

Thanks for this suggestion. We reformulated the sentence and added some additional information: *"Phenotypic classifiers are extraordinarily useful for analyzing complex populations. But because classes are categorical entities, they cannot accurately quantify continuous time-dependent gradual changes, as usually assumed in existing tools (Held et al., 2010)."*
We have also modified some sentences for simplification (see PDF with highlighted changes for details on the smaller adaptions).

5. I have noted a number of typos across the manuscript and so authors may consider proofreading the final version. e.g. line 34 translational not transnational

Thanks for spotting this typo. It's corrected now and we also carefully went through the entire manuscript again to eradicate remaining typos.

Reviewer #2:

In their manuscript "LiveCellMiner: A New Tool to Analyze Mitotic Progress", the authors present a computational tool for the analysis of live cell imaging data with a particular focus on the quantification of mitotic progression and mitotic phenotypes. This tool contains methods for

segmentation, tracking, feature calculation, event detection, data visualization and in silico alignment. The authors apply this tool to RNAi experiments in HeLa cells stably expressing H2B-mCherry and eGFP-tubulin.

Automatic analysis of live cell imaging data with computer vision approaches to quantify and classify individual cells and their phenotypic dynamics is an important topic and interesting for an increasing number of researchers in biology. For this reason, computational tools to automatically analyze such data are important for the community.

The first part of the paper is related to the computational methods and the provided software tool (main focus of the paper) and the second part to the biological application. Given my background as a computational scientist, I will not comment on the relevance, novelty and impact of the latter.

The obvious problem related to the methodological work presented here, is that there is basically no novelty at all. Many of the methods are quite simple image analysis methods (Otsu thresholding, LoG, extracting basic features, tracking), other methods are state-of-the-art, but have been previously published (CellPose for nucleus segmentation, generic CNN-features). Most importantly, the overlap with the CellCognition paper (Held et al., 2010) regarding methods and software is quite striking (segmentation method, features, event selection, HMM, visualization and graphical output). There are also other tools for mining time-resolved phenotypes the authors should have compared their solution to (Antal et al. 2015). The novelty aspect therefore is clearly critical for this article.

We agree that LiveCellMiner is not a new method per se. However, we are convinced that the implementation of a well-selected set of methods to a user-friendly open-source software package dedicated for the analysis a variety of live cell screens related to mitotic progression is a novel contribution on its own. We are well-aware of the parallels to previously published CellCognition software and successfully used this valuable tool in our past research. However, we also learned its limitations with respect to manual classifier training per data set, the discrete number of classes, restrictions of the tracking algorithms and less flexibility for extensions. Ultimately, these limitations motivated the development of LiveCellMiner. We also acknowledge the availability of tools like the general-purpose visual analytics software presented in (Antal et al., 2015), but we do not think they would be applicable to our problem due to a lack of consideration of time-dependency of cell development and missing synchronization capabilities. We adapted the following paragraph in the main text for clarification and cited the proposed papers:

“Supervised and unsupervised phenotypic profiling of live-cell microscopy data is emerging as a powerful tool for clinicians, pharmaceutical industry and biology research in general. Multiparametric data analysis at single-cell level allows the integration of up to hundreds of quantitative features to describe and distinguish phenotypes going far beyond traditional approaches based on the analysis of one or a few features in single-cell images. Phenotypic classifiers are extraordinarily useful for analyzing complex populations. But because classes are categorical entities, they cannot accurately quantify continuous time-dependent gradual changes, as usually assumed in existing tools (Held et al., 2010). Moreover, in most of the cases where numerous features are measured and integrated to generate high-dimensional phenotypic profiles, it is unclear whether all of them are required for describing the phenotypic classes. However, the quantification of simple morphological and shape parameters, e.g., of the cell nucleus, has potential diagnostic value (Fischer et al., 2020, Katayama et al., 2021). In this regard, most available tools are able to measure health-related morphology profiles of tuneable complexity in static images of fixed cell samples (Way et al., 2021); for review, see Chandrasekaran et al., 2021). Moreover, general-purpose visual analytics tools like (Antal et al., 2015) allow a versatile analysis of HCS-derived features but are unsuitable for modeling temporal object dependencies and lack dedicated single-cell synchronization. Therefore, tools for unbiased and comprehensive analysis of image features directly reflecting time-dependent live-cell shape and morphology are urgently needed both in basic and translational research (Wang et al., 2020).”

Detailed comments:

1. Introduction (page 2): the limitations of existing software are not clearly explained.

See previous comment for the changes we incorporated to this paragraph for making the existing limitations more clear.

1.1 The authors mention that not all features that are extracted may be useful, but they do not really present a solution for this. Of note, interpretable features have been used before (Held et al., 2010).

Thanks for this comment, we agree that this information may be a bit hidden in the details. We do provide a solution for this by means of a feature ranking that can be exported as a project report in HTML format. This allows the user to quickly get a qualitative and quantitative overview of the present features and the ranking can be performed according to the n-fold change of each feature from the interphase average to the average of the first two prophase frames and the first two anaphase frames. Exemplary reports are also provided in S1 File and S2 File (can be viewed with a common internet browser). We adjusted the respective paragraph to make this more obvious as:

"Although it is good to have a variety of features that users can freely select from, not all features are necessarily relevant for characterizing a specific phenotype of interest. In addition to the targeted visualizations, LiveCellMiner thus also provides an easy way to obtain a ranked overview of all extracted features including statistical readouts like minimum, maximum, mean, standard deviation and median in table form as well as graphically using heat maps and combined line plots for all features. To identify which features are potentially suitable for characterizing phenotypic differences, we compute the n-fold change of each feature from the interphase average to the average of the first two prophase frames and the first two anaphase frames (window for averaging can be changed by the user). The generated report is exported in HTML format and can be conveniently displayed with any conventional internet browser (see File S1 and File S2 for a demonstration of automatically generated reports)."

1.2. The authors also state that subjectively chosen features might introduce bias. This is true, but on the other hand, users are of course (and should be) free to choose what they measure. In any case, the authors also do not address this point.

This is a valuable point and indeed the users are free, of course, to choose which features they want to analyze. LiveCellMiner just provides the list of extracted features and the possibility to add additional features like secondary channel information, feature changes across different mitotic phases and the like. Moreover, as mentioned in the previous comment 1.1, users can also quickly generate a report of the entire project to get a quantitative and qualitative impression of how the features behave during development. Based on extracted quantitative properties for each feature the user can (but is not obliged to) select the features of interest for the research question based on an objective feature ranking. The following sentence should hopefully clarify this:

„Although it is good to have a variety of features that users can freely select from, not all features are necessarily relevant for characterizing a specific phenotype of interest.“

1.3. The authors claim that the "lack of user-independent" segmentation is a major bottleneck today. It is unclear what they actually mean by that, but in any case, there is a plethora of fully automatic and extremely powerful segmentation methods out there that do not require any user interaction.

This is totally in line with our impression on the current state-of-the-art methods, but all of these recent segmentation methods only work well if they are first fed with sufficient training data. Particularly, the non-classical deep learning methods suffer from this problem. In addition, we would like to emphasize that we indeed do use the state-of-the-art segmentation method but only provide an additional way to further improve the tracking results in rare cases where the deep learning-based method fails. With the criticized sentence, we wanted to stress that CellCognition or CellProfiller, for instance, require selection of segmentations parameters, which have to be manually optimized for each experiment/image set and this generates inter-experimenter variability in the classifiers/extracted features.

2. MATLAB: The presented tool is very close to CellCognition and CellProfiler, both implemented in Python. This comes with several advantages, as the major Deep Learning libraries are also available in Python. The move to MATLAB (which is not an open-source solution) seems surprising.

We certainly agree that Python is becoming more and more popular, particularly in the realm of deep learning. However, as the segmentation part is just a preprocessing step in our pipeline and the main strengths of LiveCellMiner are the data analysis, data mining, visualization and statistical analysis capabilities, we decided to implement it as an extension of the MATLAB toolbox SciXMiner. The clear advantage of MATLAB here is that we can easily extend the software and have a wide variety of machine learning, statistical analysis, visualization etc. available that would have to be solved by a bunch of additional dependencies in Python. Moreover, we feel that getting a Python environment to run properly may be more challenging for inexperienced users than installing MATLAB that usually works right out of the box on any operation system taking care of correct versions of the required dependencies. Finally, MATLAB is (at least as for now) still available at most universities via campus licenses and it is thus useable by a large portion of academic employees.

3. Presented segmentation method (LoG filter, Otsu, Watershed): these are classical methods for nuclei and cell segmentation, and it has been shown many times that with multiple instance segmentation using Deep Learning, we can get much better results. The authors have compared their method in Table S1, but to my understanding, the authors have not carefully adapted the size parameter of CellPose (this is critical indeed). In our experience, CellPose always performs better than the classical method proposed by the authors. However, I understand that mitotic figures can be problematic on that account, but then, I find the solution to use a LoG filter (i.e. a return to classical image analysis) unsatisfying.

We generally agree that state-of-the-art deep learning methods outperform classical methods by a large margin if trained with sufficient data. However, as you correctly identify this is not always the case for rare events like cell divisions that additionally exhibit a large variety of different appearances that are hard to sufficiently represent in a single training set. The segmentations obtained by Cellpose are close to perfect for most of the cells and the used diameter parameter of 30 was already below the average diameter of our cells (~40 pixels). Further lowering this parameter would in turn result in over-segmentation of larger cells (which are the majority present in the screen). Moreover, cells in metaphase and anaphase are mostly detected correctly. We thus don’t think the problem can be resolved solely by adjusting the diameter parameter. In some rare cases, however, Cellpose tends to produce “flickering” segmentations for cells that undergo mitosis, *i.e.*, cells are lost for one or more frames causing interrupted and thus unusable tracks. The benefit of a classical fallback option is that it works without the need for new training data and out-of-the-box on new images as well. While Cellpose segmentations are generally preferred in our processing pipeline, in rare situations when Cellpose fails we resort to a classical method. We adapted the paragraph containing this description and added a new supplementary figure, to make the rationale of the fallback option clearer:

“Although Cellpose generally provides highly accurate segmentation results for the majority of the cells, we found that directly using Cellpose to replace the LoG-based object detection yielded less reliable tracking results. In some cases, Cellpose failed to properly segment rarely occurring cell shapes observed in meta- and anaphase. Thus, we additionally included a fallback option for cases where segmentations provided by Cellpose were missing and in these cases occasionally switch back to the classical segmentation method for individual cells. A quantitative assessment of the segmentation and tracking quality is provided in Table S1 and a qualitative demonstration of the detection and segmentation accuracy as well as exemplary erroneous detections are depicted in Fig S11”.

Moreover, we added a new supplementary figure to illustrate the rarely occurring issues of the Cellpose algorithm:

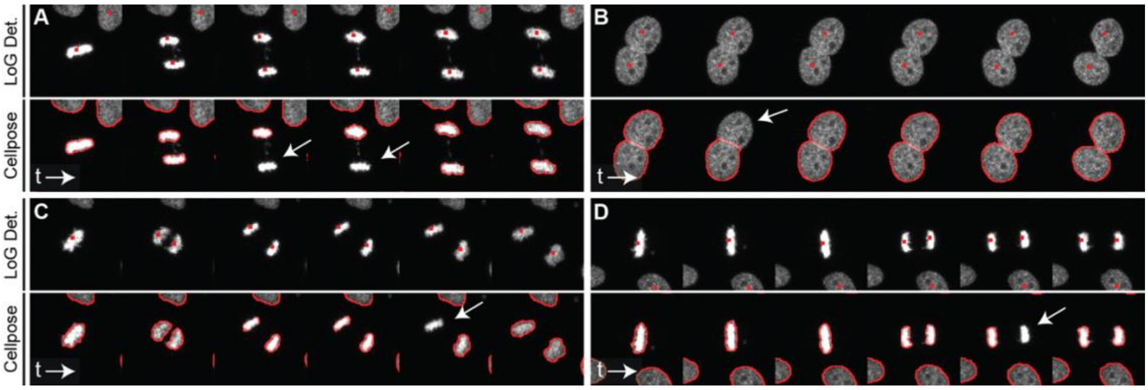

Fig. S11 Illustration of the LoG-based Nucleus Detection and Cellpose Segmentation*.* *Cellpose provides highly accurate nuclei segmentation for most of the cells. However, in some rare cases (e.g., in late anaphase), cells tend to flicker and remain undetected for one or more frames. To prevent interrupted tracks for such misdetections, LiveCellMiner provides a fallback option on classical image analysis methods and uses a LoG-based nucleus detection coupled with a classical binary threshold and watershed-based segmentation as detailed in the main text. The depicted examples qualitatively demonstrate the accurate segmentation performance of Cellpose and a few examples where detections were missed that are successfully identified by the classical LoG-based detection method. We found that using both approaches in combination resulted in complementary results and effectively in more complete tracks as quantitatively demonstrated in Table S1. The average diameter of all cells and across all time points in this example is 40.79 pixels and the Cellpose diameter parameter was set to the default value of 30 pixels, to allow segmenting smaller objects like cells in meta- and anaphase as well.*

References:

Antal, B.; Chessel, A.; Carazo Salas, R. E. Mineotaur: A Tool for High-Content Microscopy Screen Sharing and Visual Analytics. Genome Biol 2015, 16 (1), 283. https://doi.org/10.1186/s13059-015-0836-5.

Held, M.; Schmitz, M.; Fischer, B.; Walter, T.; Neumann, B.; Olma, M.; Peter, M.; Ellenberg, J.; Gerlich, D. CellCognition: Time-Resolved Phenotype Annotation in High-Throughput Live Cell Imaging. Nature methods 2010, 7 (9), 747–754. https://doi.org/10.1038/nmeth.1486.
