# Supplementary material for: LiveCellMiner: A new tool to analyze mitotic progression
Source: PLoS One. 2022 Jul 7;17(7):e0270923. doi: 10.1371/journal.pone.0270923 (PMC9262191; doi:10.1371/journal.pone.0270923)
Supplement: Attachment — Submitted filename: ResponseToReviewer.pdf [file pone.0270923.s025.pdf]

We thank the reviewer for the valuable feedback on our manuscript. Please find the point-by-point response to all raised concerns below:

Reviewer #1: In this manuscript, Moreno-Andrés et al. describe an open-source, automated software tool designed to quantify cell-cycle progression using live-cell imaging data called LiveCellMiner. The main goal of this manuscript is to show that LiveCellMiner can be used to accurately measure the cell-cycle using live-cell imaging data across various platforms and conditions. Furthermore, the authors demonstrate that LiveCellMiner reproduces previously characterized data in cells depleted of various cell-cycle proteins including PP2A, LSD1, and RecQL4. Lastly, the authors adapt their software to analyze data generated from high-content screens.

This manuscript generally demonstrates the broad capabilities of this software to accurately analyze live-cell data. The demonstrated reproducibility across various microscopy platforms will increase the adoption by the scientific community. The software builds on many pre-existing methods. While the novelty of this software is lacking in this regard, the utility will ultimately be determined by the users. With the enhancements of this software to make it more user-friendly, I find it to be of great value to the scientific community.

Altogether, I find that LiveCellMiner will be a very useful tool for analyzing cell-cycle progression using live cell imaging data. Specific comments:

1. The authors find that LiveCellMiner detected nuclear morphology phenotypes in LSD1 and PP2A depleted cells (line 331). This unexpected discovery needs to be validated to prove that the software accurately measures this phenotype. Because of this, the ability to use LiveCellMiner to uncover new phenotypes could be strengthened by showing that the abnormal nuclear shape detected by LiveCellMiner is observed in LSD1 and PP2A depleted cells.

   We added a manual analysis of the observed phenotypes in an additional supplementary figure (Fig S13):

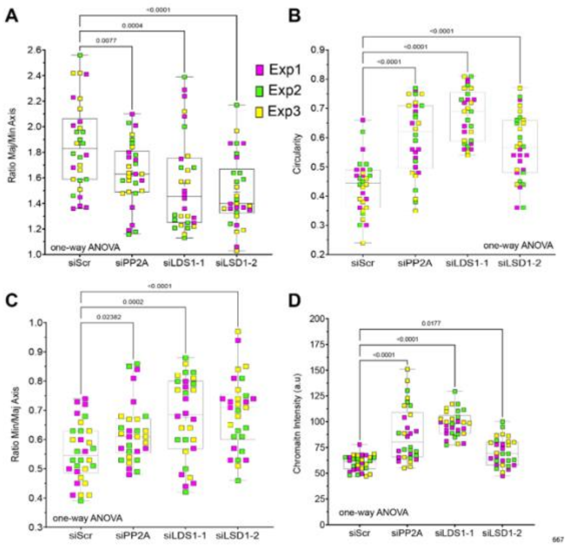

Fig. S13 Manual Validation of Unexpected Phenotypes in LSD1 and PP2A Depleted Cells. To confirm the unexpected nuclear morphology phenotypes in LSD1 and PP2A in late mitosis detected by LiveCellMiner, we manually measured the major/minor axes (A, C), circularity (B) and the mean intensity of cells (D) from the original LSD1 data set records 45h post transfection. Using Fiji [75], 10 random daughter cells 30 min after anaphase onset per condition per experiment (three independent experiments indicated in coloured data points) were manually segmented with the Wand tool in legacy mode, and the values were extracted using the ROI manager. The data were organized in excel tables and plots (box and whisker min to max) and statistical analysis were done using GraphPath (D'Agostino-Pearson normality test and parametric one-way ANOVA).

Moreover, we added the following statement in the main text: “A manual confirmation of the observed phenotypes detected by LiveCellMiner is provided in Fig S13 and the same effects were observed in an independent experiment with RPE cells as shown in Fig S9.”

2. I found the visualization options to be unclear with respect to the X-axis. Specifically, Fig.2 A-C, and similar figures. The work could benefit from a clear description of the X-axis and the vertical dotted lines in the legend. At least one description with corresponding images would be useful in the main text. (e.g. Fig.S12 paired with a graph).

Thanks for pointing this out. We extended Fig. S12 accordingly and reference it in the text at the first occurrence of temporal feature plots that use this axis formatting. The following figure and legend were added:

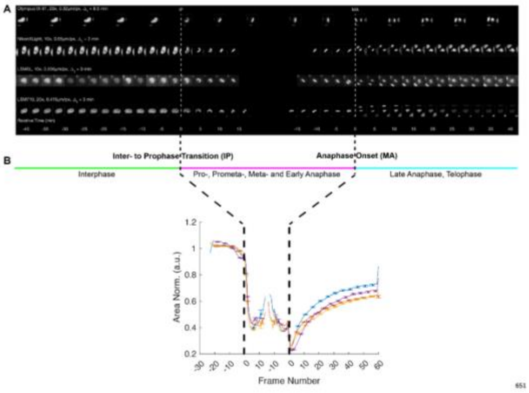

Fig. S12 Examples of Temporally Aligned Cells from Different Experiments and Time Line Explanations. (A) Each row shows a single cell of different experiments (see legend in the figure for imaging details). Images were scaled to a consistent size, temporally aligned and contrast was adapted for better visibility. (B) In all temporal plots of the manuscript, we group the visualization into three different phases, (1) interphase, (2) pro-, prometa-, meta- and early anaphase and (3) late ana- and telophase. The dashed lines indicate the interphase to prophase transition (IP) and the metaphase to anaphase transition/anaphase onset (MA). For better visibility and to be able to visualize cells with different IP to MA duration, the frames between IP and MA are evenly distributed to the left and right. Time stamps are in minutes and display the relative timing with respect to the transition time points.

3. The authors need to clearly state the types of cell lines that are used in the analysis. It is not until the comparison of Hela to RPE cells that it is implied that the presented results are mammalian cells. Is this software only limited to mammalian cells or can this be used for other species commonly used for cell-cycle research such as S. cerevisiae or S. pombe? Addition of this information would be useful to the scientific community.

Thanks for spotting this. We added the additional information about the cell lines to the introduction and extended the conclusions with a statement about the potential applicability to other cell types. Specifically, we added the following changes:

“Here, we reanalyze published and unpublished data sets of human tissue culture cells passing mitosis generated with different live-cell imaging platforms showing high reproducibility between the systems.”

“Although LiveCellMiner's synchronization tools were developed for recognizing mitotic morphologies of mammalian cells, the recognition of mitotic transitions in other organisms is possible. For instance, the analysis of mitotic progression of yeast could be easily implemented in the code, given that the shape of the chromatin mass is very particular at anaphase and could be supported by measurements of the position of the nucleus with respect to the yeast walls.”

4. What are the limitations in the data input in LiveCellMiner? The author’s comment: Line 87 “LiveCellMiner expects time series of 2D images of cells with chromatin-labeled nuclei and optionally additional markers in other channels.” Additional information about what the constraints on the input data would benefit those deciding to use the software.

LiveCellMiner can handle one additional channel and extracts intensity-related features based on the nucleus mask from the second image channel. The images need to have the same size and the same number of frames. We adapted the statement in the main text as follows to make the requirements clearer:

“LiveCellMiner expects time series of 2D images (2D+t) of cells with chromatin-labeled nuclei as the primary and required input. For two-channel experiments, LiveCellMiner can optionally extract additional intensity-related features based on the nucleus segmentation. Both channels need to have the same number of frames, the same image dimensions and need to be aligned properly.”

5. Fig 5 title should also include PP2A.

Done.
